# Exposure-driven tolerance modeling of efpeglenatide-induced nausea or vomiting using repeated time-to-event analysis: implications for dose-escalation strategies

**DOI:** 10.3389/fphar.2026.1846164

**Published:** 2026-09-22

**Authors:** Jiyoung Seo, Seungchan Choi, NaYoung Kim, Hyeeun Kim, Hyeong-Seok Lim

**Affiliations:** 1 Asan Medical Center, Department of Clinical Pharmacology and Therapeutics, University of Ulsan College of Medicine, Seoul, Republic of Korea; 2 Hanmi Pharm. Co., Ltd., Seoul, Republic of Korea

**Keywords:** adverse events, efpeglenatide, GLP-1 agonist, HM11260C, nausea or vomiting, NONMEM^®^, repeated time-to-events, tolerance

## Abstract

**Introduction:**

Nausea or vomiting are common adverse events (AEs) associated with glucagon-like peptide-1 receptor agonists and often decline over time despite continued exposure, suggesting tolerance development. However, the longitudinal dynamics of recurrent events and tolerance have not been quantitatively characterized for efpeglenatide. This study aimed to characterize efpeglenatide-induced nausea/vomiting in non-diabetic patients with obesity using repeated time-to-event (RTTE) modeling and to explore its implications for dose-escalation strategies.

**Methods:**

Data from six clinical trials were pooled, and 678 participants with obesity and/or type 2 diabetes mellitus were included in the analysis. A total of 672 nausea or vomiting events were analyzed using a sequential PK–AE approach based on a previously developed population pharmacokinetic model. Separate RTTE models were developed for an obesity population and a pooled total population. The models incorporated baseline hazard, a time-decaying non-drug effect, and an exposure-driven drug effect with tolerance linked to cumulative exposure (AUC). Model performance was evaluated using bootstrap and visual predictive checks, and Monte Carlo simulations were performed to assess nine dose-escalation scenarios. The obesity model served as the primary model for predicting dosing regimen–dependent AE patterns, whereas the pooled total model was used as an exploratory model to assess the concentration–AE response relationship.

**Results:**

Among the 678 participants, 247 (36.4%) experienced at least one episode of nausea or vomiting. In the obesity model, the exposure–hazard relationship was best described by a log-linear function. The estimated AUC associated with 50% tolerance development was 109,872 h ng/mL in the obesity model. Among the nine dosing scenarios, the Case 1–4 titration regimens achieved approximately 50% tolerance within 3–4 weeks while maintaining a lower early AE burden than high fixed-dose regimens. Low fixed-dose regimens delayed tolerance development, whereas high fixed-dose regimens increased the early AE burden.

**Conclusion:**

Efpeglenatide-associated nausea or vomiting was well characterized using an exposure-driven RTTE framework, and early cumulative exposure appeared to play a central role in tolerance development. These findings suggest that, in non-diabetic patients with obesity, stepwise titration of efpeglenatide may help optimize tolerability by mitigating early AE burden while allowing sufficient cumulative exposure for tolerance development.

## 1 Introduction

Glucagon-like peptide-1 receptor agonists (GLP-1 RAs) are widely used for the treatment of type 2 diabetes mellitus (T2DM) and obesity owing to their beneficial effects on glycemic control, weight reduction, and cardiovascular risk factors (Müller et al., 2019; Drucker, 2022). Despite their clinical benefits, gastrointestinal (GI) adverse events (AEs), such as nausea or vomiting, are the most commonly reported side effects associated with GLP-1 RAs and are a major cause of treatment discontinuation, especially during the early treatment period (Müller et al., 2019). These AEs are mechanistically associated with delayed gastric emptying and reduced GI motility induced by GLP-1 receptor activation (Müller et al., 2019; Holst, 2007). Notably, the incidence of nausea or vomiting is typically highest during the early phase of treatment and gradually decreases over time despite continued drug exposure (Drucker, 2022). This finding suggests the development of physiological adaptation or tolerance to the drug (Drucker, 2022; Holst, 2007).

Efpeglenatide is a long-acting GLP-1 receptor agonist developed for the treatment of type 2 diabetes and obesity (Reid et al., 2019; Pratley et al., 2019). GLP-1, an incretin hormone secreted by intestinal L-cells in response to food intake, exerts its effects through GLP-1 receptors, stimulating glucose-dependent insulin secretion, suppressing glucagon release, slowing gastric emptying, and reducing appetite through central mechanisms (Reid et al., 2019). Compared with once-daily GLP-1 receptor agonists such as liraglutide, efpeglenatide has a relatively longer half-life, enabling less frequent dosing and potentially improving treatment adherence (Rosenstock et al., 2019). The prolonged half-life of efpeglenatide is primarily attributed to reduced renal clearance (CL) and vascular endothelial uptake (Yoon et al., 2020). Similar to other GLP-1 receptor agonists, GI AEs remain among the most common AEs associated with efpeglenatide treatment (Pratley et al., 2019; Rosenstock et al., 2019).

Although numerous clinical studies have reported the incidence of nausea or vomiting associated with GLP-1 receptor agonists, most studies have focused on overall incidence rates rather than the longitudinal dynamics of recurrent AEs and the development of tolerance over time (Wharton et al., 2022a; Jastreboff et al., 2022). In particular, the relationship between drug exposure, cumulative exposure (AUC), and tolerance development has not been quantitatively characterized in most studies (Tang et al., 2024; Niebecker et al., 2013; Li et al., 2024). Accordingly, recurrent AEs, such as nausea or vomiting, require modeling approaches capable of describing repeated events and time- and exposure-dependent changes in risk (Karlsson et al., 2011; Zingmark et al., 2005). Conventional time-to-first-event analyses or simple incidence summaries are insufficient to capture these longitudinal dynamics (Karlsson et al., 2011; Karlsson et al., 2019).

Therefore, this study aimed to characterize the longitudinal dynamics of efpeglenatide-associated nausea or vomiting in non-diabetic patients with obesity using a repeated time-to-event (RTTE) modeling approach, provide a biologically plausible, exposure-based interpretation of cumulative exposure-driven tolerance development, and evaluate evidence-based titration strategies to improve tolerability in this population. In addition, an exploratory total model was developed using the pooled dataset including both patients with obesity and patients with type 2 diabetes mellitus (T2DM) and was used to complementarily explore the relationship between drug concentration and nausea/vomiting risk.

## 2 Methods

To ensure consistency in terminology across the analyses, the models in this study were defined as follows. The obesity model refers to the model developed in the non-diabetic obesity population and was used as the primary model for the main interpretation of this study. The total model refers to the model developed using the pooled dataset including both patients with obesity and patients with type 2 diabetes mellitus (T2DM). This model should not be interpreted as representing a pure T2DM population; rather, it was interpreted as an exploratory model to describe the overall AE pattern in the pooled dataset.

Detailed information on the study populations and obesity-related eligibility or exclusion criteria for each study is provided in Table 1. In the T2DM studies, BMI-related eligibility criteria were applied; however, these criteria did not explicitly exclude patients with obesity or stratify patients by obesity status. Therefore, the T2DM studies may have included both patients with T2DM with obesity and those with T2DM without obesity, and the available data did not support a reliable classification into “T2DM with obesity” and “T2DM without obesity.” In contrast, HM-EXC-205 enrolled non-diabetic patients with obesity and explicitly excluded diabetes mellitus. Accordingly, the obesity model was developed as the primary model to evaluate the longitudinal nausea/vomiting pattern and cumulative AUC-driven tolerance in non-diabetic patients with obesity.

**TABLE 1:** Summary of study populations, participant numbers, BMI-related eligibility criteria, and T2DM-related exclusion criteria across the six clinical studies.

Study	Number of participants			Study population	BMI-related eligibility criteria	Obesity-related exclusion criterion?	Interpretation for current modeling
	Total	Efpeglenatide	Placebo				
HM-EXC-102	34	26	8	Subjects with T2DM receiving a stable dose of metformin for ≥3 months or stable sulfonylurea plus metformin combination therapy for ≥3 months, with possible sulfonylurea washout	BMI ≥18 and ≤45 kg/m^2^ at screening	No explicit obesity exclusion identified	T2DM study, but obesity was not excluded; participants with obesity could have been included.
HM-EXC-201	48	40	8	Adult patients with T2DM receiving diet/exercise therapy only, stable oral antidiabetic medication, or metformin plus sulfonylurea combination therapy, with stable treatment for 2 months before screening and a 14-day washout of glucose-lowering drugs before dosing	BMI 25–40 kg/m^2^, inclusive	No explicit obesity exclusion identified	The BMI eligibility criterion required a BMI of 25–40 kg/m^2^, corresponding to overweight or obese BMI ranges; therefore, a non-overweight/non-obese T2DM subgroup could not be defined.
HM-EXC-202	68	51	17	Subjects with T2DM receiving stable metformin monotherapy for ≥3 months	BMI 27–45 kg/m^2^ at screening; BMI-based PK stratification was planned (27–37 vs. >37 kg/m^2^).	No explicit obesity exclusion identified	T2DM study in which obesity was not excluded; BMI eligibility criteria suggest that participants within obese BMI ranges could have been included.
HM-EXC-203	118	81	37	Subjects with T2DM receiving diet and exercise therapy with or without metformin monotherapy for ≥3 months; metformin users were required to receive a stable dose for ≥3 months	BMI <40 kg/m^2^ at screening	No explicit obesity exclusion identified	T2DM study with upper BMI limit only; obesity was not excluded.
HM-EXC-204	145	95	50	Subjects with inadequately controlled T2DM despite stable metformin monotherapy for ≥3 months prior to screening	BMI <40 kg/m^2^ at screening	No explicit obesity exclusion identified	T2DM study with upper BMI limit only; obesity was not excluded.
HM-EXC-205	265	205	60	Non-diabetic subjects with obesity; HbA1c ≤6.5% required at screening	BMI ≥30 kg/m^2^, or BMI ≥27 kg/m^2^ with treated or untreated obesity-related comorbidities such as hypertriglyceridemia, dyslipidemia, hypertension, glucose intolerance, or sleep apnea, at screening; participants with BMI >42 kg/m^2^ were excluded.	Participants with obesity were included; diabetes mellitus (T1DM, T2DM, or other) was explicitly excluded.	This study provides the clearest non-diabetic population with obesity and was therefore used as the primary basis for the obesity model.
Total	678	498	180	—	—	—	—

The values in the ‘Number of participants’ columns represent participants included in the RTTE analysis dataset. Studies 102–204 included participants with T2DM with undetermined obesity status, whereas study 205 included non-diabetic participants with obesity. Totals represent the sum across studies.

### 2.1 Data

#### 2.1.1 Clinical trial data sources and study characteristics

This analysis integrated data from six clinical trials (one Phase 1 and five Phase 2 studies) conducted to evaluate the pharmacokinetics (PK), pharmacodynamics (PD), and efficacy of efpeglenatide, with a specific focus on nausea or vomiting (Pratley et al., 2019; Rosenstock et al., 2019; Yoon et al., 2020; Del Prato et al., 2020; Hompesch et al., 2021).

Among these, one study used a single-dose design, while the remaining five utilized multiple-dose designs. Dosing frequencies varied across studies, including weekly, biweekly (every 2 weeks), and monthly regimens. In the multiple-dose studies, efpeglenatide doses ranged from 0.3 to 16 mg, whereas the single-dose study evaluated doses between 2 and 100 μg/kg. Three of the studies incorporated dose titration to reach the maintenance dose, while others were conducted without titration.

The study population comprised participants with type 2 diabetes mellitus (T2DM), in whom obesity status was not consistently distinguished, and non-diabetic participants with obesity. Across the six clinical trials, 917 participants received study treatment, of whom 688 received efpeglenatide, 180 received placebo, and 49 received liraglutide. Detailed information on the study populations, participant numbers, BMI-related eligibility criteria, and T2DM-related exclusion criteria is summarized in Table 1. Dosing regimens and protocol-defined follow-up periods are summarized in Table 2.

**TABLE 2:** Summary of dosing regimens and protocol-defined follow-up periods across the six clinical studies (Pratley et al., 2019; Rosenstock et al., 2019; Yoon et al., 2020; Del Prato et al., 2020; Hompesch et al., 2021).

Study	# Of dosing	Dosing frequency	Dosing regimen	Follow up period (day)
102	Multiple	Weekly	6 mg weekly (four times)	59 (±1)
			No titration	
		Monthly	16 mg monthly (three times)	123 (±1)
			4 mg (2 weeks) → 8 mg (2 weeks)	
201	Single	Single	2/4/8/14/20/40/60/100 μg/kg	82 (±4)
			No titration	
202	Multiple	Weekly	1/2/4 mg weekly (eight times)	106 (±4)
			No titration	
		Monthly	8/12/16 mg monthly (three times)	
			No titration	
203	Multiple	Weekly	0.3/1/2/3/4 mg weekly (12 times)	127 (±3)
			No titration	
204	Multiple	Monthly	8/12/16 mg monthly (two times)	155 (±3)
			4 mg (4 weeks) → 8 mg (1 week)	
205	Multiple	Weekly	4/6 mg weekly (20 times)	183 (±3)
			No titration	
		Biweekly	6/8 mg biweekly (9 times)	
			4 mg (2 weeks)	

In HM-EXC-102 and HM-EXC-203, liraglutide was included as an open-label active comparator and is not shown in the efpeglenatide dosing regimen summary. Single indicates single-dose administration, whereas Multiple indicates repeated dosing. Weekly indicates once every week, biweekly indicates once every 2 weeks, and monthly indicates once every 4 weeks. The arrow (→) indicates dose escalation during the titration period. For study 201, different dose levels were administered as single-dose cohorts. Follow-up periods are based on the protocol-defined study schedule. Actual last observation or follow-up times in the RTTE analysis dataset could be later than the protocol-defined period for some participants because of delayed visits or extended follow-up.

#### 2.1.2 Analysis population and RTTE data preparation

A total of 678 participants were included in the final analysis. Participants were excluded if they did not receive the study drug, discontinued the study prematurely, or lacked plasma concentration data despite being assigned to a treatment group. Individual predicted PK parameter estimates were used for participants with at least one measurable PK sample (Choi et al., 2025); conversely, those in the treatment group without any measurable PK concentrations were excluded from the analysis.

All AEs occurring from the initial dose until the final follow-up visit were included in the RTTE modeling. AEs occurring before the first administration were not considered treatment-emergent and were excluded. AEs reported on the first day of treatment were assumed to have occurred post-dose and were included in the analysis. For all participants, the last follow-up visit was defined as the censoring date, and any events occurring after this date were excluded. The baseline was set at the time of the first drug administration. Subsequent dosing and AE occurrence times were calculated as relative hours from this baseline. Events with reported exact times were treated as exact-time events. For events where only the date was available, particularly in study HM-EXC-202, they were treated as interval-censored (Seo, 2024; Seo and Lim, 2026). In these cases, the interval start was defined by the AE occurrence date, and the end time was set 24 h later. Given that nausea or vomiting were modeled simultaneously, simultaneous occurrences were separated by adding a minimal interval of 0.1 h to one of the events.

### 2.2 Software

All PK/PD (AE) modeling and parameter estimation were performed using nonlinear mixed-effects modeling implemented in NONMEM (version 7.5; ICON Development Solutions, Hanover, MD, United States) (Beal et al., 1989). The first-order conditional estimation method with interaction (FOCEI) was used to approximate the marginal likelihood and facilitate estimation of population model parameters (Lindstrom and Bates, 1990; Bauer, 2019a; Bauer, 2019b). Data processing and graphical visualizations were conducted using R software (version 4.4.0 or later; R Foundation for Statistical Computing) (R Core Team, 2022).

### 2.3 Nausea or vomiting model strategy and structure model

#### 2.3.1 Modelling strategy

The population PK model of efpeglenatide was adopted from Choi et al., who established the model using pooled data from six clinical trials in patients with T2DM and individuals with obesity (Choi et al., 2025). The final PK structure comprised a linear two-compartment disposition model with first-order absorption and elimination. To characterize the double peaks observed during the absorption phase, a dual-absorption-pathway structure was incorporated. In the final PK model, body weight was included as a covariate on CL and the absorption rate constant (Ka), whereas T2DM and obesity status were included as covariates on CL. These covariate effects resulted in differences in drug exposure between individuals with T2DM and those with obesity. To account for these exposure differences in the exposure–AE relationship, two separate AE models were developed: an obesity model based on non-diabetic participants with obesity and a total model based on the pooled total population. Each AE model was constructed using pooled data from both placebo and active treatment groups.

The relationship between drug exposure and AEs was evaluated using a sequential PK-AE modeling approach. Individual PK parameters (IPK) obtained from the final PK model were fixed during the construction of the AE model (Choi et al., 2025). Exposure metrics, such as plasma concentration and AUC, were then derived from these fixed estimates to characterize the drug effect on the log-hazard of AE occurrence (Niebecker et al., 2013; Li et al., 2024; U.S. Food and Drug Administration, 2023a).

The exposure–response relationship for efpeglenatide-induced nausea or vomiting was characterized using an RTTE model (Niebecker et al., 2013; Li et al., 2024; Karlsson et al., 2011). Unlike traditional time-to-first-event or Markov chain models frequently employed in AE analyses, our approach refined the widely used RTTE framework by redefining the conditional probability structure to better capture the inter-dependency between successive events (Tang et al., 2024; Karlsson et al., 2011; Zingmark et al., 2005; Karlsson et al., 2019; Del Prato et al., 2020; Hompesch et al., 2021; Choi et al., 2025; Seo, 2024; Seo and Lim, 2026; Beal et al., 1989; Lindstrom and Bates, 1990; Bauer, 2019a; Bauer, 2019b). This refined model was applied to describe the longitudinal patterns of event occurrence, with detailed mathematical derivations provided in Supplementary Material S1 (Seo, 2024; Seo and Lim, 2026). The RTTE modeling framework applied in this study has been described in greater methodological detail in a companion manuscript currently under review in this journal.

The occurrence of nausea or vomiting was characterized by an RTTE model consisting of three structural components: baseline log-hazard, a non-drug effect, and a drug effect. For the base model, an exponential distribution was assumed, implying a constant baseline hazard over time—an approach deemed appropriate for capturing the stochastic nature of nausea or vomiting. The log-hazard ( $\log\left(h\left(\theta ;t\right)\right)$ ) of the parametric RTTE model was defined as shown in Equation 1 (Tang et al., 2024; Niebecker et al., 2013; Li et al., 2024; Karlsson et al., 2011; U.S. Food and Drug Administration, 2023a; Gabrielsson and Weiner, 2016):

$$\log\left(h\left(\theta ;t\right)\right)=\log\theta_{base}+f_{nd}\left(\theta ;t\right)+f_{d}\left(\theta ;t,C_{p},Tol\right)$$ (1)

Where $h\left(\theta ;t\right)$ denotes the hazard governed by the fixed-effect parameter vector θ at time t since the first dose. $\theta_{base}$ represents the baseline hazard, whereas $f_{nd}\left(\theta ;t\right)$ and $f_{d}\left(\theta ;t,C_{p},Tol\right)$ represent the non-drug and drug effects, respectively.

Non-drug effects encompass psychological and environmental factors, as well as lifestyle modifications integrated into clinical trials, such as dietary and exercise regimens. The maximum value for the non-drug effect was set at 1. The temporal dissipation of this effect ( $f_{nd}\left(\theta ;t\right)$ ) was modeled using an exponential placebo function to describe the pattern where the hazard is highest at the treatment’s onset and gradually declines over time, as shown in Equation 2:

$$f_{nd}\left(\theta ;t\right)=\exp\left(-\theta_{ktol}\cdot t\right)$$ (2)

The drug effect component ( $f_{d}\left(\theta ;t,C_{p},Tol\right)$ ) incorporated both the time-varying drug concentration (Cp) and tolerance development (Tol). Here, Cp denotes the time-varying drug concentration, and Tol represents tolerance development over time driven by cumulative drug exposure, expressed as AUC. During structural model development, several candidate exposure–hazard functions, including log-linear and $E_{\max}$ models, were evaluated separately for the obesity dataset and the pooled total dataset. The final structural model was selected by comprehensively considering convergence, objective function value (OFV), parameter precision, and VPC-based predictive performance. Finally, in the obesity dataset, the log-linear model best captured the rapid initial increase in exposure-related hazard followed by gradual attenuation over time; therefore, this structure was selected as the final model, as defined in Equation 3:

$$f_{d}\left(\theta ;t,C_{p},Tol\right)=tol\left(\theta ;t\right)\cdot Slope\cdot \log\left(C_{p}+Intercept\right)$$ (3)

In contrast, in the pooled total dataset, the total model was developed as a complementary analysis to evaluate the relationship between drug concentration and the risk of nausea or vomiting. Accordingly, the *E_max_* structure was selected to explore the possibility of pharmacological interpretation based on ${\;EC}_{50}$ and $E_{\max}$ , as defined in Equation 4:

$$f_{d}\left(\theta ;t,C_{p},Tol\right)=\frac{E_{\max}\cdot tol_{1}\left(\theta_{1};t\right)\cdot C_{p}}{EC_{50}\cdot tol_{2}\left(\theta_{2};t\right)+C_{p}}$$ (4)

In the pooled total model, several candidate structures were evaluated to determine the tolerance parameterization for $E_{\max}$ and ${\;EC}_{50}$ . The evaluated structures included dual tolerance on both $E_{\max}$ and ${\;EC}_{50}$ with shared ${TOL}_{\max}$ and ${TC}_{50}$ , dual tolerance with separate ${TOL}_{\max}$ and shared ${TC}_{50}$ , dual tolerance with separate ${TOL}_{\max}$ and separate ${TC}_{50}$ , and single-component tolerance applied only to either $E_{\max}$ or ${\;EC}_{50}$ . The final pooled total model used separate maximum tolerance effects for $E_{\max}$ and ${\;EC}_{50}$ , while sharing ${TC}_{50}$ , the cumulative AUC scale at which 50% of tolerance develops. This structure was considered to allow different magnitudes of tolerance effect on $E_{\max}$ and ${\;EC}_{50}$ while preserving parameter parsimony. The comparison of candidate structures is presented in Supplementary Materials S2 and S3.

Detailed descriptions of all model parameters are provided in Supplementary Material S4 (Tang et al., 2024; Niebecker et al., 2013; Li et al., 2024; U.S. Food and Drug Administration, 2023a; Gabrielsson and Weiner, 2016). Figure 1 illustrates the RTTE model structures for obesity-only and pooled total populations, respectively.

**FIGURE 1:**
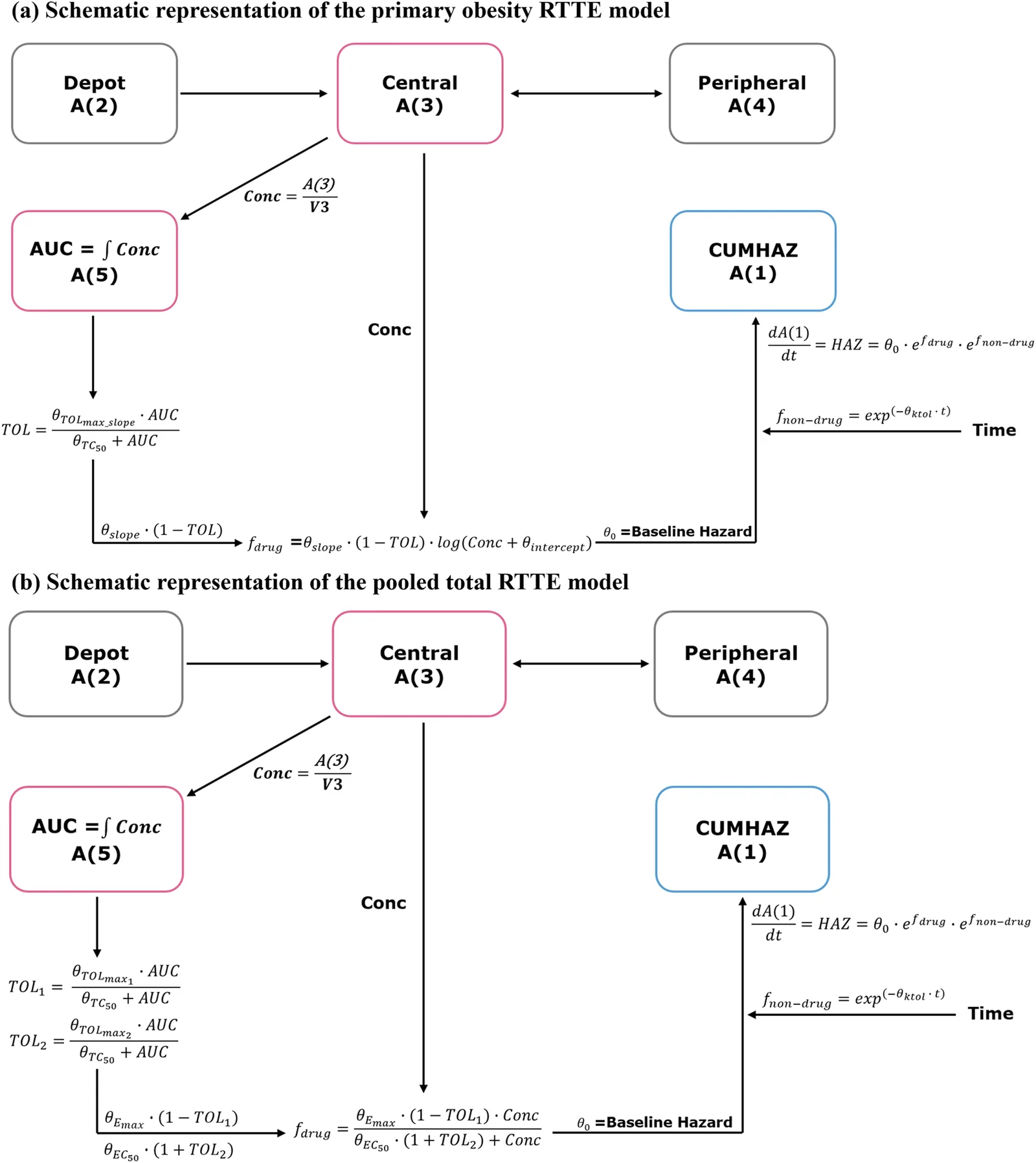
Schematic representation of the final RTTE models for nausea/vomiting. The pharmacokinetic model consists of depot (A(2)), central (A(3)), and peripheral (A(4)) compartments. Drug concentration in the central compartment drives the drug effect on the event hazard. V3 denotes the central compartment volume, such that Conc = A(3)/V3. Cumulative exposure (AUC, A(5)) is calculated as the integral of the central compartment concentration over time and is used to describe cumulative AUC-driven tolerance development. The event hazard is defined as the baseline hazard multiplied by the exponential drug-effect and non-drug-effect terms. The non-drug effect decreases exponentially over time, and the cumulative hazard is represented by CUMHAZ (A(1)), which governs repeated event occurrence in the RTTE framework. The drug-effect component differed between the primary obesity and pooled total models. In both models, the tolerance term was modeled as an E_max_-type function of cumulative AUC; however, tolerance was applied to different drug-effect parameters in the two models. **(a)** In the primary obesity model, the concentration–effect relationship was described using a log-linear structure, and cumulative AUC-driven tolerance was applied to the concentration–effect slope. **(b)** In the pooled total model, the concentration–effect relationship was described using an E_max_ structure, and cumulative AUC-driven tolerance was applied separately to E_max_ and EC_50_.

To isolate and precisely characterize the natural course of nausea or vomiting in the absence of active drug treatment, a separate evaluation of the placebo group was conducted. The placebo model was developed using only the baseline hazard and the non-drug effect (Equation 2).

The baseline hazard ( $\theta_{0}$ ) and $\theta_{ktol}$ parameters were initially estimated in units of week^−1^ for both the obesity and total models. To facilitate the interpretation of these parameters, the estimated values were converted to h^−1^ by applying a scaling factor of 168 h (1 week).

#### 2.3.2 Covariates

Potential covariates were evaluated to account for inter-individual variability in the model parameters. Demographic characteristics (e.g., age, sex, race), clinical variables (glycated hemoglobin [HbA1c] and body mass index [BMI]), and laboratory measurements (bilirubin and creatinine) were assessed as clinically relevant factors. These covariates were evaluated against all base model parameters. The covariate models and their mathematical formulations are provided in Supplementary Material S5.

Covariate selection followed a stepwise procedure based on the likelihood ratio test (LRT). Forward inclusion was conducted at a significance level of p < 0.05 (ΔOFV >3.84), followed by backward elimination at p < 0.001 (ΔOFV >10.83) (Jonsson and Karlsson, 1998). To ensure model convergence and stability, reduced models or full models were constructed where necessary.

#### 2.3.3 Model evaluation

Model development was guided by successful convergence, a substantial reduction in the objective function value (OFV), and the precision of parameter estimates, as assessed by relative standard errors (RSEs) (Ette, 1997). Structural stability was monitored throughout the model-building process. The model was simplified by removing redundant parameters or *via* re-parameterization to ensure numerical stability (Bonate, 2011).

In cases where standard errors could not be obtained due to a covariance step failure, parameter uncertainty was assessed using a nonparametric bootstrap approach (Ette, 1997; Lindbom et al., 2005). A total of 1,000 bootstrap datasets were generated by resampling with replacement from the original dataset. Each replicate was fitted with the final model, and 95% confidence intervals (CIs) were derived from the 2.5th and 97.5th percentiles of the resulting empirical distribution.

The bootstrap median and 95% confidence interval were derived by including both minimization-successful replicates and evaluable replicates that terminated due to rounding errors but still produced parameter estimates. In the primary obesity model, a sensitivity analysis was conducted on the parameter showing a relatively wide confidence interval to evaluate the effect of parameter uncertainty on the model-predicted cumulative probability profile.

The predictive performance of the final RTTE model was evaluated using a Visual Predictive Check (VPC) (Karlsson and Holford, 2008; Bergstrand et al., 2011). Based on the final parameter estimates, 100 Monte Carlo simulation datasets were generated (Bonate, 2011), and model adequacy was assessed by comparing the observed Kaplan–Meier curve with the simulation-based 95% prediction interval (Bergstrand et al., 2011). The simulations reflected the dosing regimen, observed participants, and covariate distributions of each model, and the duration was set to 30 weeks for the obesity model and 46 weeks for the pooled total model.

Furthermore, cumulative event and event-free probabilities were calculated to verify that the proposed hazard model sufficiently characterized the temporal patterns and longitudinal occurrence of AEs. The time to reach specific thresholds ( ${TC}_{50}$ , ${TC}_{90}$ ), as well as $T_{max,ss}$ and $C_{max,ss}$ , were predicted by graphical method. More specifically, these estimated values were determined by identifying the intersection points between the target y-axis thresholds and the simulated median cumulative AUC-time profiles or the simulated median concentration-time profiles, based on the dosing regimens of the observed dataset (Choi et al., 2025).

${TC}_{90}$ values were derived from ${TC}_{50}$ using the $E_{\max}$ model relationship, and the TC , representing the cumulative AUC threshold associated with a target effect or tolerance level ( F ) was calculated using the following Equation 5 (Gabrielsson and Weiner, 2016).

$${TC}_{F}={\left(\frac{F}{100-F}\right)}^{\frac{1}{H}}\cdot {TC}_{50}$$ (5)

Where F represents the percent response and H denotes the Hill coefficient as 1. Since the Hill coefficient is 1, this equation simplifies to ${TC}_{90}=9\cdot \;{TC}_{50}$ .

#### 2.3.4 Simulation analyses to support clinical interpretation

Monte Carlo simulations were performed for multiple dosing scenarios using the AE model for nausea or vomiting (Bonate, 2011; Karlsson and Holford, 2008; Lalonde et al., 2007). A total of nine dosing scenarios (Table 3) were evaluated over 48 weeks. In this simulation, analyses were conducted while preserving each participant’s observed covariates and individual PK-derived exposure profiles from the source data.

**TABLE 3:** Simulation cases of the nausea or vomiting model.

Scenario	Dosing regimen (weekly)		
	Titration		Maintenance
Case 1	8 weeks	2, 2, 4, 4, 6, 6, 8, 8 mg	10 mg
Case 2	4 weeks	2, 2, 4, 4 mg	6 mg
Case 3	16 weeks	2, 2, 4, 4, 6, 6, 6, 6, 6, 6, 6, 6, 6, 6, 8, 8 mg	10 mg
Case 4	12 weeks	2, 2, 4, 4, 6, 6, 6, 6, 8, 8, 8, 8 mg	10 mg
1 mg weekly	—		1 mg
2 mg weekly	—		2 mg
10 mg weekly	—		10 mg
6 mg weekly	—		6 mg
8 mg weekly	—		8 mg

The titration column shows weekly doses administered during the titration period. Maintenance indicates the dose administered after the titration period. The 1 mg, 2 mg, 6 mg, 8 mg, and 10 mg weekly scenarios represent fixed-dose regimens without titration. The 1 mg and 2 mg weekly scenarios were included as low-exposure reference regimens, whereas the 6 mg, 8 mg, and 10 mg weekly scenarios were included as high-exposure reference regimens.

For each scenario, a total of 100 simulation replicates were performed, with each replicate consisting of 678 patients. The median and 95% prediction interval for the cumulative probability of nausea/vomiting and the cumulative event-free probability were derived from the simulated distributions and presented graphically (Seo, 2024; Seo and Lim, 2026). The time to reach predefined threshold values, including ${TC}_{50}$ and ${TC}_{90}$ , as well as $T_{max,ss}$ and $C_{max,ss}$ , was predicted based on the simulation results for each dosing scenario.

Dose-regimen simulations were conducted to evaluate the impact of titration strategies and maintenance doses on the predicted cumulative incidence of nausea/vomiting. Four candidate titration regimens (Cases 1–4; Table 3) were assessed as clinically feasible dose-escalation strategies. In addition, a once-weekly 1, 2, 6, 8, and 10 mg fixed-dose regimen was included as the reference scenario to compare predicted patterns under low- and high-exposure conditions. The once-weekly 1 and 2 mg regimens were included to assess adverse event profiles under low cumulative exposure and slow exposure-accumulation conditions, whereas the once-weekly 6, 8, and 10 mg regimens were included to assess rapid exposure accumulation and initial adverse event burden associated with high fixed doses.

The maintenance doses and escalation schemes presented in Cases 1–4 of Table 3 were designed based on the dose ranges evaluated in previous efpeglenatide clinical trials and efficacy-related simulations (Choi et al., 2025). The same initial titration design was applied during the first 6 weeks across Cases 1–4, whereas the duration of subsequent escalation steps and the final maintenance dose varied by case. Across all cases, dosing was structured to start from 2 mg once weekly, followed by stepwise escalation in 2 mg increments. This design was based on the model-informed interpretation that initiation at the lowest doses evaluated in previous clinical studies, such as 0.3 mg or 1 mg, could reduce the initial adverse event burden but may slow cumulative AUC accumulation and delay attainment of the cumulative exposure threshold required for tolerance development. Therefore, 2 mg was selected as a practical starting dose capable of inducing meaningful cumulative exposure accumulation without excessively increasing early exposure. The maintenance doses were set within the range of 6–10 mg once weekly by considering the weekly and monthly dose ranges evaluated in previous clinical studies, as well as their relevance to subsequent efficacy-related simulations (Choi et al., 2025). This enabled comparison, within a consistent dose-escalation framework (Cases 1–4), of how differences in escalation rate and maintenance dose affected the predicted cumulative occurrence probability of nausea or vomiting.

Additionally, for each dose-regimen scenario, the weekly model-predicted cumulative occurrence probability, $1-S\left(t\right)$ , was calculated by dose interval using the RTTE model–based event-free survival probability, $S\left(t\right)$ . Here, $1-S\left(t\right)$ represents the proportion of patients predicted to experience nausea/vomiting at least once up to the corresponding time point. The difference in $1-S\left(t\right)$ between consecutive weekly time points was calculated as the increment in the predicted cumulative occurrence probability for each dosing interval. This value represents the incremental proportion of patients predicted to experience nausea/vomiting for the first time during the corresponding interval. Because the simulations were based on the hazard and tolerance parameters estimated from the repeated nausea/vomiting event data in the RTTE model, these patient-level probabilities can be interpreted as reflecting the tendency for recurrent event occurrence while incorporating the cumulative exposure-driven tolerance effect. The weekly cumulative occurrence probabilities and dose-interval increments for the primary obesity model were summarized in Supplementary Material S6.

## 3 Results

### 3.1 Data summary

Among the 678 participants, 247 (36.4%) experienced at least one episode of nausea or vomiting. In the treatment group, 219 participants experienced at least one episode of nausea or vomiting, whereas 28 participants in the placebo group experienced at least one such event. Overall, 672 individual nausea or vomiting events were recorded, of which 628 occurred in the efpeglenatide treatment group and 44 occurred in the placebo group. The number of nausea or vomiting events by clinical study, as well as the number and proportion of participants who experienced at least one event, are summarized in Table 4. In addition, the proportion of events occurring at the corresponding dose/regimen among all events within each study is presented in Supplementary Material S7.

**TABLE 4:** Summary of nausea/vomiting events and affected participants across the six clinical trials.

Study	Number of nausea/vomiting events			Number of participants with nausea/vomiting (proportion of participants with ≥1 NV event)		
	Total events	Treatment events	Placebo events	Total participants with events	Treatment participants with events	Placebo participants with events
102	40	39	1	16 (0.471)	15 (0.577)	1 (0.125)
201	12	12	0	10 (0.208)	10 (0.250)	0 (0.00)
202	79	64	15	25 (0.368)	18 (0.353)	7 (0.412)
203	32	26	6	22 (0.186)	16 (0.198)	6 (0.162)
204	95	91	4	44 (0.303)	42 (0.442)	2 (0.04)
205	414	396	18	130 (0.491)	118 (0.576)	12 (0.20)
Total	672	628	44	247	219	28

T2DM, type 2 diabetes mellitus; NV, nausea or vomiting; Treatment refers to efpeglenatide-treated participants. When nausea/vomiting occurred concurrently, each was counted as a separate adverse event. Participants with nausea/vomiting were defined as participants who experienced at least one nausea or vomiting event. Total values represent sums across studies.

### 3.2 Model parameters

The two AE models adequately described the observed AE incidence. Final parameter estimates and bootstrap results for obesity and the pooled total models are summarized in Table 5.

**TABLE 5:** Parameter estimates for the final RTTE nausea or vomiting model in the obesity and total models.

Obesity model				
Parameter	Description	Unit	Estimates	Bootstrap median (95% CI)
Base model: Exponential model				
$\theta_{0}$	Baseline hazard	hr^−1^	0.000153	0.000169 (0.0000640–0.000351)
Drug effect (log-linear concentration model)				
$\theta_{Slope}$	Slope of the *log(concentration + intercept)* effect	(hr · ng/mL)^−1^	0.627	0.592 (0.302–0.825)
$\theta_{Intercept}$	Intercept of concentration effect	ng/mL	0.634	0.398 (0.000702–3.147)
Tolerance effect (AUC-driven)				
$\theta_{{TOL}_{\max\_\text{slope}}}$	Maximum tolerance effect on *Slope*	—	0.996	0.996 (0.996–0.996)
$\theta_{{TC}_{50}}$	AUC producing 50% of the maximum tolerance	hr · ng/mL	109,872	109,872 (109,872–109,872)
Non-drug effect				
$\theta_{ktol}$	Rate constant for non-drug effect	hr^−1^	0.0208	0.0191 (0.00219–0.0263)

Total model				
Base model: Exponential model				
$\theta_{0}$	Baseline hazard	hr^−1^	0.0000702	0.0000674 (0.0000381–0.000122)
Drug effect (Emax concentration model)				
$\theta_{\mathrm{Emax}}$	Maximum drug effect on log-hazard	—	5.22	3.622 (2.672–8.472)
$\theta_{{EC}_{50}}$	Concentration producing 50% of the maximum drug effect	ng/mL	81.0	0.102 (0.00083–307.315)
Tolerance effect (AUC-driven)				
$\theta_{{TOL}_{\max_{1}}}$	Maximum tolerance effect on $E_{\max}$	—	0.829	0.824 (0.616–0.996)
$\theta_{{TOL}_{\max_{2}}}$	Maximum tolerance effect on ${EC}_{50}$	—	0.996	0.996 (0.996–0.996)
$\theta_{{TC}_{50}}$	AUC producing 50% of the maximum tolerance	hr · ng/mL	109,872	109,872 (109,872–109,872)
Non-drug effect				
$\theta_{ktol}$	Rate constant for non-drug effect	hr^−1^	0.0117	0.00901 (0.00404–0.0147)

Where $\theta_{0}$ denotes the baseline hazard; $\theta_{ktol}$ is the first-order rate constant describing the time course of the non-drug effect; $\theta_{slope}$ is the slope of the log-linear concentration–effect relationship; $\theta_{intercept}$ is the intercept of the log-linear concentration–effect model; $\theta_{{TOL}_{\max}\_slope}$ represents the maximum tolerance effect on $\theta_{slope}$ ; $\theta_{\mathrm{Emax}}$ represents the maximum drug effect on the log-hazard; $\theta_{{EC}_{50}}$ is the efpeglenatide concentration producing 50% of the maximum drug effect; $\theta_{{TOL}_{\max_{1}}}$ and $\theta_{{TOL}_{\max_{2}}}$ represent the maximum tolerance effects on $\theta_{\mathrm{Emax}}$ and $\theta_{{EC}_{50}}$ , respectively; and $\theta_{{TC}_{50}}$ denotes the cumulative AUC producing 50% of the maximum tolerance effect for the corresponding tolerance function applied to *Slope*, $E_{\max}$ , or $EC_{50}$ , as applicable; Note that the bootstrap 95% CIs for $\theta_{{TOL}_{\max}\_slope}$ and $\theta_{{TC}_{50}}$ corresponding to *Slope*, $E_{\max}$ , or ${EC}_{50}$ , as applicable, showed minimal variance, indicating high precision in the estimation or the parameter reaching its boundary during the convergence process. Bootstrap median and 95% CI were calculated using evaluable replicates, defined as bootstrap runs with successful minimization or minimization terminated with rounding errors that yielded parameter estimates. Non-evaluable replicates were excluded from the bootstrap summary. In the obesity model, the log-linear drug effect was modeled as *Slope × log(concentration + intercept)*, and the tolerance effect was modeled as a function of cumulative exposure (AUC). In total model, the drug effect was modeled using an $E_{\max}$ model. Tolerance effects were modeled on both $E_{\max}$ and ${EC}_{50}$ parameters as functions of cumulative exposure (AUC).

Clinically relevant covariates, such as sex, age, and baseline BMI, were assessed for their potential effects. When several covariates were included, OFV decreased; however, the VPC did not show a meaningful improvement in predictive performance for the observed longitudinal profile of nausea/vomiting, particularly with respect to early event accumulation followed by attenuation or plateauing. Therefore, these covariates were not included in the final RTTE model.

This prioritization of VPC-based predictive performance was appropriate because the primary objective of this study was to predict the occurrence pattern of nausea/vomiting and the development of tolerance following efpeglenatide administration in non-diabetic participants with obesity, and to evaluate changes in adverse-event burden across dosing scenarios. Accordingly, VPC-based predictive performance that appropriately reproduced the observed event-time profile was considered more important in selecting the final model than a simple reduction in OFV.

These findings suggest that, rather than indicating the absence of effects of demographic or baseline characteristics such as sex, age, and BMI, some exposure-related effects may have already been captured at the population PK model stage through covariates retained in the PK model, including body weight on CL and Ka and T2DM/obesity status on CL (Choi et al., 2025). They also suggest that, within the current RTTE data structure, additional clinically meaningful independent covariate effects may have been difficult to identify robustly (Li et al., 2024).

#### 3.2.1 Obesity model

The baseline hazard ( $\theta_{0}$ ) was estimated to be 0.000153 h^−1^, and the non-drug (placebo) effect was governed by a first-order rate constant ( $\theta_{ktol}$ ) of 0.0208 h^−1^. The pharmacodynamic (PD) effect was characterized by a log-linear relationship, with the slope parameter ( $\theta_{slope}$ ) estimated at 0.627 (hr · ng/mL)^−1^. Tolerance development was incorporated as a function of AUC, where the AUC required to achieve 50% of the maximum tolerance effect ( ${TC}_{50}$ ) was estimated to be 109,872 h · ng/mL. The maximum fractional reduction in the slope ( $\theta_{TOL_{\max\_slope}}$ ) was estimated as 0.996, yielding a calculated slope of 0.00251 h · ng/mL^−1^ at the state of maximal tolerance.

In the obesity model, among the 1,000 bootstrap replicates, 957 (95.7%) terminated with successful minimization, and an additional 40 (4.0%) terminated due to rounding errors but provided evaluable parameter estimates. Accordingly, a total of 997 replicates (99.7%) were considered evaluable. When the results obtained using only successful replicates were compared with those obtained using evaluable replicates including both successful and rounding-error replicates, the bootstrap median and 95% confidence intervals were generally consistent. This indicates that the bootstrap results for the obesity model were broadly stable and were not materially affected by the replicate inclusion criteria. The summaries of the 95% confidence intervals according to each replicate inclusion criterion are presented in Supplementary Material S8, and the sensitivity graph for the intercept parameter, which showed a relatively wide 95% confidence interval, is presented in Supplementary Material S9.

#### 3.2.2 Total model

The baseline hazard ( $\theta_{0}$ ) was estimated to be 0.0000702 h^−1^ with the non-drug effect ( $\theta_{ktol}$ ) of 0.0117 h^−1^. The PD effect in the total model was characterized by an *E_max_* concentration–effect relationship, with *θ_Emax_* estimated at 5.22 and *θ*_*EC50*_ estimated at 81.0 ng/mL. The onset of tolerance resulted in a progressive attenuation of efficacy and potency; at the state of maximal tolerance, $E_{\max}$ was reduced to 0.893 (an 82.9% reduction), and ${EC}_{50}$ was shifted to 161.7 ng/mL (approximately 2-fold increase). This compensatory process was governed by a $\theta_{{TC}_{50}}$ of 109,872 h · ng/mL, representing the AUC at which half-maximal tolerance is reached.

In the pooled total model, among the 1,000 bootstrap replicates, 769 (76.9%) terminated with successful minimization. When replicates that were terminated due to rounding errors but provided evaluable parameter estimates were also included, a total of 993 replicates (99.3%) were considered evaluable. When the bootstrap medians and 95% confidence intervals calculated using all replicates, minimization-successful replicates, and evaluable replicates were compared, the tolerance-related parameters showed generally consistent and stable estimates. In contrast, the bootstrap confidence intervals for $E_{\max}$ and ${EC}_{50}$ were relatively wide, and the median ${EC}_{50}$ estimate, in particular, showed some differences depending on the replicate inclusion criterion. Therefore, $E_{\max}$ and ${EC}_{50}$ in the pooled total model were interpreted not as definitive pharmacological thresholds in a specific homogeneous patient population, but as exploratory indicators. In particular, the low final *EC*_50_ estimate suggests that drug-related adverse-event risk may develop rapidly after initial exposure. The parameter estimates and their 95% bootstrap confidence intervals, summarized according to the replicate inclusion criteria, are presented in Supplementary Material S10.

#### 3.2.3 Placebo model

The parameter estimates for the placebo and placebo plus treatment models are summarized in Table 6. Within the placebo cohort (Group A), the baseline hazard ( $\theta_{0}$ ) and non-drug effect parameters ( $\theta_{ktol}$ ) were comparable between the obesity (O) and total (T) populations.

**TABLE 6:** Comparison of baseline hazard and non-drug effect parameters between the obesity and total models under placebo and combined treatment conditions.

Parameter	Population	Placebo (A)	Unit	Placebo + treatment (B)	Unit	Ratio		Relative fold -Difference (O/T)	
						A/B	B/A	A/B	B/A
Baseline Hazard ( $\theta_{0}$ )	Obesity (O)	0.0000667	1/h	0.000153	hr^−1^	0.436	2.294	0.548	1.823
	Total (T)	0.0000558	1/h	0.0000702	hr^−1^	0.795	1.258		
	Ratio (O/T)	1.20	—	2.18	—	—	—		
Non-drug Effect ( $\theta_{ktol}$ )	Obesity (O)	0.0018	1/h	0.0208	hr^−1^	0.087	11.556	0.596	1.679
	Total (T)	0.0017	1/h	0.0117	hr^−1^	0.145	6.882		
	Ratio (O/T)	1.06	—	1.78	—	—	—		
Half-life of non-drug effect ( $t_{1/2}$ )	Obesity (O)	16.1	days	1.39	Days	11.5	0.09	1.679	0.596
	Total (T)	17.0	days	2.47	Days	6.88	0.15		
	Ratio (O/T)	0.94	—	0.56	—	—	—		
Time to 97% loss of the non-drug effect	Obesity (O)	2.67	months	6.94	Days	11.5	0.09	1.679	0.596
	Total (T)	2.83	months	12.34	Days	6.88	0.15		
	Ratio (O/T)	0.94	—	0.56	—	—	—		

O, obesity population; T, total population including T2DM; A, placebo; B, placebo + treatment; The half-life of the non-drug effect was calculated as $\frac{\log2}{\theta_{ktol}}$ , and the time to 97% loss of the non-drug effect was calculated as $5\cdot t_{1/2}$ . A/B and B/A denote the ratios of placebo (A) to placebo + treatment (B), and placebo + treatment (B) to placebo (A), respectively. The relative fold-difference represents the ratio of the A/B (or B/A) value in the obesity model to that in the total model (O/T).

Specifically, the baseline hazard was 0.0000667 h^−1^ for the population with obesity and 0.0000558 h^−1^ for the total group (O/T ratio: 1.20). Similarly, the non-drug effect parameters remained largely consistent at 0.0018 h^−1^ and 0.0017 h^−1^, respectively (O/T ratio: 1.06), indicating minimal baseline disparities between the two groups.

In contrast, the placebo plus treatment cohort (Group B) exhibited markedly higher parameter values in the population with obesity than in the pooled total population. The O/T ratio for the baseline hazard and the O/T ratio for the non-drug effect increased to 2.18 and 1.78, respectively, reflecting a widening gap between groups upon drug administration.

Notably, within the population with obesity, when comparing the placebo and treatment groups, the baseline hazard increased 2.29-fold based on the B/A ratio, whereas the non-drug effect decayed approximately 11.5-fold faster based on the B/A ratio, indicating a substantial treatment-related parameter change.

The relative fold-differences characterizing treatment-related responses between groups were 1.823 for baseline hazard and 1.679 for the non-drug effect, based on the B/A ratio. These findings indicate that, compared with the pooled total population, the population with obesity showed a greater treatment-associated increase in baseline hazard, accompanied by a more pronounced reduction in the persistence of the non-drug effect over time.

### 3.3 VPC

The predictive performance of the nausea or vomiting model was evaluated using VPCs. Two metrics were assessed: cumulative occurrence probability of nausea/vomiting and event-free probability. Figure 2 presents the cumulative occurrence probability of nausea/vomiting and the event-free probability for both the obesity model and the total model. The observed Kaplan–Meier (KM) estimates were compared against the 95% PI derived from 100 simulations.

**FIGURE 2:**
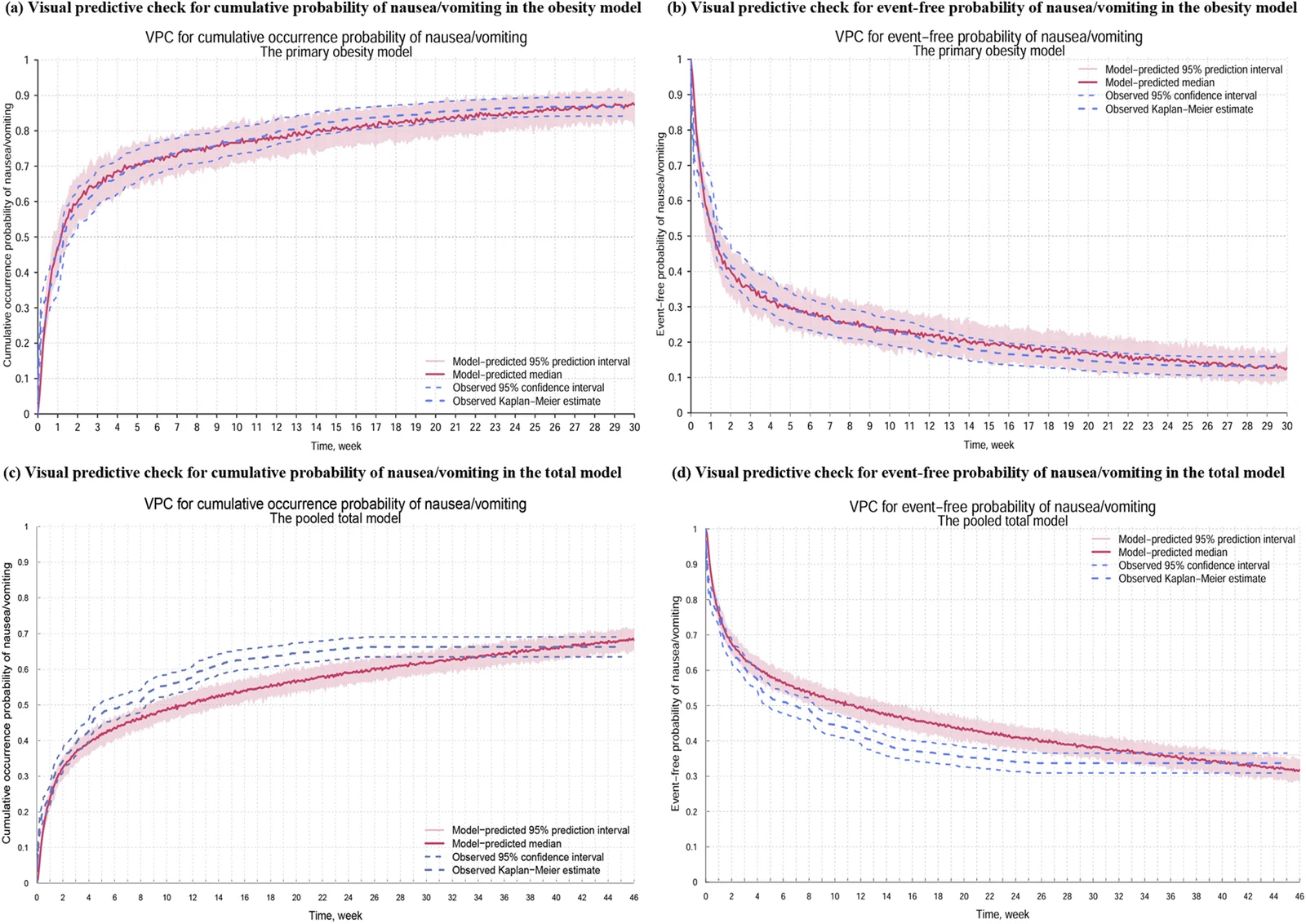
Visual predictive checks for cumulative nausea/vomiting occurrence and cumulative event-free probability in the primary obesity and pooled total models. The upper-left **(a)** shows the visual predictive check for the cumulative occurrence probability of nausea/vomiting in the primary obesity model, and the upper-right **(b)** shows the visual predictive check for the cumulative event-free probability of nausea/vomiting in the primary obesity model. The lower-left **(c)** shows the visual predictive check for the cumulative occurrence probability of nausea/vomiting in the pooled total model, and the lower-right **(d)** shows the visual predictive check for the cumulative event-free probability of nausea/vomiting in the pooled total model. The solid pink line represents the model-predicted median, and the shaded pink area represents the 95% prediction interval from model simulations. The dashed blue line represents the observed Kaplan–Meier curve, and the blue dashed confidence bands represent the 95% confidence interval of the observed data. Time is shown in weeks.

#### 3.3.1 Obesity model

VPC results for the patient population with obesity (Figure 2a) demonstrated a rapid initial surge in the cumulative probability of nausea or vomiting during the first 7 days post-dose. Subsequently, a marked attenuation in the rate of increase was observed starting approximately weeks 3–4, with the cumulative incidence profile reaching a near-plateau phase by weeks 10–11. The observed Kaplan–Meier estimates were generally well captured within the model-predicted 95% prediction intervals. Based on the simulated VPC profiles generated using the estimated model parameters, the time to reach ${TC}_{50}$ and ${TC}_{90}$ were graphically determined as 14.54 days and 82.11 days (11.73 weeks), respectively, with an associated $C_{\max}$ of 641.18 ng/mL.

#### 3.3.2 Total model

For the pooled total model, the model-predicted profiles (Figure 2c) indicated a sharp initial rise in the cumulative probability of AEs during the first 1–2 weeks of the treatment phase, followed by a gradual deceleration in the rate of increase from approximately week 4–5 onwards. After weeks 10–11, the incremental growth rate further diminished, with the cumulative incidence curve reaching a near-plateau.

In comparison with model predictions, the observed data showed a steeper initial increase in cumulative probability during weeks 1–2, with the inflection point delayed to approximately weeks 4–5. This finding indicates that a higher level of drug exposure may be required to trigger the onset of tolerance development compared with the model’s assumptions, resulting in a slower observed rate of tolerance. Consequently, during the period of tolerance development (approximately weeks 4–32), the model tended to slightly underpredict the cumulative probability of AEs, although discrepancies generally remained within approximately 10 percentage points.

### 3.4 Simulation

Monte Carlo simulations were conducted to evaluate the impact of efpeglenatide exposure on the incidence of nausea or vomiting across nine dosing regimens (Table 3) incorporating titration phases. The simulation results for the nine scenarios from each model are summarized in Figure 3 for the obesity model. Scenario-specific prediction curves showing the median and 95% prediction intervals are presented in Supplementary Material S11 for the obesity model.

**FIGURE 3:**
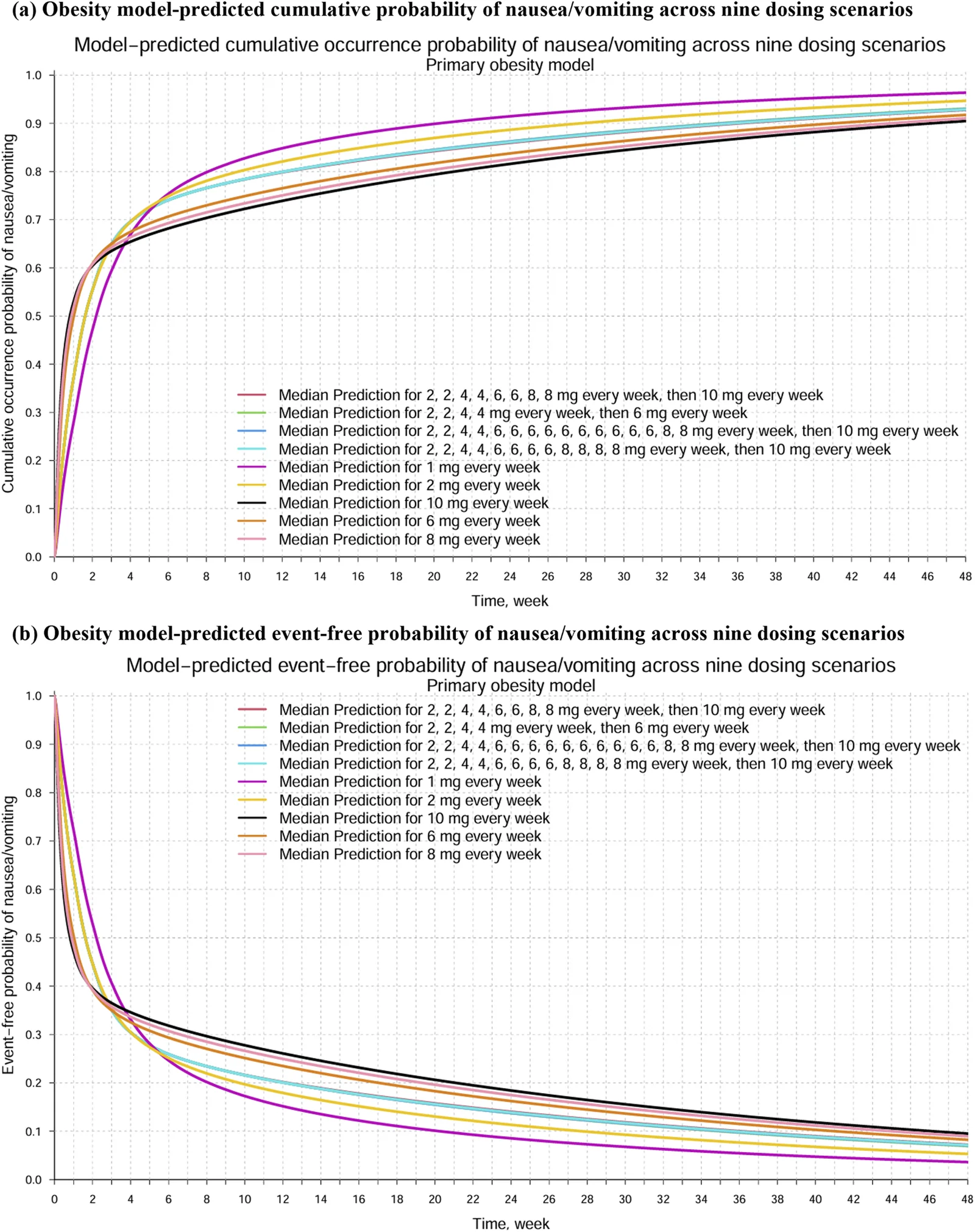
Primary obesity model–predicted cumulative nausea/vomiting occurrence and event-free probability across nine dosing scenarios. The upper **(a)** presents the primary obesity model–predicted cumulative occurrence probability of nausea/vomiting across nine dosing scenarios, whereas the lower **(b)** presents the corresponding cumulative event-free probability. Each line represents the median model-predicted profile for one dosing scenario.

#### 3.4.1 Obesity model

Monte Carlo simulations using the population with obesity model demonstrated that the cumulative incidence curves for all scenarios (Cases 1–4) were largely superimposed, with nearly identical median cumulative probabilities and 95% PI (Figure 3, Supplementary Material S11).

In the additional simulations, the low-dose once-weekly 2 mg regimen showed an occurrence pattern similar to that observed in Cases 1–4, whereas the low-dose once-weekly 1 mg regimen showed a higher cumulative probability of nausea or vomiting after 4 weeks despite lower drug exposure. In the high-dose once-weekly 6, 8, and 10 mg regimens, the cumulative probability of nausea or vomiting increased sharply during the early treatment period; however, as tolerance developed earlier in response to rapid cumulative exposure accumulation, the attenuation of the curve slope occurred earlier than in Cases 1–4, and the subsequent cumulative probability of nausea or vomiting was predicted to be lower than that in Cases 1–4.

The time points at which ${TC}_{50}$ and ${TC}_{90}$ are reached, as well as the steady-state concentrations and their corresponding attainment times across the nine simulation scenarios, are summarized in Table 7. Across all scenarios, the cumulative incidence curves increased sharply within the first 7 days following initial dosing.

**TABLE 7:** Estimated tolerance development times and steady-state exposure metrics across dosing scenarios based on the primary obesity model.

Obesity model				
Scenario	${TC}_{50}$	${TC}_{90}$	$C_{\max,ss}$ (ng/mL)	$T_{\max,ss}$ (weeks)
VPC	14.54 days	11.73 weeks (82.11 days)	Not reached (max; 641.18 ng/mL)	
Case 1	3.19 weeks (22.30 days)	9.30 weeks	1,619.00	14.30
Case 2	3.19 weeks (22.30 days)	10.00 weeks	971.40	13.32
Case 3	3.19 weeks (22.30 days)	10.01 weeks	1,619.00	25.33
Case 4	3.19 weeks (22.30 days)	9.80 weeks	1,619.00	19.32
1 mg weekly	5.89 weeks	40.20 weeks	161.90	7.29
2 mg weekly	3.58 weeks (25.08 days)	20.98 weeks	323.80	9.32
10 mg weekly	9 days (216 h)	5.44 weeks	1,619.00	9.31
6 mg weekly	12.29 days (295 h)	8.05 weeks	971.40	9.31
8 mg weekly	10.25 days (246 h)	6.43 weeks	1,295.20	9.30

${TC}_{50}$ and ${TC}_{90}$ represent the time required to reach 50% and 90% of the maximum adaptive response (tolerance), respectively. $C_{\max,ss}$ , maximum concentration at steady-state; $T_{\max,ss}$ , time to reach $C_{\max,ss}$ ; The VPC row represents the dosing regimens derived from the clinical trial data used for model development. Because multiple dosing regimens were included in the VPC dataset, steady state was not reached; therefore, the maximum concentration observed in the VPC-based concentration profile was reported instead. *TC*_50_ and *TC*_90_ were determined from simulated cumulative AUC profiles, with time on the x-axis and cumulative AUC on the y-axis, as illustrated in Supplementary Material S13. The corresponding time points were calculated as the times at which each cumulative AUC trajectory crossed the model-estimated *TC*_50_ and *TC*_90_ threshold.

The graphically derived ${TC}_{50}$ and ${TC}_{90}$ values were consistent with the inflection points and plateau phases observed in the simulation profiles (Table 7). In the low-dose once-weekly 2 mg regimen and Cases 1–4, ${TC}_{50}$ was reached at approximately 3–4 weeks, whereas the once-weekly 1 mg regimen reached ${TC}_{50}$ at approximately 6 weeks, corresponding to the time point at which the slope of the cumulative occurrence curve (Figure 3a) began to decrease (Table 7). In the high-dose once-weekly 6, 8, and 10 mg regimens, ${TC}_{50}$ was reached between approximately week 1 and week 2, representing approximately 1.5 weeks. In Figure 3a, the slope of the curve was also observed to begin decreasing shortly before week 2 for these three regimens.

${TC}_{90}$ was reached at approximately 9–10 weeks in Cases 1–4, consistent with the near-plateau phase observed around week 11 in Figure 3a and Supplementary Material S11. In contrast, the once-weekly 1 mg and 2 mg regimens reached ${TC}_{90}$ later, at approximately week 40 and week 20, respectively, reflecting a delayed attainment of maximal tolerance. Accordingly, after approximately week 4 (the ${TC}_{50}$ time point in Cases 1–4), the cumulative probability of nausea or vomiting in the low-dose once-weekly 1 mg and 2 mg regimens was slightly higher than that in Cases 1–4, although the difference in cumulative probability was within approximately 0.1 (Figure 3a; Table 7; Supplementary Material S11). In the high-dose once-weekly 6, 8, and 10 mg regimens, the cumulative probability of nausea or vomiting was predicted to increase sharply during the early treatment period. Thereafter, the curve slope was attenuated earlier than in Cases 1–4, and in the long term, these regimens tended to show a lower cumulative probability of nausea or vomiting than Cases 1–4.

#### 3.4.2 Total model

In the Monte Carlo simulations based on the pooled total model, the cumulative occurrence curves of nausea/vomiting for the 2 mg once-weekly regimen were generally overlapping with those for Cases 1–4, whereas the low-dose 1 mg once-weekly regimen showed a relatively lower incidence of nausea/vomiting (Supplementary Material S12). In contrast, the high-dose 6, 8, and 10 mg once-weekly regimens showed higher incidences of nausea/vomiting than Cases 1–4. These cumulative occurrence probability profiles of nausea/vomiting from the pooled total model differed from those obtained using the primary obesity model. Specifically, tolerance development was slowest in the low-dose regimens, intermediate in Cases 1–4 incorporating titration, and fastest in the high-dose regimens. Correspondingly, the cumulative incidence of nausea/vomiting was highest in the high-dose regimens, followed by Cases 1–4 incorporating titration and the low-dose regimens.

These results suggest that, in the pooled total model, it was difficult to explain the overall occurrence pattern of nausea/vomiting solely by the timing of tolerance development. This may be attributable to the lower overall incidence of nausea/vomiting in the total model compared with the primary obesity model, such that the relatively low absolute adverse-event burden, reflected in the more gradual cumulative occurrence pattern, made the attenuation associated with tolerance less apparent.

In addition, the pooled total model was developed using heterogeneous data that included both patients with type 2 diabetes mellitus and non-diabetic patients with obesity. Differences in drug exposure levels, disease status, and obesity status across patient groups may therefore have been reflected in a complex manner. Accordingly, in the pooled total model, there were limitations in directly linking tolerance development to the cumulative occurrence pattern of nausea/vomiting, and these results were interpreted only as exploratory findings rather than as primary evidence for dose-regimen interpretation.

## 4 Discussion

### 4.1 Principal findings

The present study characterized the longitudinal dynamics of nausea or vomiting associated with efpeglenatide using RTTE modeling and suggested that tolerance development is the likely primary mechanism underlying the decline in AE incidence after the early treatment phase. The non-diabetic obesity model developed in this analysis adequately reproduced the AE patterns and demonstrated overall good predictive performance. Approximately 50% of tolerance developed around weeks 3–4, and this early tolerance formation appeared to determine the long-term AE profile, highlighting the clinical importance of the initial dose-escalation (titration) period. These findings suggest that early cumulative drug exposure plays a key role in inducing physiological adaptation (e.g., receptor downregulation, central nervous system adaptation, *etc.*), which subsequently stabilizes the AE profile despite continued drug exposure.

### 4.2 Advantages of the RTTE modeling approach

Repeated AE analyses have traditionally relied on time-to-first-event models or Markov chain approaches (Zingmark et al., 2005). However, time-to-first-event models are limited in their ability to capture the full dynamics of recurrent events, including temporal changes in event frequency and the development of tolerance over time. Markov models also present several limitations. First, the discretization of time assumes state transitions at fixed intervals, which may fail to capture continuous event processes and can lead to information loss. Second, the memoryless property inherent to Markov models may not adequately represent biological processes, such as tolerance development, which depend on cumulative drug exposure. Additionally, as the number of states increases, model complexity and parameter uncertainty can increase relatively, making it difficult to clearly interpret the effects of drug exposure or tolerance on transition probabilities.

In contrast, the RTTE modeling framework applied in this study may allow continuous-time representation of event occurrence without information loss and can help maintain model stability even in the presence of multiple recurrent events (Karlsson et al., 2011; Ooi et al., 2025). In particular, this framework allowed direct incorporation of cumulative drug exposure as a time-varying covariate (Karlsson et al., 2019), thereby jointly accounting for the dosing regimen, individual pharmacokinetic (PK) variability, the time course of cumulative exposure accumulation, and differences in the time to reach the tolerance threshold and quantitatively evaluating their impact on the adverse-event occurrence pattern. Although the present model was not a mechanistic model that explicitly describes biological mechanisms, such as gastric emptying delay, gastrointestinal motility, or changes in central nervous system signaling pathways, as in physiologically based pharmacokinetic (PBPK) models, it can be interpreted as a biologically plausible exposure-based empirical modeling approach for explaining the onset and attenuation patterns of nausea or vomiting.

In addition, a notable key methodological feature of this study is that the proposed RTTE model addressed limitations of conventional RTTE approaches by explicitly accounting for inter-event dependency (Seo, 2024; Seo and Lim, 2026). In this analysis, these were handled through the concepts of conditional probability. For exact-time events, the conditional hazard was defined over each inter-event interval based on the mean rate of change to reflect prior event history. For interval-censored events, the conditional survival incorporated cumulative event information, and the cumulative hazard was formulated using combinatorial principles capable of accounting for multiple events within an interval, thereby potentially improving the accuracy of event-free probability estimation. A detailed description of the model structure and mathematical formulation is provided in Supplementary Material S1 (Seo, 2024; Seo and Lim, 2026), and the overall modeling workflow is described in the methodology section.

### 4.3 Methodological features of the proposed model

The key methodological distinction of the present model lies in the use of an exponential hazard model for nausea or vomiting, and in the explicit separation of early event patterns into a non-drug effect component and a tolerance component driven by cumulative AUC. This modeling framework was intended to allow the time-dependent background risk and exposure-dependent drug effects to be evaluated separately.

#### 4.3.1 Rationale for selecting the exponential hazard model

In models such as the Weibull or log-logistic hazard models, where the hazard decreases over time or shows an increase followed by a decrease, the model parameters simultaneously reflect both non-drug and drug effects, making it difficult to interpret these effects independently. In contrast, GI AEs, such as nausea or vomiting, often occur irregularly without a clear time-dependent pattern. Therefore, assuming a constant baseline hazard over time may represent a reasonable approximation, and the exponential hazard model was selected as the final model.

#### 4.3.2 Biological rationale for the early time-varying non-drug effect

From a pharmacological perspective, GI AEs associated with GLP-1 receptor agonists typically increase during the early treatment period and then gradually decrease (Drucker, 2018; Holst et al., 2024). This pattern has been attributed, at least in part, to temporary physiological imbalance and central nervous system responses during the early treatment phase (Drucker, 2018; Nauck et al., 2011). To account for this, the model incorporated a time-decaying non-drug effect to describe early event patterns (Niebecker et al., 2013), while drug effects were modeled separately.

#### 4.3.3 Rationale for the cumulative AUC-driven tolerance structure

Unlike previous models that incorporated time-delay or effect-compartment structures (Tang et al., 2024; Niebecker et al., 2013), the present model implemented tolerance as a function of cumulative AUC rather than time.

From a physiological perspective, gastrointestinal tolerability with GLP-1 receptor agonists is known to be associated with adaptation to delayed gastric emptying and central pathways involved in satiety and nausea signaling. Therefore, incorporating cumulative AUC as a function that captures both time and exposure, rather than time alone, was considered appropriate (Nauck et al., 2011; Jalleh et al., 2025b).

Cumulative AUC generally increases as treatment duration increases under repeated dosing conditions and may therefore be correlated with treatment time. However, the key consideration in this study was not the simple correlation between cumulative AUC and time, but rather the slope of the cumulative AUC–time relationship, that is, the rate at which cumulative exposure increases over time, which may vary according to the dosing regimen and individual PK characteristics. Even when the same treatment duration has elapsed, cumulative exposure may be formed differently under fixed high-dose, low-dose maintenance, or stepwise titration regimens, and the time required to reach a given tolerance threshold may consequently differ.

In fact, across the nine dosing regimens evaluated in the simulation, the cumulative AUC–time profiles differed substantially, and the time points at which ${TC}_{50}$ and ${TC}_{90}$ were reached also varied across regimens. These results are presented in Table 7 and Supplementary Material S13. This indicates that the time required to reach the tolerance threshold is not uniform across dosing simulation regimens. In contrast, in a time-driven tolerance model, tolerance is determined solely as a function of time; therefore, once the same amount of time has elapsed, similar tolerance development would be assumed regardless of dose level or titration strategy.

Additionally, based on the final obesity model, which incorporated cumulative AUC–driven tolerance, five alternative models were further compared (Supplementary Material S14a): time-driven tolerance models and models without a tolerance component. The time-driven tolerance models were constructed by applying time-dependent tolerance to three alternative parameters: the slope parameter of the log-linear structure, the $E_{\max}$ parameter of the $E_{\max}$ structure, and the ${EC}_{50}$ parameter of the $E_{\max}$ structure. As shown in Supplementary Materials S14 and S15, the model incorporating time-dependent tolerance on the ${EC}_{50}$ parameter of the $E_{\max}$ structure (Supplementary Material S15d) did not adequately reproduce the observed cumulative probability profile.

The remaining two models (Supplementary Material S15b-c) were able to describe the observed cumulative probability pattern after approximately 2 weeks; however, compared with the final cumulative AUC–driven model, they did not consistently reproduce the rapid event accumulation during the early treatment period, particularly during weeks 1–2, or the subsequent attenuation or plateau phase. In addition, the ${TC}_{50}$ estimated from the time-driven tolerance models (Supplementary Material S14b) was very short, approximately 0.1–1 week, suggesting that 50% of the tolerance effect would be formed at a very early time point after treatment initiation; however, this timing did not align with the inflection point observed in the VPC profiles, unlike the final cumulative AUC-driven tolerance model.

This limitation was more pronounced in the no-tolerance model (Supplementary Material S15e,f). Even when an $E_{\max}$ or log-linear concentration–effect structure was applied, the absence of a tolerance component prevented adequate characterization of the rapid event accumulation during early treatment and the subsequent attenuation or plateauing of the cumulative probability profile.

Taken together, these findings suggest that the cumulative AUC–driven tolerance model (Supplementary Material S15a) was more appropriate than the time-driven or no-tolerance structures for describing differences in the early nausea/vomiting burden across titration regimens and the longitudinal pattern in which event accumulation is attenuated after tolerance develops following cumulative exposure.

### 4.4 Rationale for developing separate obesity and total models

Several models were applied to the obesity group, among which the log-linear exposure–hazard model most appropriately reproduced the observed longitudinal AE profile, particularly the rapid early increase in risk followed by the subsequent attenuation or plateau phase (Müller et al., 2019). In the representative comparison, the $E_{\max}$ model yielded a slightly lower OFV than the log-linear model; however, the log-linear model showed superior VPC agreement compared with the *E*_*max*_ model. Therefore, the log-linear model was considered more appropriate for predicting the AE pattern and was selected as the final model.

In contrast, because T2DM status, obesity status, and BMI were included as covariates affecting drug exposure in the previously developed PK model, the total model was developed to further evaluate the relationship between differences in PK exposure attributable to patient-group characteristics and nausea/vomiting risk in the pooled dataset. Thus, the total model was interpreted not as a model demonstrating an independent and definitive concentration–hazard relationship in a pure T2DM population, but as an exploratory model intended to complement the obesity model–centered interpretation.

In the pooled total dataset, differences in predictive performance between the log-linear and *E_max_* models were limited, with the log-linear model showing a slightly lower OFV than the $E_{\max}$ model. However, although the slope and intercept parameters of the log-linear model can be used to describe the concentration–hazard relationship, they do not provide a pharmacological threshold concept directly linked to drug concentration, such as ${EC}_{50}$ . Because the log-linear model had already been applied to the obesity group to adequately characterize the AE pattern, the $E_{\max}$ structure was retained in the total model to interpret adverse-event risk associated with exposure differences attributable to patient-group characteristics on the basis of ${EC}_{50}$ (Gabrielsson and Weiner, 2016). This selection does not imply that the $E_{\max}$ model was globally superior to the log-linear model; rather, it was interpreted as a practical parameterization intended to provide a complementary pharmacological interpretation of concentration-related AE risk in the overall pooled dataset.

The comparative results of the major candidate structural models are presented in Supplementary Materials S16 and S17. These tables summarize the OFV, VPC performance, ${TC}_{50}$ estimates, model interpretation, and final model selection for the log-linear and *E_max_* candidate models evaluated in the obesity and pooled total datasets. Notably, ${TC}_{50}$ was consistently estimated across the candidate models in both the obesity and total datasets, supporting that the AUC-driven tolerance component identified in this analysis can be described in a relatively stable manner and is not dependent on a specific form of the concentration–hazard function.

### 4.5 Comparison of baseline hazard and non-drug effects across placebo, treatment, obesity, and pooled total groups

The parameter comparisons in Table 6 were interpreted as exploratory comparisons between the obesity model and the pooled total model, rather than as direct comparisons between a pure T2DM group and a non-diabetic obesity group. In addition, although the placebo-plus-treatment model included both placebo and active-treatment data, it was interpreted as representing the combined treatment condition because active-treatment data accounted for the majority of the dataset. Therefore, the results in Table 6 should be interpreted as an exploratory evaluation of baseline hazard and non-drug effect patterns under the placebo-only and combined treatment conditions, rather than independent baseline comparisons for specific patient groups.

#### 4.5.1 Comparison between the obesity and pooled total populations under placebo and treatment conditions (Table 6; ratio O/T)

When the obesity model and pooled total model were compared under the placebo-only condition in Table 6, the parameters related to baseline hazard and the non-drug effect did not show substantial differences. This suggests that, in the absence of drug exposure, the baseline physiological or background non-drug components may be largely comparable between the two groups. However, the slightly higher baseline hazard estimated in the obesity model suggests that factors such as chronic low-grade inflammation and altered gastrointestinal motility associated with obesity may have partly contributed to background gastrointestinal susceptibility (Khanna et al., 2022; Cani et al., 2008; Miron and Dumitrascu, 2019; Emerenziani et al., 2020).

Under the placebo-plus-treatment condition, the baseline hazard was estimated to be higher in the obesity model than in the pooled total model. This suggests that patients with obesity may have a more pronounced early nausea/vomiting burden after treatment initiation. This difference may reflect not only increased drug exposure, but also expectation associated with newly initiated injectable therapy or nocebo-related responses, as well as obesity-related gastrointestinal susceptibility (Faasse and Petrie, 2013; Petrie and Rief, 2019; Colloca and Miller, 2011). In contrast, in the pooled total population including patients with T2DM, treatment-related symptom-reporting patterns may differ from those in the obesity group owing to long-term medication use and disease-management experience (Faasse and Petrie, 2013; Petrie and Rief, 2019). Furthermore, under treatment conditions, the larger estimated non-drug-effect decay rate constant in the obesity group than in the pooled total group may be interpreted as a consequence of the model structure, in which a larger decay constant was estimated, reflecting more of the event risk being captured by the relatively higher baseline hazard. Additionally, this may be clinically attributable to the possibility that, in the pooled total population including patients with T2DM, the time course of symptoms or responses may differ from that in the obesity group owing to diabetes-related alterations in gastrointestinal function (Holst et al., 2024; Nauck et al., 2011).

#### 4.5.2 Comparison between placebo and treatment conditions within each population (Table 6; ratio A/B or B/A)

In both the obesity and pooled total populations, the estimated parameters under the combined treatment condition were generally higher than those under the placebo condition. In addition, the time to 97% loss of the non-drug effect shortened from approximately 2.7–2.8 months under placebo to approximately 6.94–12.34 days under treatment. This suggests that, under the combined treatment condition, non-pharmacological components related to nausea/vomiting, including expectation effects or nocebo-related responses, may be most prominent during the first 1–2 weeks after treatment initiation and subsequently attenuate. This may be attributable to the inclusion of active-treatment data in the treatment conditions, whereby a substantial proportion of the early change in adverse-event occurrence was captured by drug-effect parameters, such as slope, $E_{\max}$ , or ${EC}_{50}$ . Consequently, the remaining residual event risk may have been estimated as a larger non-drug-effect decay rate constant that attenuated rapidly over time.

#### 4.5.3 Integrated interpretation of placebo–treatment and obesity–total comparisons (Table 6; relative fold difference, O/T)

Taken together, the increases in baseline hazard and non-drug-effect decay rate constant from the placebo condition (A) to the combined treatment condition (B) may be more pronounced in the obesity group than in the pooled total group. These findings suggest that, compared with the pooled total population, patients with obesity may show a greater early increase in baseline AE risk after treatment initiation and a larger decrease in the duration of non-drug effects (Jalleh et al., 2025a).

#### 4.5.4 Overall interpretation and limitation

Overall, in both the placebo model without drug exposure and the treatment model incorporating drug exposure, the baseline hazard in the obesity group was estimated to be higher than that in the pooled total population. In particular, in the combined treatment model, the baseline hazard in the obesity group increased more markedly after drug administration than in the pooled total population, while the non-drug effect attenuated more rapidly. These findings suggest that patients with obesity may be more susceptible to early nausea/vomiting after drug treatment.

Because a higher baseline hazard was observed in the obesity group even in the placebo model without drug exposure, this may partly reflect baseline gastrointestinal susceptibility or exposure-independent obesity-related AE propensity in patients with obesity (Khanna et al., 2022; Cani et al., 2008; Miron and Dumitrascu, 2019; Emerenziani et al., 2020). In the combined treatment model, the higher baseline hazard in the obesity group may reflect, at least in part, greater intrinsic obesity-related sensitivity to drug-related adverse events. In addition, considering that the previous PK model showed higher drug exposure profiles in the obesity group than in the T2DM group and that this difference persisted even after accounting for administered dose (Choi et al., 2025), an exposure-mediated increase in hazard may also have contributed to the higher early risk of nausea or vomiting observed in the obesity group.

Taken together, the higher early risk in the obesity group may reflect a combination of exposure-independent baseline obesity-related susceptibility, potentially greater intrinsic obesity-related sensitivity to drug-related adverse events, and potentially exposure-mediated effects due to higher PK exposure. However, this interpretation is inferential and based on estimated model parameters. Given the current data structure, in which the pooled total model included T2DM participants and obesity status was not consistently defined within the pooled total dataset, the relative contributions of these components could not be fully distinguished.

### 4.6 Interpretation of model parameters and VPC results

In the obesity model, the drug-induced effect on the log-hazard was successfully characterized by a log-linear relationship (Niebecker et al., 2013). Although the bootstrap diagnostics of the obesity model were generally stable, the intercept parameter of the log-linear concentration–effect model showed relatively greater uncertainty than the other parameters. To evaluate the impact of this uncertainty on model-based predictions, sensitivity simulations were conducted by fixing the intercept at three values: the bootstrap median and the lower and upper bounds of the 95% confidence interval, while retaining the remaining parameters at their final estimates. The predicted cumulative probability profiles of nausea/vomiting from the sensitivity scenarios were nearly superimposable on the final model predictions, and the interpretation of the rapid early event accumulation followed by attenuation or plateauing was preserved. These findings indicate that uncertainty in the intercept estimate is unlikely to affect the main conclusions of the obesity model. The results of the sensitivity simulations are presented in Supplementary Material S9.

The log-linear model in the population with obesity theoretically allows an unbounded increase in hazard; however, the estimated slope parameter ( $\theta_{slope}$ ) was 0.627, which was below 1 (Niebecker et al., 2013; Karlsson et al., 2019). A slope value less than unity indicates a concave concentration-hazard profile, in which the marginal increase in risk diminishes as drug concentration rises (Gabrielsson and Weiner, 2016). Consequently, the model underscores a high sensitivity to hazard at lower exposure levels, whereas the rate of risk escalation tends to attenuate as concentrations approach the simulated $C_{\max}$ of 641.18 ng/mL (Choi et al., 2025). In addition, under maximum tolerance conditions, the obesity model showed an approximately 250-fold reduction in the effective concentration–effect slope, indicating a substantial decrease in sensitivity to drug exposure.

In the pooled total model, the relationship between drug exposure and adverse-event risk could be approximately described by a nonlinear $E_{\max}$ structure; however, the bootstrap confidence intervals for the estimated $E_{\max}$ and ${EC}_{50}$ parameters were relatively wide. This may reflect the complex influence of study design, observation period, prior treatment, disease status, obesity status, and individual PK-derived exposure differences. In particular, as shown in Supplementary Material S18, individual PK-derived cumulative AUC trajectories were broadly distributed across patients even within the same treatment duration, which may have limited the precise identification of the pharmacologically interpretable region of the nonlinear concentration–effect relationship around ${EC}_{50}$ in the pooled total model.

In the pooled total model, ${EC}_{50}$ was estimated to be 81.0 ng/mL, which corresponds to the concentration range that could be reached within approximately 2–3 days after once-weekly administration of 2 mg. However, because the bootstrap confidence interval for ${EC}_{50}$ was relatively wide, this value should be interpreted with caution and not as a definitive pharmacological threshold. Even when the upper bootstrap confidence bound (307.315 ng/mL) was considered, the concentration range corresponding to ${EC}_{50}$ was estimated to be attainable in Cases 1–4 after approximately the third dose, around weeks 3–4. As shown in Supplementary Material S12, this indicates that, in Cases 1–4, the corresponding concentration range could be reached prior to the marked decrease in the slope of the cumulative adverse-event occurrence curve observed around weeks 4–5. Therefore, in the pooled dataset, these findings additionally suggest that exposure-related nausea/vomiting risk, corresponding to approximately 50% of the maximal concentration effect, may develop rapidly during the early exposure period, before the cumulative adverse-event curve begins to decelerate around weeks 4–5.

In contrast, tolerance-related parameters were estimated stably regardless of the replicate inclusion criteria. This suggests that, although the precision of direct concentration–effect parameters, such as $E_{\max}$ and ${EC}_{50}$ was limited in the pooled total model, the attenuation pattern of nausea/vomiting risk observed during repeated dosing—namely, the cumulative AUC–driven tolerance component—may have been identified relatively consistently (Nauck et al., 2011).

In the obesity model, the VPC (Figures 2a,b) results indicated that the risk of nausea/vomiting began to attenuate after approximately 3–4 weeks, suggesting that AE risk progressively attenuated or approached a plateau as repeated dosing continued and cumulative exposure increased (Holst et al., 2024; Nauck et al., 2011). In this model, ${TC}_{50}$ was estimated to be 109,872 h ng/mL, corresponding to approximately 14.54 days under the dosing regimens included in the model development dataset (Müller et al., 2019). The simulated time to reach ${TC}_{90}$ was estimated to be approximately 82.11 days, which was broadly consistent with the plateauing trend observed in the VPC at approximately weeks 10–12 (Tang et al., 2024). Collectively, these findings support the contribution of the cumulative AUC-driven tolerance structure in characterizing the longitudinal pattern of nausea/vomiting in the obesity model.

In the VPC of the pooled total model (Figures 2c,d), discrepancies between the observed and predicted profiles were observed during specific time intervals, particularly between approximately week 4 and week 32 (Tang et al., 2024). These discrepancies may be partly attributable to the heterogeneous nature of the pooled dataset, which included both patients with T2DM and non-diabetic patients with obesity. In the T2DM studies, obesity status was not explicitly excluded or confirmed, and factors such as prior treatment, metformin use, dietary and lifestyle changes, and study-specific design characteristics may have influenced the observed nausea/vomiting profiles. In addition, differences in protocol-defined follow-up durations and actual observation windows across studies may have contributed to the AE patterns observed in the pooled total model and to the corresponding VPC discrepancies. Therefore, the VPC discrepancies observed in the total model were interpreted not as arising from a single specific factor, but rather as reflecting the combined influence of clinical and behavioral heterogeneity within the pooled dataset and limitations inherent to the observed data.

Accordingly, the pooled total model was not used as the primary predictive model for dosing-scenario simulations. The primary interpretation of AE burden and tolerance timing across dosing regimens was instead based on the results from the primary obesity model.

### 4.7 Individual PK-derived exposure and observed nausea/vomiting occurrence

To further evaluate how individual PK-derived exposure variability was associated with the adverse-event occurrence pattern observed in the RTTE model, the relationship between cumulative AUC calculated using individual PK parameters and the observed occurrence pattern of nausea/vomiting was additionally analyzed.

Specifically, individual PK-derived cumulative AUC was calculated in active-treatment patients, and the corresponding quartiles (Q1–Q4) were defined based on the cumulative AUC at week 4. Week 4 was selected as the representative early time point because nausea/vomiting events were concentrated during the initial treatment period, particularly within the first 4 weeks, and this time point approximately corresponded to the first inflection point at which the slope of the cumulative occurrence curve began to attenuate. Because this analysis used individual PK predictions incorporating estimated interindividual random effects, it was based on cumulative exposure estimates that reflected interindividual PK variability, rather than on simple group-mean PK profiles.

As shown in Supplementary Material S18, individual PK-derived cumulative AUC values showed a wide distribution across patients even at the same week 4 time point. This indicates that individual PK-derived cumulative exposure may differ substantially among patients despite the same treatment duration, depending on individual PK characteristics. As shown in Supplementary Material S19, comparison of the observed nausea/vomiting event burden across cumulative AUC quartiles showed that Q1 had the lowest event burden, whereas Q4 had the highest event burden. Consistent findings were also observed in the Kaplan–Meier–based cumulative probability analyses presented in Supplementary Material S20, where the week 4 cumulative occurrence probability of nausea/vomiting was higher in the highest cumulative AUC quartile (Q4) than in the lowest quartile (Q1), indicating a greater early nausea/vomiting burden. Supplementary Material S21 provided a complementary early-interval analysis of the relative timing and accumulation pattern of events observed within the first 4 weeks across the exposure quartiles, with Q4 generally showing earlier event accumulation than Q1. Although the difference between Q2 and Q3 was not substantial and Q2 appeared higher at certain time points, this was considered a natural fluctuation in the observed data. Overall, the contrast was most evident between the lowest and highest cumulative AUC quartiles.

These results suggest that differences in individual cumulative exposure may be associated with the observed early AE occurrence patterns. Accordingly, this analysis further supports that individual PK-derived exposure variability may be meaningfully linked to the nausea/vomiting occurrence patterns evaluated in the RTTE model.

### 4.8 Dosing-scenario simulations: Rationale, interpretation, and implications for titration strategy

The dosing-regimen simulations were designed to characterize the effects of titration regimen and maintenance dose on early nausea/vomiting burden and tolerance development following efpeglenatide administration.

In simulations based on the obesity model (Figure 3; Supplementary Material S11), all Cases 1–4 were predicted to reach 50% tolerance within approximately 3–4 weeks, likely reflecting the same dosing sequences applied across the four scenarios during the initial dose-escalation period (6 weeks).

In the low-dose once-weekly 2 mg fixed-dose regimen, the cumulative occurrence profile of nausea/vomiting during the first approximately 4 weeks was comparable to that observed in Cases 1–4; however, the cumulative occurrence probability of nausea/vomiting was predicted to be slightly higher thereafter. This may be explained by the fact that the 2 mg once-weekly regimen provided sufficient cumulative exposure to achieve 50% tolerance by approximately week 4, whereas the time required to achieve 90% tolerance was delayed relative to Cases 1–4. In contrast, the once-weekly 1 mg regimen showed a relatively lower cumulative occurrence probability of nausea/vomiting during the early treatment period; however, its cumulative AUC was lower than that of the other regimens, resulting in delayed tolerance development. Consequently, the cumulative occurrence probability of nausea/vomiting was predicted to be higher in the long term than that observed in Cases 1–4 and the once-weekly 2 mg regimen. This suggests that maintaining an excessively low dose over an extended period may reduce early adverse-event occurrence but may also delay the cumulative exposure required for tolerance development (Nauck et al., 2011). Conversely, the once-weekly fixed-dose regimens of 6, 8, and 10 mg were included to evaluate the early AE burden under high-exposure conditions. With fixed high-dose administration, cumulative AUC accumulated rapidly, allowing the tolerance threshold to be reached earlier; however, the predicted cumulative probability of nausea/vomiting increased markedly during the first 1–2 weeks. The interval-specific weekly increases summarized in Supplementary Material S6 also showed that the fixed high-dose regimens produced an approximately 1.3–1.4-fold greater early AE burden than Cases 1–4. These findings indicate that rapid tolerance development does not necessarily translate into a clinically favorable strategy. Thus, although high fixed-dose regimens may promote earlier tolerance development during subsequent treatment, they may also induce an excessive AE burden during the early treatment period, potentially compromising tolerability or increasing the risk of treatment discontinuation. The *TC*_50_, *TC*_90_, and steady-state exposure results for each scenario are presented in Table 7, and the dose-specific cumulative occurrence probabilities and interval-specific weekly increments are summarized in Supplementary Material S6.

Dosing-scenario simulations were also performed using the exploratory pooled total model, and the overall patterns differed somewhat from those observed with the primary obesity model (Supplementary Material S12). Similar to the obesity model, Cases 1–4 reached 50% tolerance at comparable time points, reflecting the shared initial dose-escalation phase across these scenarios. The fixed low-dose once-weekly 1 mg and 2 mg regimens generally showed lower cumulative occurrence probabilities of nausea/vomiting than Cases 1–4. In contrast, the fixed high-dose once-weekly 6, 8, and 10 mg regimens tended to show higher cumulative occurrence probabilities of nausea/vomiting than Cases 1–4, despite reaching the tolerance threshold earlier. These findings suggest that, in the pooled total model, the overall adverse-event occurrence pattern could not be fully explained by the timing of tolerance development alone. In the fixed high-dose regimens, cumulative exposure accumulated rapidly, resulting in earlier attainment of 50% tolerance; however, the cumulative occurrence probability of nausea/vomiting continued to increase, likely owing to sustained high drug exposure. Thus, irrespective of the timing of 50% tolerance development, the increase in event occurrence appeared to be predominantly driven by the magnitude and persistence of drug exposure.

These differences may reflect the complex influence of the heterogeneous pooled dataset, which included both patients with T2DM and non-diabetic patients with obesity. However, because patients with T2DM could not be clearly stratified according to obesity status in the present analysis, it was not possible to determine whether the differences between the obesity and total models were driven by differences in drug exposure related to patient-group characteristics, patient-specific susceptibility to adverse events, or differences in study design and prior treatment. Therefore, the pooled total model results should be interpreted as exploratory findings that complementarily characterize overall concentration-related AE tendencies in the heterogeneous pooled population, rather than as a basis for definitive dose-escalation decisions in a specific patient population.

Taken together, in non-diabetic patients with obesity, these simulation results suggest that controlling the rate at which cumulative exposure increases during early treatment may be more important for efpeglenatide dose-escalation strategies than simply achieving the target maintenance dose (Nauck et al., 2011). In particular, the initial 2 → 4 → 6 mg titration sequence shared across Cases 1–4 was considered to provide a rational titration framework because it may mitigate the early AE burden compared with fixed high-dose administration while achieving 50% tolerance within approximately 3–4 weeks, earlier than fixed low-dose administration (Bettge et al., 2017; Gorgojo-Martínez et al., 2023).

These findings are consistent with the clinical rationale for stepwise dose escalation commonly used for GLP-1 receptor agonists. However, because the present analysis was based on RTTE simulations focused on the occurrence pattern of nausea/vomiting, additional evaluations incorporating efficacy, weight reduction, durability of glycemic control, and discontinuation risk are warranted before recommending any specific case as the optimal regimen.

### 4.9 Comparison with other GLP-1 receptor agonists and clinical implications

GI AEs such as nausea or vomiting associated with GLP-1 receptor agonists are mechanistically linked to delayed gastric emptying (Drucker, 2018; Jalleh et al., 2025b). Activation of GLP-1 receptors reduces GI motility, prolongs gastric retention time, and may induce gastric stasis, contributing to the development or worsening of nausea or vomiting (Drucker, 2018; Jalleh et al., 2025a). This effect is typically most pronounced during the early phase of treatment, which explains the high incidence of GI AEs shortly after treatment initiation (Bettge et al., 2017; Gorgojo-Martínez et al., 2023; Wharton et al., 2022b). Over time, however, these AEs tend to decrease, likely due to physiological adaptation to the drug (Nauck et al., 2011; Wharton et al., 2022b). Continued exposure to GLP-1 receptor agonists leads to gradual adaptation of GI motility and possibly central nervous system signaling pathways, resulting in reduced sensitivity to the drug’s GI effects and a decline in the incidence of nausea or vomiting (Holst et al., 2024; Nauck et al., 2011).

Based on the results of this analysis, the time at ${TC}_{50}$ for efpeglenatide may correspond to the point at which the drug-related GI inhibitory effect, namely delayed gastric emptying, is approaching a dynamic balance with the body’s physiological adaptation (Nauck et al., 2011). Clinically, this corresponds to the time when the severity of nausea or vomiting begins to noticeably decrease (Jalleh et al., 2025b; Horowitz et al., 2017). Notably, the estimated ${TC}_{50}$ time point was consistent with the time at which the cumulative incidence curves for nausea or vomiting in the VPC began to flatten. This finding suggests that the point at which the slope of the cumulative incidence or prevalence curve begins to decrease may be interpreted as a statistical indicator corresponding to approximately 50% tolerance development.

The first period after the peak in the prevalence or occurrence profile, when the decline begins, indicates attenuation in the overall nausea/vomiting burden and may reflect a reduction in newly occurring events (Bettge et al., 2017; Lean et al., 2014). These observations suggest that physiological adaptation and tolerance to the drug have developed broadly across the patient population (Nauck et al., 2011; Gorgojo-Martínez et al., 2023). Based on these criteria, the time to approximately 50% tolerance development of nausea or vomiting for efpeglenatide was estimated and compared with those of four representative GLP-1 receptor agonists currently on the market. In the non-diabetic obesity model, application of the Cases 1–4 dosing strategy to the obesity group predicted attainment of 50% tolerance to efpeglenatide within approximately 3–4 weeks.

By integrating evidence from clinical studies, regulatory documents, cumulative incidence patterns, prevalence profiles, and attenuation patterns of delayed gastric emptying across various GLP-1 receptor agonists, the timing of approximately 50% tolerance development for nausea or vomiting was compared across agents. The evidence and inference process used to estimate the timing of approximately 50% tolerance development for each agent are summarized in Supplementary Material S22. Specifically, the estimated timing was approximately 4–8 weeks for liraglutide (Lean et al., 2014; U.S. Food and Drug Administration, 2014; Pi-Sunyer et al., 2015; Maselli et al., 2022), 16–20 weeks for semaglutide (Wharton et al., 2022a; Friedrichsen et al., 2021; U.S. Food and Drug Administration, 2021), 6–7 weeks for dulaglutide (Tang et al., 2024), and 16–20 weeks for tirzepatide (U.S. Food and Drug Administration, 2023a; U.S. Food and Drug Administration, 2023b; Urva et al., 2020; Rubino et al., 2025). Notably, when these time frames are compared with the dose-escalation (titration) periods of each drug, approximately 50% tolerance appears to develop during the late titration phase or around the time of reaching the target maintenance dose in most agents. This observation suggests that stepwise dose-escalation strategies may have been designed not only to reduce early AEs but also to allow sufficient physiological adaptation and tolerance development before reaching the full therapeutic dose.

A summary of the dosing regimens, administration methods, standard dose-escalation strategies, maintenance doses, and the estimated time to approximately 50% tolerance development for each GLP-1 receptor agonist is presented in Table 8. For efpeglenatide, based on the modeling results from the present study, a titration period of at least approximately 4 weeks appears necessary to reach the maintenance dose of 10 mg/week, and extending the titration period up to approximately 8 weeks appears to be an appropriate strategy when needed to improve tolerability.

**TABLE 8:** Comparison of GLP-1 receptor agonist titration schedules and obesity model-based efpeglenatide tolerance timing.

Drug (active ingredient)	Starting dose (mg)	Dosing frequency	Titration interval	Dose escalation steps (mg)	Total titration period (weeks)	Target maintenance dose (mg)	Time at ${TC}_{50}$ (weeks)
Liraglutide (Lean et al., 2014; U.S. Food and Drug Administration, 2014; Pi-Sunyer et al., 2015; Maselli et al., 2022)	0.6	Daily	1 week	0.6→1.2→1.8→2.4→3.0	4	3.0	4–8
Semaglutide (Wharton et al., 2022a; Friedrichsen et al., 2021; U.S. Food and Drug Administration, 2021)	0.25	Weekly	4 weeks	0.25→0.5→1.0→1.7→2.4	16	2.4	16–20
Dulaglutide (Tang et al., 2024)	0.75/1.5	Weekly	4 weeks	0.75→1.5→3.0→4.5	8–12	3.0/4.5	6–7
Tirzepatide (Jastreboff et al., 2022; U.S. Food and Drug Administration, 2023a; U.S. Food and Drug Administration, 2023b; Urva et al., 2020; Rubino et al., 2025)	2.5	Weekly	4 weeks	2.5→5.0→7.5→10.0→12.5→15.0	4–20	5/10/15	16–20
Efpeglenatide	2	Weekly	1 week	2→2→4→4 (→6→6→8→8)→10	4–8	10	3–4

Dosing regimens and titration schedules represent typical clinical dose-escalation strategies for GLP-1 receptor agonists. The total titration period varies depending on the target maintenance dose. Time at ${TC}_{50}$ for efpeglenatide is based on the modeling results of the present study. Values for other agents represent approximate estimates derived from cumulative incidence and prevalence patterns reported in published clinical trials, prescribing information, and regulatory documents. These values are intended for qualitative comparison rather than direct quantitative comparison across agents. For the other agents, ${TC}_{50}$ was defined as the time at which the occurrence or prevalence profile of nausea or vomiting began to attenuate or, where available, as the model-predicted tolerance timing; for efpeglenatide, the ${TC}_{50}$ value represents the model-predicted time to reach 50% of the maximum tolerance effect based on the primary obesity model developed in the non-diabetic obesity population. The supporting literature, clinical evidence, and inference process used to derive the estimated timing of approximately 50% tolerance development for each GLP-1 receptor agonist are summarized in Supplementary Material S22.

It should be noted that the tolerance development timelines estimated for other GLP-1 receptor agonists were indirectly derived by interpreting cumulative incidence and prevalence patterns reported in published clinical trials and regulatory documents, based on the ${TC}_{50}$ -based criterion established in the present analysis. Therefore, these estimates represent approximations rather than formally modeled values and should be interpreted with caution.

Consequently, compared with other GLP-1 receptor agonists, these results suggest that efpeglenatide may exhibit relatively rapid tolerance development to nausea or vomiting. The relatively rapid development of GI tolerance observed with efpeglenatide may have several key clinical and pharmacological implications.

Rapid tolerance development suggests the potential to reach the target therapeutic dose more rapidly. The findings may allow patients to experience therapeutic benefits, such as glycemic control and weight reduction, earlier in the course of treatment, potentially improving early clinical efficacy (Wharton et al., 2022a; Gorgojo-Martínez et al., 2023).

Additionally, rapid tolerance development may also have critical implications for treatment adherence. One of the primary reasons for treatment discontinuation with GLP-1 receptor agonists is nausea or vomiting occurring during the early treatment period (Wharton et al., 2022a; Gorgojo-Martínez et al., 2023). If physiological adaptation and tolerance develop relatively quickly, as suggested for efpeglenatide, the duration of nausea or vomiting may be shortened, potentially reducing early treatment discontinuation and improving long-term treatment adherence.

Furthermore, rapid tolerance development may provide greater flexibility in dose-adjustment strategies. The results of this study suggest that after the initial titration period of approximately 4 weeks, subsequent differences in dose-escalation speed or dosing intervals have a limited impact on GI AE incidence. This finding indicates that after the early adaptation period, more flexible dose adjustments may be possible depending on patient response and tolerability, supporting individualized or personalized dosing strategies (Lalonde et al., 2007).

Finally, agents such as efpeglenatide that exhibit rapid physiological adaptation may shorten the period requiring AE management, potentially reducing overall treatment burden. For drugs with slower tolerance development, prolonged AE management may require additional medications or increased clinical visits. As fewer healthcare resources are required, rapid tolerance development may improve both clinical and economic efficiency for patients, healthcare providers, and healthcare systems.

## 5 Limitation and future research

A key limitation of this study concerns the interpretability of the pooled total model. The model was developed using an integrated dataset that included patients with T2DM, for whom obesity status was not consistently defined, and non-diabetic patients with obesity from the HM-EXC-205 study. Accordingly, it was used as an exploratory reference model to characterize overall nausea/vomiting occurrence and concentration-related AE tendencies in the pooled dataset, while providing supportive evaluation of pharmacologically interpretable parameters such as ${EC}_{50}$ and $E_{\max}$ .

However, the dataset was characterized by substantial heterogeneity in disease status, obesity status, BMI, background therapy, study design, observation windows, dosing regimens, and individual PK-derived exposure. Such heterogeneity may have contributed to the underprediction observed in the VPC of the pooled total model during approximately weeks 4–32, as well as to the wide confidence intervals for ${EC}_{50}$ and $E_{\max}$ . Therefore, ${EC}_{50}$ in the pooled total model should not be interpreted as a definitive pharmacological threshold in a specific homogeneous patient population. Rather, it should be regarded as an exploratory descriptor; however, because the *EC*_50_ estimate remained relatively low even when considering the upper bound of its confidence interval, it provides supportive evidence that concentration-related nausea/vomiting risk may emerge relatively rapidly after treatment initiation.

In addition, the pooled total model was not intended to serve as the primary predictive model for deriving dosing regimens or titration strategies. Nevertheless, ${TC}_{50}$ was estimated similarly between the primary obesity and pooled total models, with relatively narrow confidence intervals, suggesting that the cumulative exposure–driven tolerance component was identified consistently despite heterogeneity across patient groups. However, because the absolute incidence of drug-related nausea/vomiting and exposure-related susceptibility may differ across patient populations, future studies should prospectively stratify and evaluate patients with T2DM with obesity, patients with T2DM without obesity.

Second, placebo-only and placebo-plus-treatment models were constructed to explore differences between the placebo-only and combined treatment conditions. Although the placebo-plus-treatment model included a subset of placebo data, it was interpreted as representing a combined treatment condition because active-treatment data comprised the majority of the dataset. This pooled-model strategy was used to retain the available placebo information while improving the stability of parameter estimation, given the limited number of placebo patients and events. However, this comparison was exploratory and intended to provide an indirect assessment of differences between the placebo-only and combined treatment conditions; therefore, it should not be interpreted as a direct comparison between pure placebo and pure treatment groups. Future studies with sufficient placebo and active-treatment data may allow clearer separation of treatment-related effects from non-drug effects.

Third, the comparison across GLP-1 receptor agonists presented in Table 8 was not performed within a unified modeling framework. Instead, it represents a qualitative contextual comparison that summarizes literature-reported dose-escalation schedules for selected GLP-1 receptor agonists used in obesity treatment, together with the efpeglenatide tolerance timing predicted from the primary obesity model in the present study. Therefore, Table 8 should be interpreted as a contextual reference for positioning the efpeglenatide results within the obesity-treatment setting, rather than as evidence for quantitatively comparing tolerability or adverse-event risk across agents. Future analyses using individual patient-level data from multiple GLP-1 receptor agonists within a common exposure–RTTE modeling framework may enable more direct and quantitative comparisons of nausea/vomiting risk, tolerance development, and titration strategies in patients with obesity.

## 6 Conclusion

In conclusion, this study provides a biologically plausible exposure-based framework for interpreting transient efpeglenatide-associated nausea/vomiting in relation to cumulative drug exposure and tolerance development. The VPC results from the primary obesity model, supported by the model developed using data from non-diabetic patients with obesity, indicated that gastrointestinal tolerance may develop relatively rapidly, reaching the 50% tolerance threshold within approximately 3–4 weeks.

Across the nine dosing scenarios evaluated, Cases 1–4, representing clinically applicable stepwise titration strategies, were interpreted as well-balanced approaches that could achieve approximately 50% tolerance within 3–4 weeks while controlling the early AE burden. In contrast, excessively low fixed-dose regimens may delay cumulative exposure accumulation and tolerance development, thereby increasing the long-term cumulative occurrence probability of adverse events. Conversely, high fixed-dose regimens may accelerate tolerance development but substantially increase the early AE burden, potentially increasing the risk of reduced tolerability or treatment discontinuation. Thus, stepwise titration of efpeglenatide may support clinical optimization by aligning early adverse-event mitigation with sufficient cumulative exposure for tolerance development.

Notably, this time window appears shorter than that inferred for other GLP-1 receptor agonists. Clinically, rapid tolerance development with efpeglenatide may facilitate earlier transition to therapeutic dosing, improve long-term treatment adherence, and shorten the period of highest adverse-event risk, thereby supporting more efficient use of healthcare resources.

## Data Availability

The original contributions presented in the study are included in the article and Supplementary Material. The clinical trial datasets analyzed in this study were provided by Hanmi Pharm Co., Ltd. and are not publicly available due to confidentiality and data use agreements. Further inquiries can be directed to the corresponding author.
