# Supplementary material for: Exposure-driven tolerance modeling of efpeglenatide-induced nausea or vomiting using repeated time-to-event analysis: implications for dose-escalation strategies
Source: Front Pharmacol. 2026 Sep 22;17:1846164. doi: 10.3389/fphar.2026.1846164 (PMC13639893; doi:10.3389/fphar.2026.1846164)
Supplement: Supplementary file 1 [file DataSheet1.pdf]

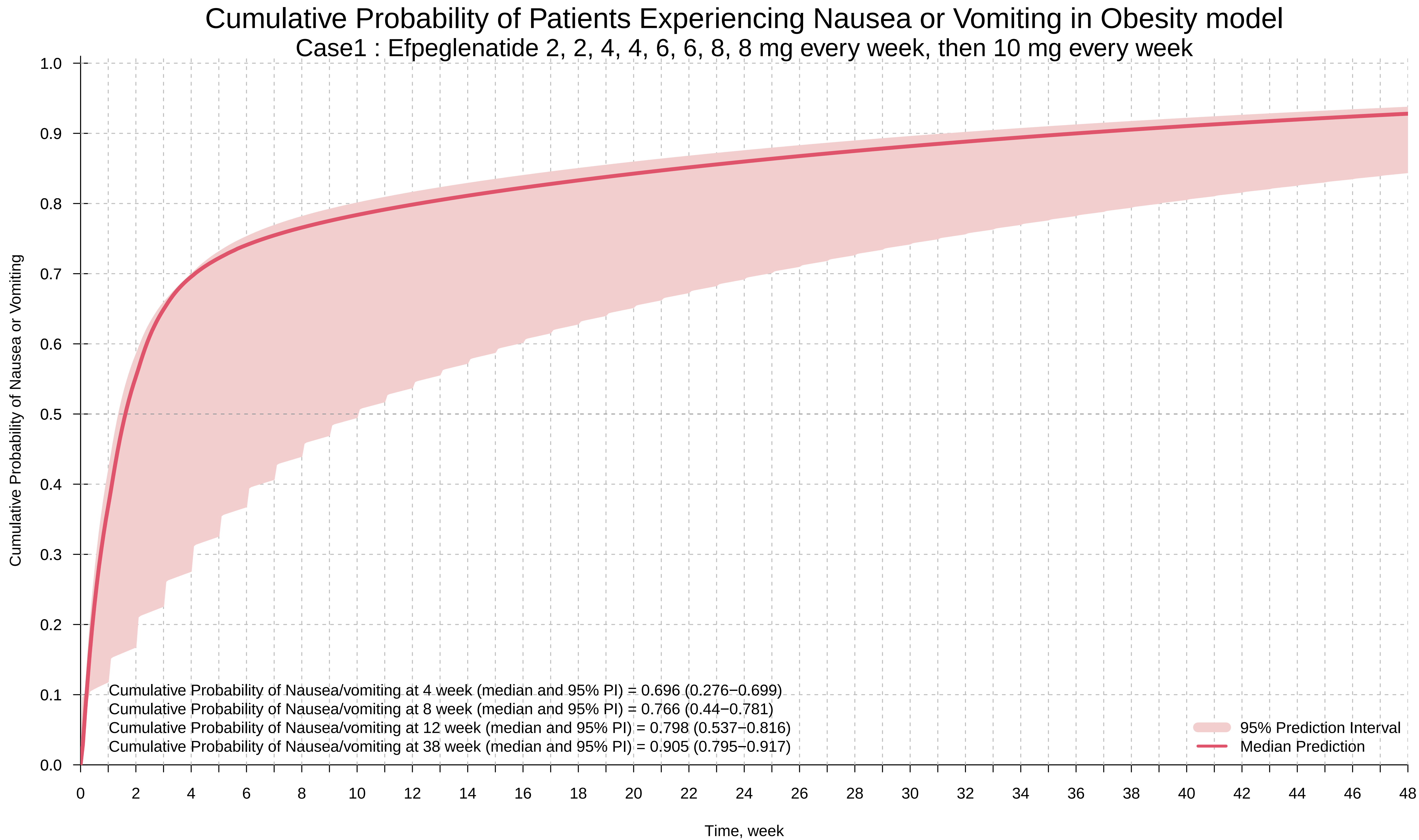

Cumulative Probability of Patients Experiencing Nausea or Vomiting in Obesity model
Case1 : Efpeglenatide 2, 2, 4, 4, 6, 6, 8, 8 mg every week, then 10 mg every week
Cumulative Probability of Nausea or Vomiting
Time, week
Cumulative Probability of Nausea/vomiting at 4 week (median and 95% PI) = 0.696 (0.276−0.699)
Cumulative Probability of Nausea/vomiting at 8 week (median and 95% PI) = 0.766 (0.44−0.781)
Cumulative Probability of Nausea/vomiting at 12 week (median and 95% PI) = 0.798 (0.537−0.816)
Cumulative Probability of Nausea/vomiting at 38 week (median and 95% PI) = 0.905 (0.795−0.917)
95% Prediction Interval
Median Prediction

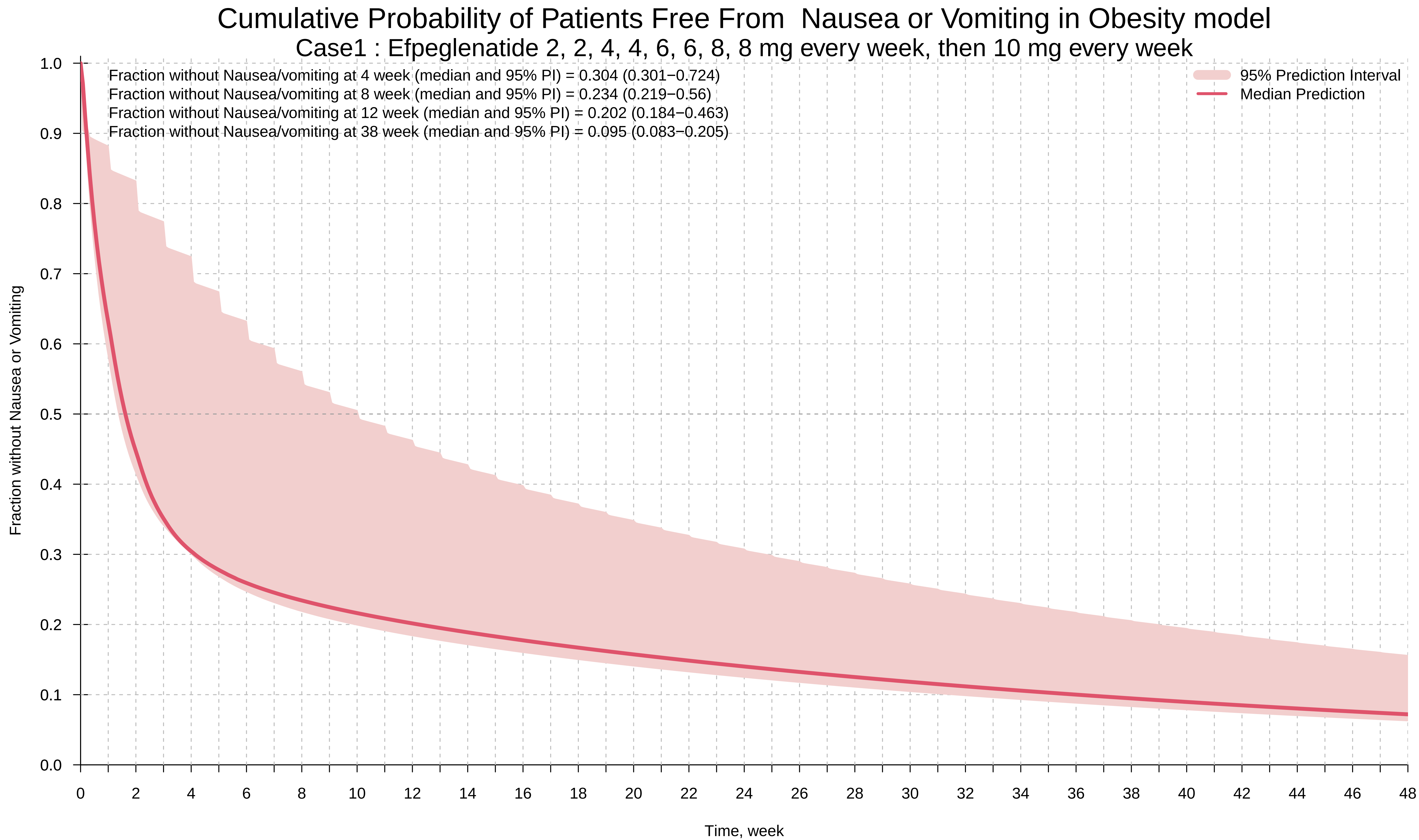
Cumulative Probability of Patients Free From Nausea or Vomiting in Obesity model
Case1 : Efpeglenatide 2, 2, 4, 4, 6, 6, 8, 8 mg every week, then 10 mg every week
Fraction without Nausea/vomiting at 4 week (median and 95% PI) = 0.304 (0.301−0.724)
Fraction without Nausea/vomiting at 8 week (median and 95% PI) = 0.234 (0.219−0.56)
Fraction without Nausea/vomiting at 12 week (median and 95% PI) = 0.202 (0.184−0.463)
Fraction without Nausea/vomiting at 38 week (median and 95% PI) = 0.095 (0.083−0.205)
95% Prediction Interval
Median Prediction
Fraction without Nausea or Vomiting
Time, week
1.0
0.9
0.8
0.7
0.6
0.5
0.4
0.3
0.2
0.1
0.0
0
2
4
6
8
10
12
14
16
18
20
22
24
26
28
30
32
34
36
38
40
42
44
46
48

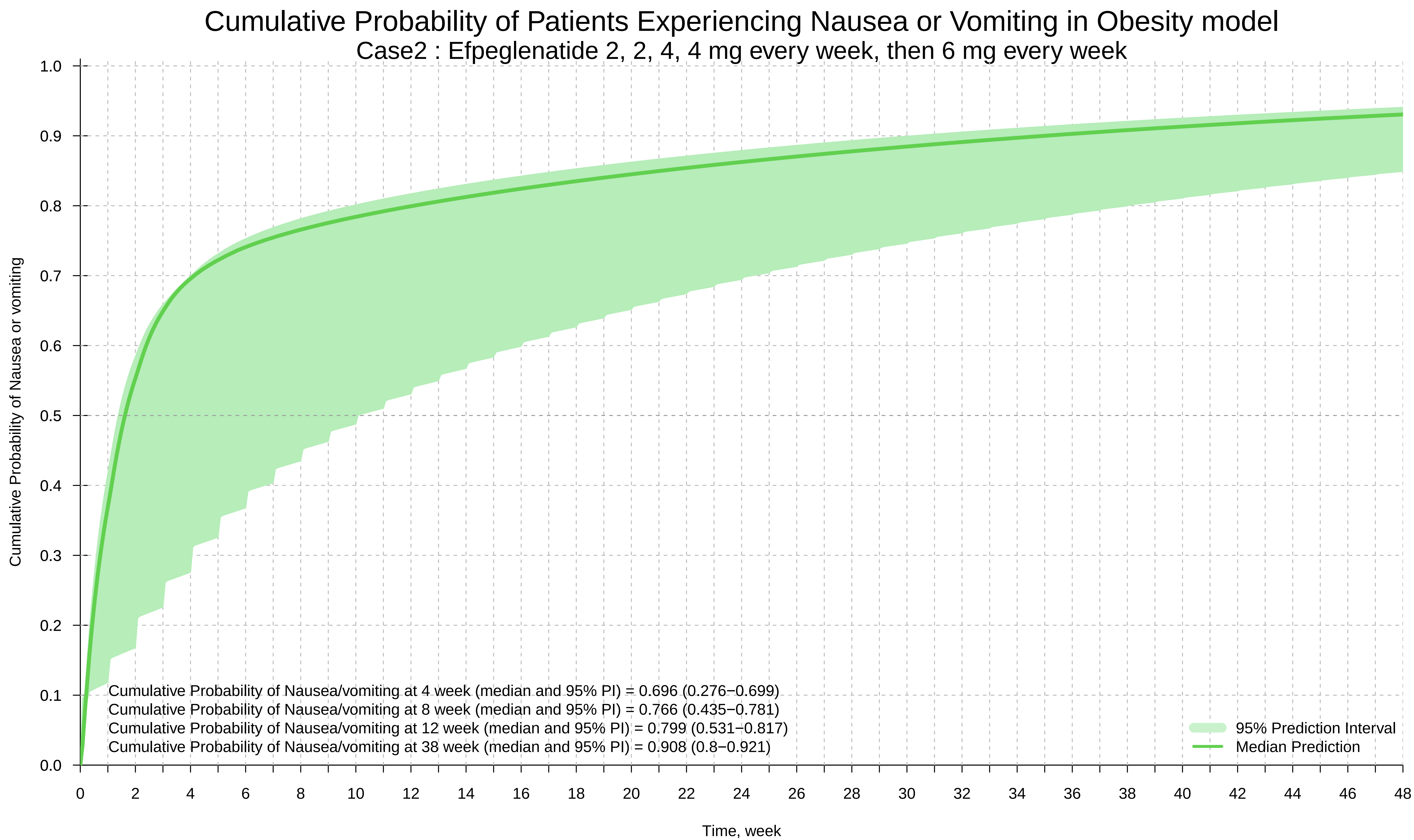
Cumulative Probability of Patients Experiencing Nausea or Vomiting in Obesity model
Case2 : Efpeglenatide 2, 2, 4, 4 mg every week, then 6 mg every week
Cumulative Probability of Nausea or vomiting
1.0
0.9
0.8
0.7
0.6
0.5
0.4
0.3
0.2
0.1
0.0
0
2
4
6
8
10
12
14
16
18
20
22
24
26
28
30
32
34
36
38
40
42
44
46
48
Time, week
Cumulative Probability of Nausea/vomiting at 4 week (median and 95% PI) = 0.696 (0.276−0.699)
Cumulative Probability of Nausea/vomiting at 8 week (median and 95% PI) = 0.766 (0.435−0.781)
Cumulative Probability of Nausea/vomiting at 12 week (median and 95% PI) = 0.799 (0.531−0.817)
Cumulative Probability of Nausea/vomiting at 38 week (median and 95% PI) = 0.908 (0.8−0.921)
95% Prediction Interval
Median Prediction

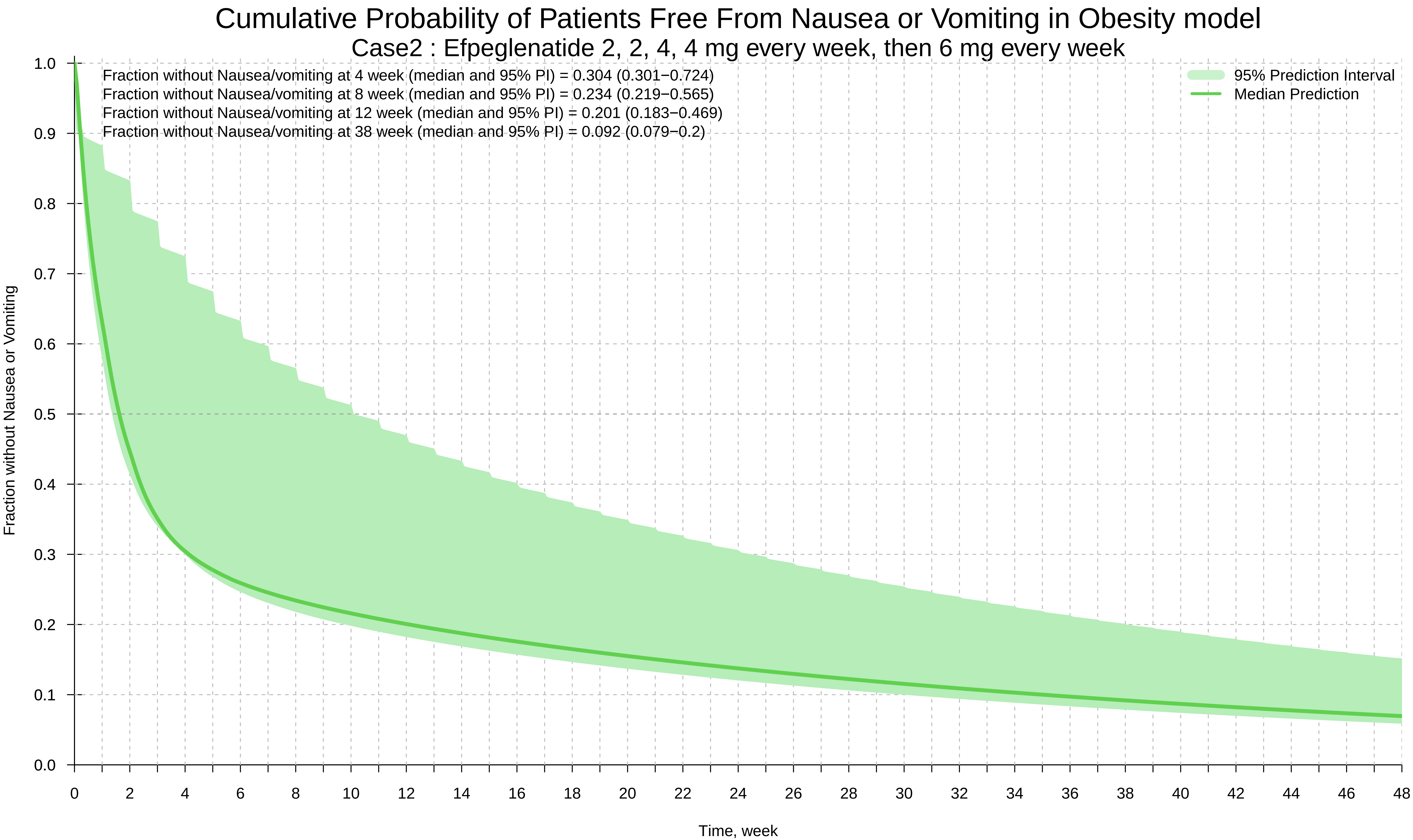
Cumulative Probability of Patients Free From Nausea or Vomiting in Obesity model
Case2 : Efpeglenatide 2, 2, 4, 4 mg every week, then 6 mg every week
Fraction without Nausea/vomiting at 4 week (median and 95% PI) = 0.304 (0.301−0.724)
Fraction without Nausea/vomiting at 8 week (median and 95% PI) = 0.234 (0.219−0.565)
Fraction without Nausea/vomiting at 12 week (median and 95% PI) = 0.201 (0.183−0.469)
Fraction without Nausea/vomiting at 38 week (median and 95% PI) = 0.092 (0.079−0.2)
95% Prediction Interval
Median Prediction
Fraction without Nausea or Vomiting
Time, week
1.0
0.9
0.8
0.7
0.6
0.5
0.4
0.3
0.2
0.1
0.0
0
2
4
6
8
10
12
14
16
18
20
22
24
26
28
30
32
34
36
38
40
42
44
46
48

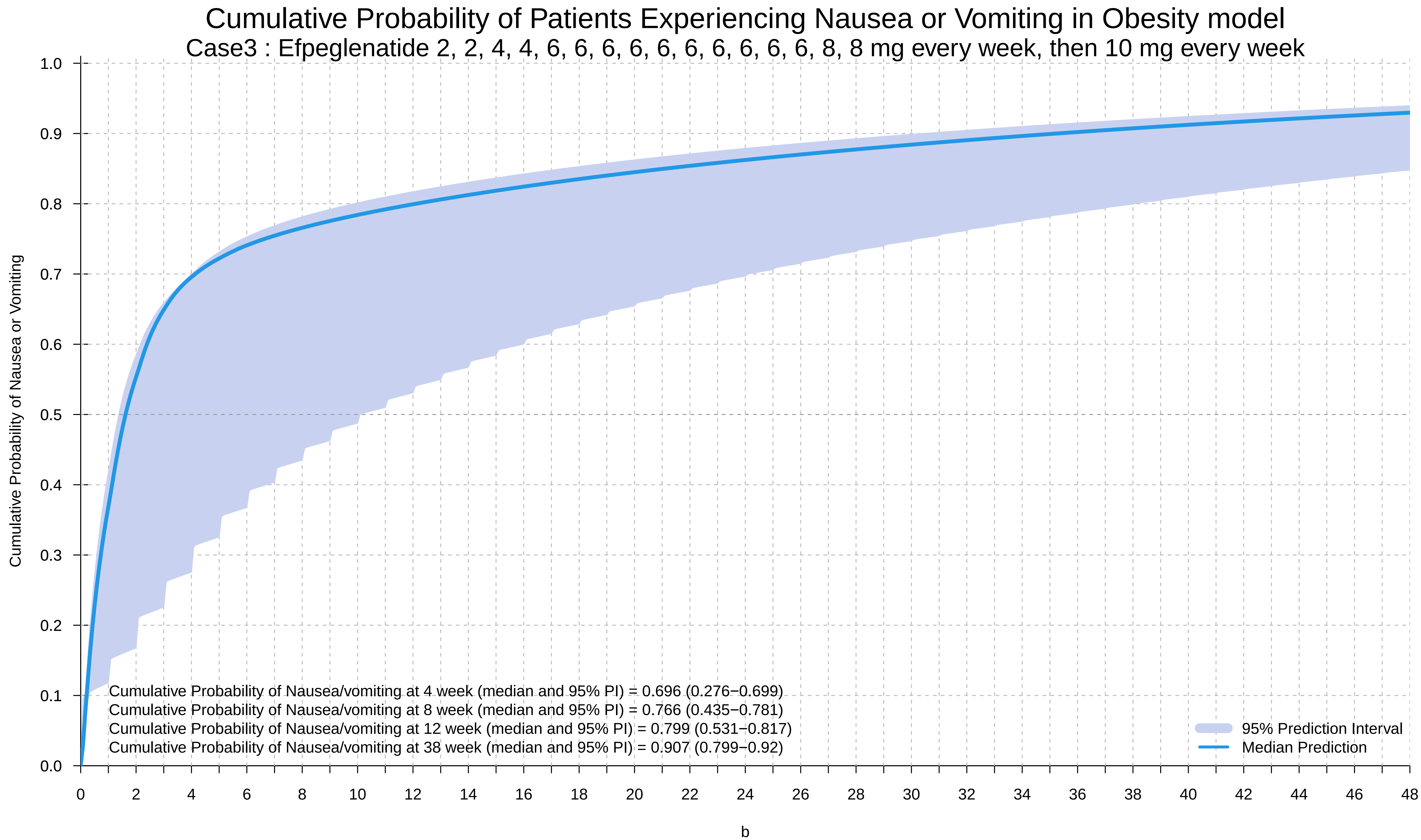

Cumulative Probability of Patients Experiencing Nausea or Vomiting in Obesity model
Case3 : Efpeglenatide 2, 2, 4, 4, 6, 6, 6, 6, 6, 6, 6, 6, 6, 6, 8, 8 mg every week, then 10 mg every week
Cumulative Probability of Nausea or Vomiting
0.0
0.1
0.2
0.3
0.4
0.5
0.6
0.7
0.8
0.9
1.0
0
2
4
6
8
10
12
14
16
18
20
22
24
26
28
30
32
34
36
38
40
42
44
46
48
b
Cumulative Probability of Nausea/vomiting at 4 week (median and 95% PI) = 0.696 (0.276−0.699)
Cumulative Probability of Nausea/vomiting at 8 week (median and 95% PI) = 0.766 (0.435−0.781)
Cumulative Probability of Nausea/vomiting at 12 week (median and 95% PI) = 0.799 (0.531−0.817)
Cumulative Probability of Nausea/vomiting at 38 week (median and 95% PI) = 0.907 (0.799−0.92)
95% Prediction Interval
Median Prediction

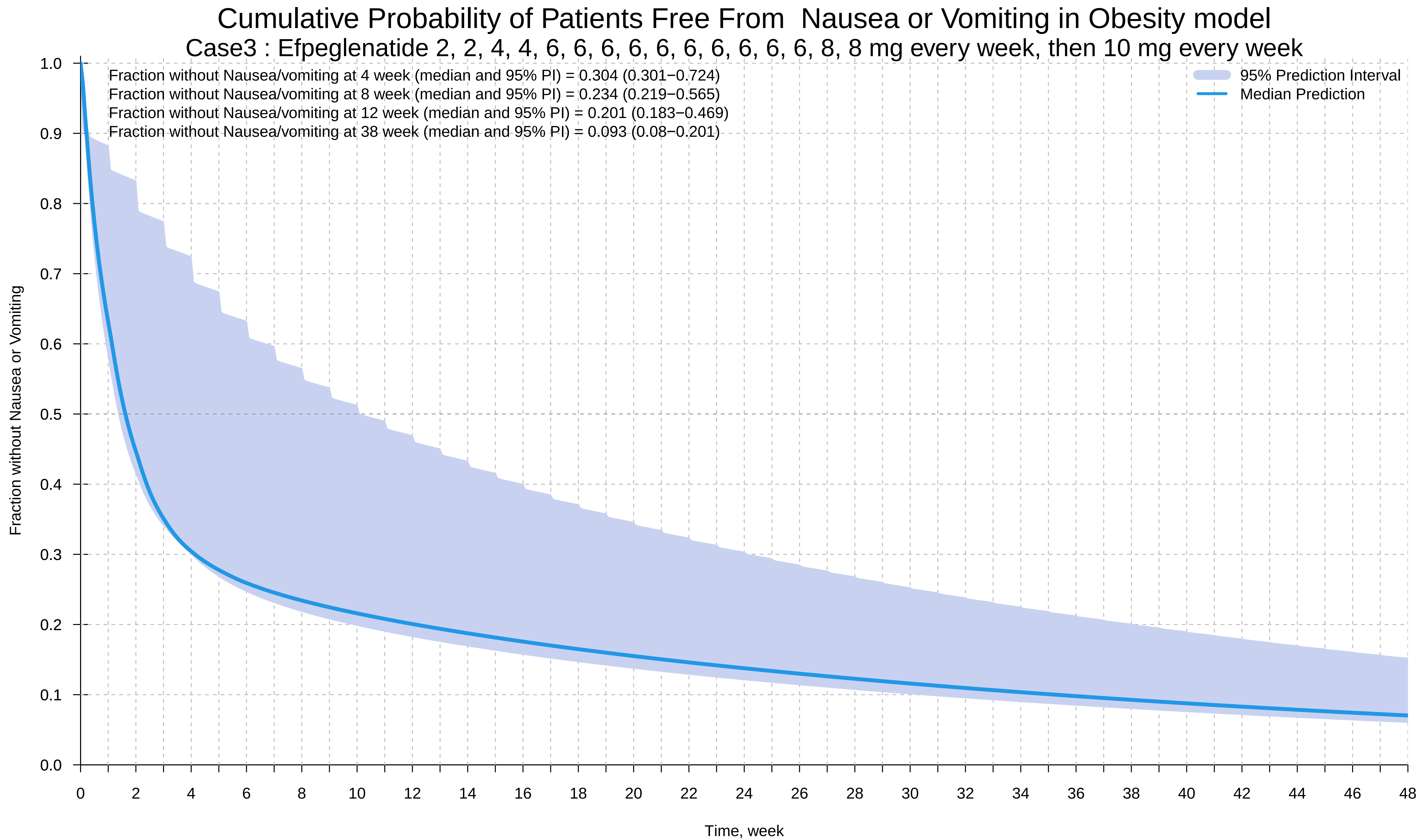
Cumulative Probability of Patients Free From Nausea or Vomiting in Obesity model
Case3 : Efpeglenatide 2, 2, 4, 4, 6, 6, 6, 6, 6, 6, 6, 6, 6, 6, 8, 8 mg every week, then 10 mg every week
Fraction without Nausea/vomiting at 4 week (median and 95% PI) = 0.304 (0.301−0.724)
Fraction without Nausea/vomiting at 8 week (median and 95% PI) = 0.234 (0.219−0.565)
Fraction without Nausea/vomiting at 12 week (median and 95% PI) = 0.201 (0.183−0.469)
Fraction without Nausea/vomiting at 38 week (median and 95% PI) = 0.093 (0.08−0.201)
95% Prediction Interval
Median Prediction
Fraction without Nausea or Vomiting
Time, week
0.0
0.1
0.2
0.3
0.4
0.5
0.6
0.7
0.8
0.9
1.0
0
2
4
6
8
10
12
14
16
18
20
22
24
26
28
30
32
34
36
38
40
42
44
46
48

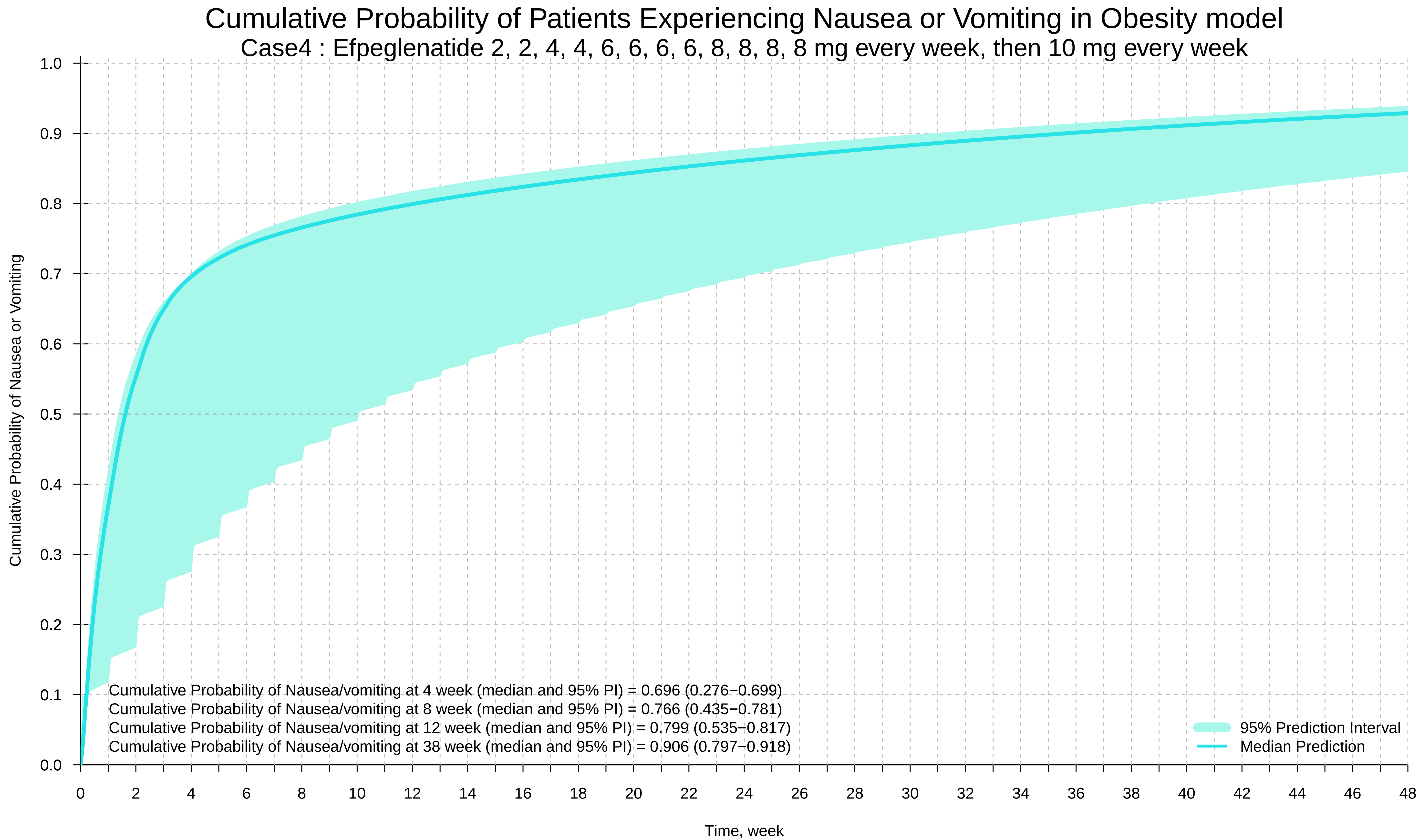

Cumulative Probability of Patients Experiencing Nausea or Vomiting in Obesity model
Case4 : Efpeglenatide 2, 2, 4, 4, 6, 6, 6, 6, 8, 8, 8, 8 mg every week, then 10 mg every week
Cumulative Probability of Nausea or Vomiting
Time, week
0.0
0.1
0.2
0.3
0.4
0.5
0.6
0.7
0.8
0.9
1.0
0
2
4
6
8
10
12
14
16
18
20
22
24
26
28
30
32
34
36
38
40
42
44
46
48
Cumulative Probability of Nausea/vomiting at 4 week (median and 95% PI) = 0.696 (0.276−0.699)
Cumulative Probability of Nausea/vomiting at 8 week (median and 95% PI) = 0.766 (0.435−0.781)
Cumulative Probability of Nausea/vomiting at 12 week (median and 95% PI) = 0.799 (0.535−0.817)
Cumulative Probability of Nausea/vomiting at 38 week (median and 95% PI) = 0.906 (0.797−0.918)
95% Prediction Interval
Median Prediction

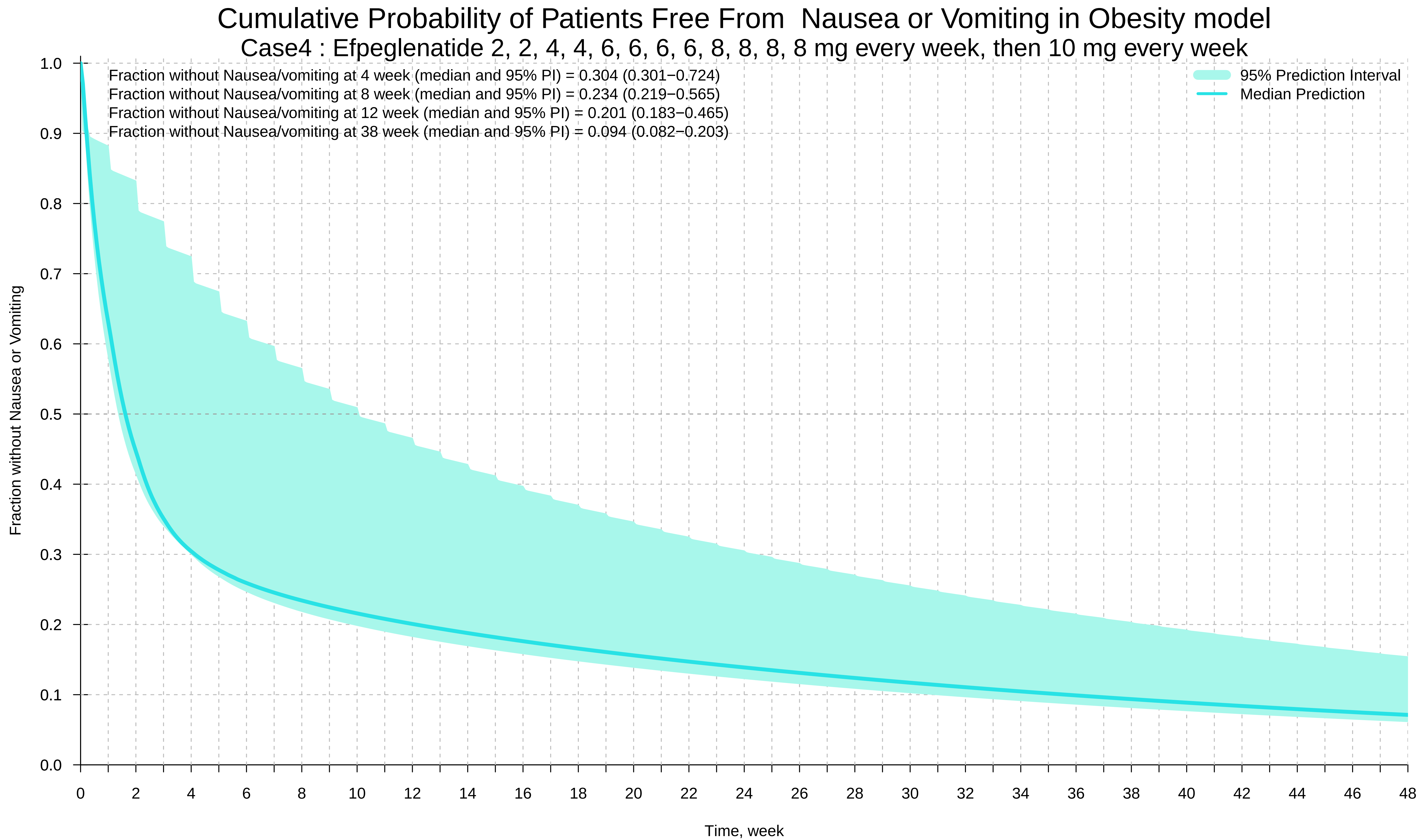
Cumulative Probability of Patients Free From Nausea or Vomiting in Obesity model
Case4 : Efpeglenatide 2, 2, 4, 4, 6, 6, 6, 6, 8, 8, 8, 8 mg every week, then 10 mg every week
Fraction without Nausea/vomiting at 4 week (median and 95% PI) = 0.304 (0.301−0.724)
Fraction without Nausea/vomiting at 8 week (median and 95% PI) = 0.234 (0.219−0.565)
Fraction without Nausea/vomiting at 12 week (median and 95% PI) = 0.201 (0.183−0.465)
Fraction without Nausea/vomiting at 38 week (median and 95% PI) = 0.094 (0.082−0.203)
95% Prediction Interval
Median Prediction
Fraction without Nausea or Vomiting
Time, week
0.0
0.1
0.2
0.3
0.4
0.5
0.6
0.7
0.8
0.9
1.0
0
2
4
6
8
10
12
14
16
18
20
22
24
26
28
30
32
34
36
38
40
42
44
46
48

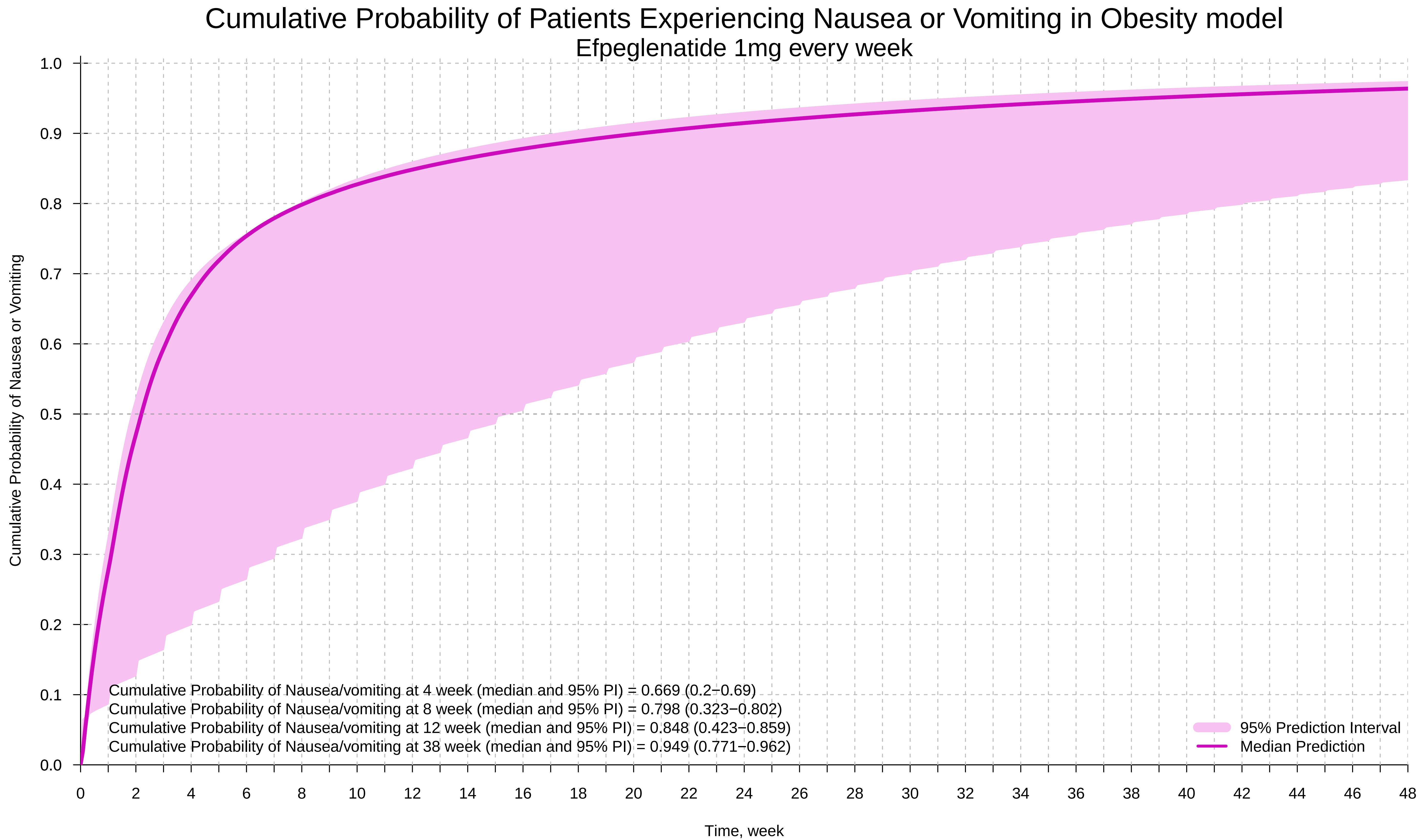
Cumulative Probability of Patients Experiencing Nausea or Vomiting in Obesity model
Efpeglenatide 1mg every week
Cumulative Probability of Nausea or Vomiting
Time, week
Cumulative Probability of Nausea/vomiting at 4 week (median and 95% PI) = 0.669 (0.2−0.69)
Cumulative Probability of Nausea/vomiting at 8 week (median and 95% PI) = 0.798 (0.323−0.802)
Cumulative Probability of Nausea/vomiting at 12 week (median and 95% PI) = 0.848 (0.423−0.859)
Cumulative Probability of Nausea/vomiting at 38 week (median and 95% PI) = 0.949 (0.771−0.962)
95% Prediction Interval
Median Prediction

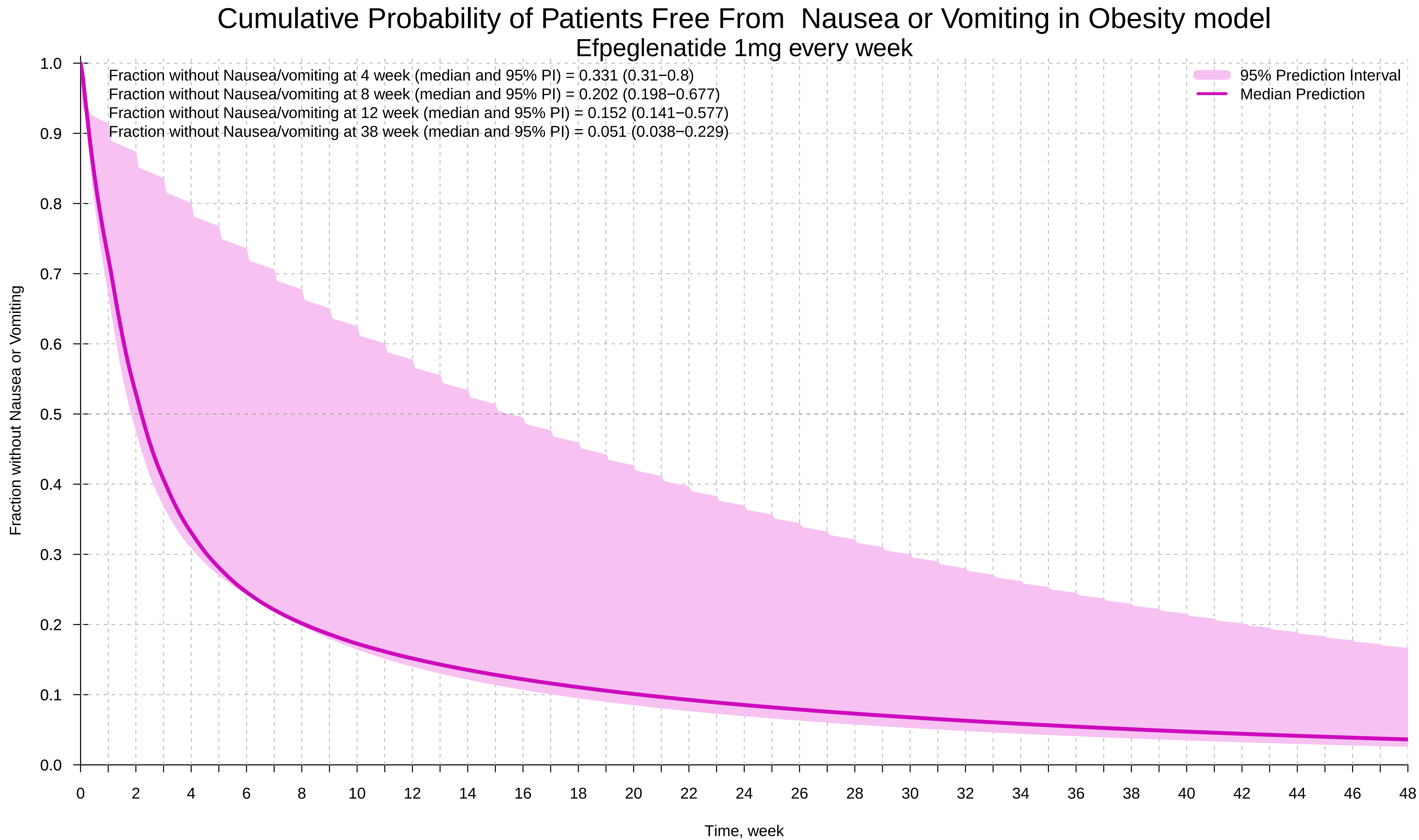
Cumulative Probability of Patients Free From Nausea or Vomiting in Obesity model
Efpeglenatide 1mg every week
Fraction without Nausea/vomiting at 4 week (median and 95% PI) = 0.331 (0.31−0.8)
Fraction without Nausea/vomiting at 8 week (median and 95% PI) = 0.202 (0.198−0.677)
Fraction without Nausea/vomiting at 12 week (median and 95% PI) = 0.152 (0.141−0.577)
Fraction without Nausea/vomiting at 38 week (median and 95% PI) = 0.051 (0.038−0.229)
95% Prediction Interval
Median Prediction
Fraction without Nausea or Vomiting
0.0
0.1
0.2
0.3
0.4
0.5
0.6
0.7
0.8
0.9
1.0
0
2
4
6
8
10
12
14
16
18
20
22
24
26
28
30
32
34
36
38
40
42
44
46
48
Time, week

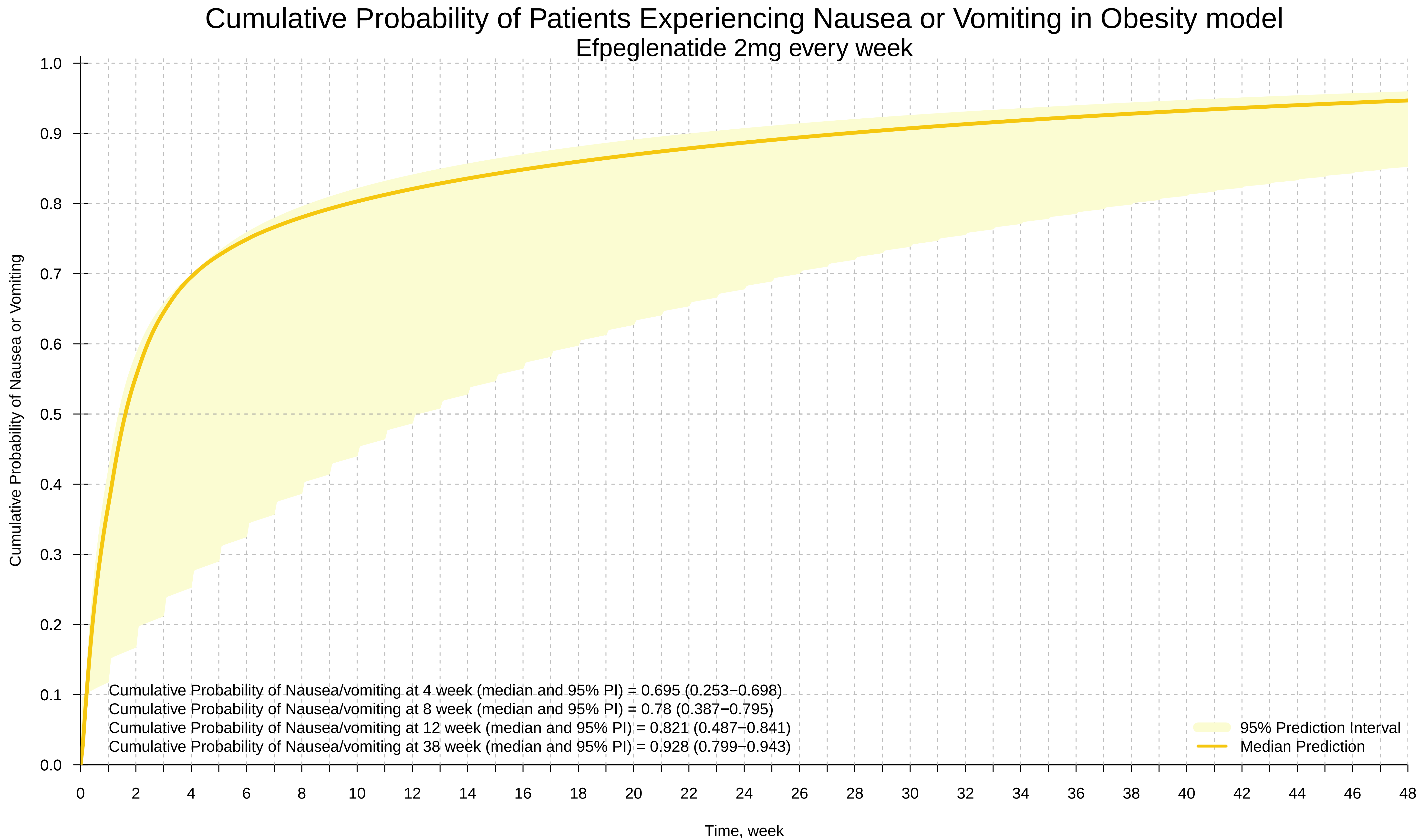
Cumulative Probability of Patients Experiencing Nausea or Vomiting in Obesity model
Efpeglenatide 2mg every week
Cumulative Probability of Nausea or Vomiting
Time, week
Cumulative Probability of Nausea/vomiting at 4 week (median and 95% PI) = 0.695 (0.253−0.698)
Cumulative Probability of Nausea/vomiting at 8 week (median and 95% PI) = 0.78 (0.387−0.795)
Cumulative Probability of Nausea/vomiting at 12 week (median and 95% PI) = 0.821 (0.487−0.841)
Cumulative Probability of Nausea/vomiting at 38 week (median and 95% PI) = 0.928 (0.799−0.943)
95% Prediction Interval
Median Prediction

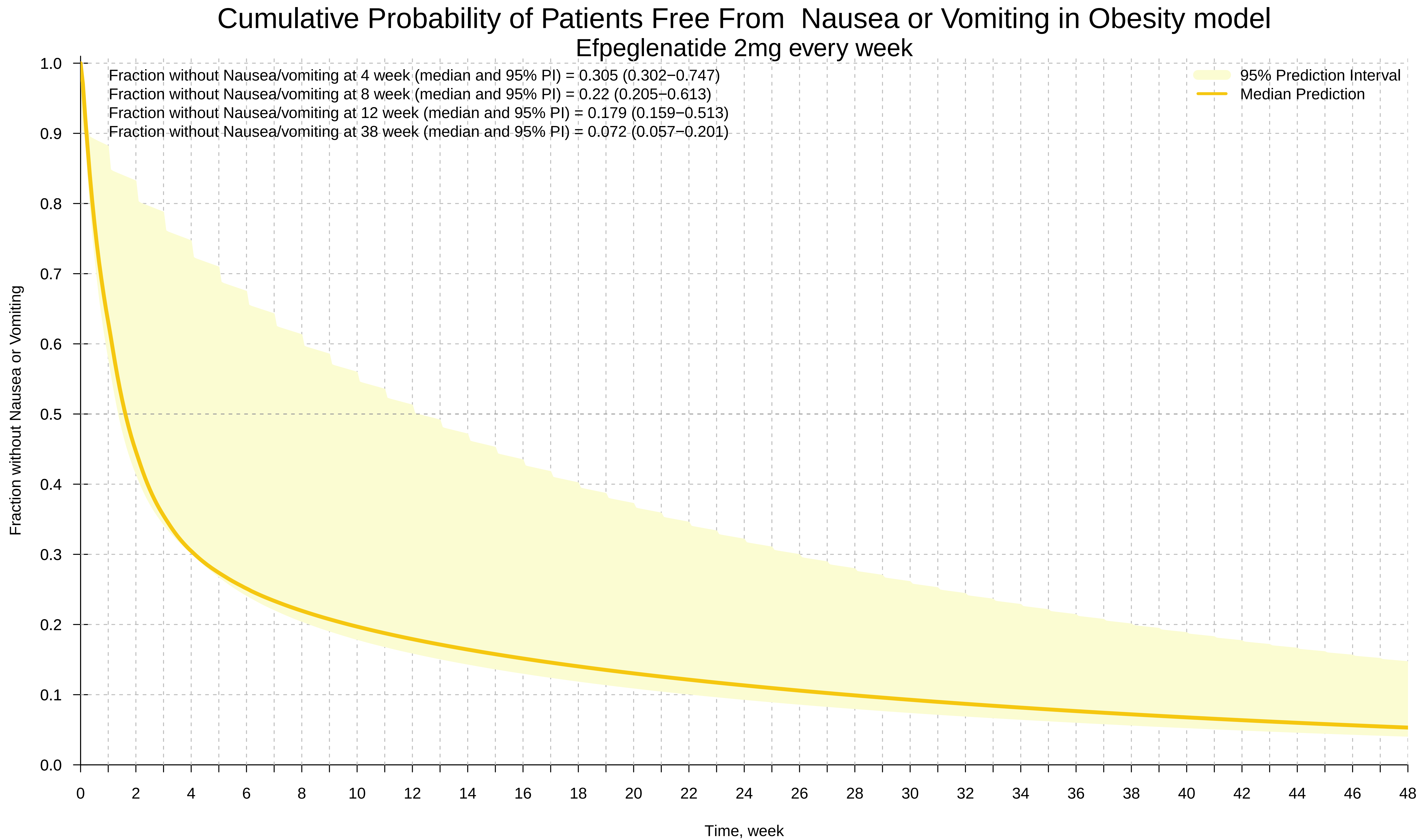
Cumulative Probability of Patients Free From Nausea or Vomiting in Obesity model
Efpeglenatide 2mg every week
Fraction without Nausea/vomiting at 4 week (median and 95% PI) = 0.305 (0.302−0.747)
Fraction without Nausea/vomiting at 8 week (median and 95% PI) = 0.22 (0.205−0.613)
Fraction without Nausea/vomiting at 12 week (median and 95% PI) = 0.179 (0.159−0.513)
Fraction without Nausea/vomiting at 38 week (median and 95% PI) = 0.072 (0.057−0.201)
95% Prediction Interval
Median Prediction
Fraction without Nausea or Vomiting
Time, week
0.0
0.1
0.2
0.3
0.4
0.5
0.6
0.7
0.8
0.9
1.0
0
2
4
6
8
10
12
14
16
18
20
22
24
26
28
30
32
34
36
38
40
42
44
46
48

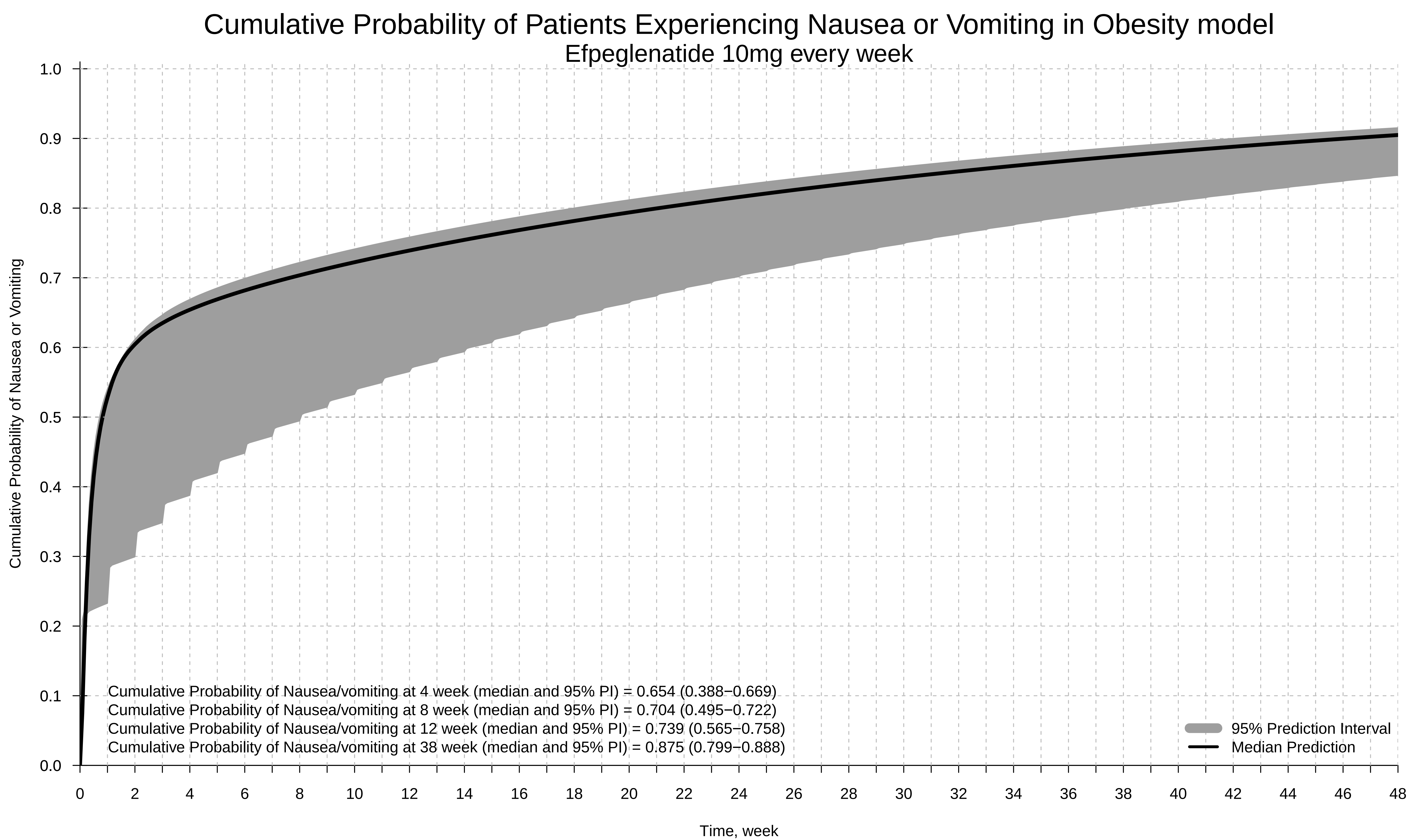
Cumulative Probability of Patients Experiencing Nausea or Vomiting in Obesity model
Efpeglenatide 10mg every week
Cumulative Probability of Nausea or Vomiting
Time, week
Cumulative Probability of Nausea/vomiting at 4 week (median and 95% PI) = 0.654 (0.388−0.669)
Cumulative Probability of Nausea/vomiting at 8 week (median and 95% PI) = 0.704 (0.495−0.722)
Cumulative Probability of Nausea/vomiting at 12 week (median and 95% PI) = 0.739 (0.565−0.758)
Cumulative Probability of Nausea/vomiting at 38 week (median and 95% PI) = 0.875 (0.799−0.888)
95% Prediction Interval
Median Prediction

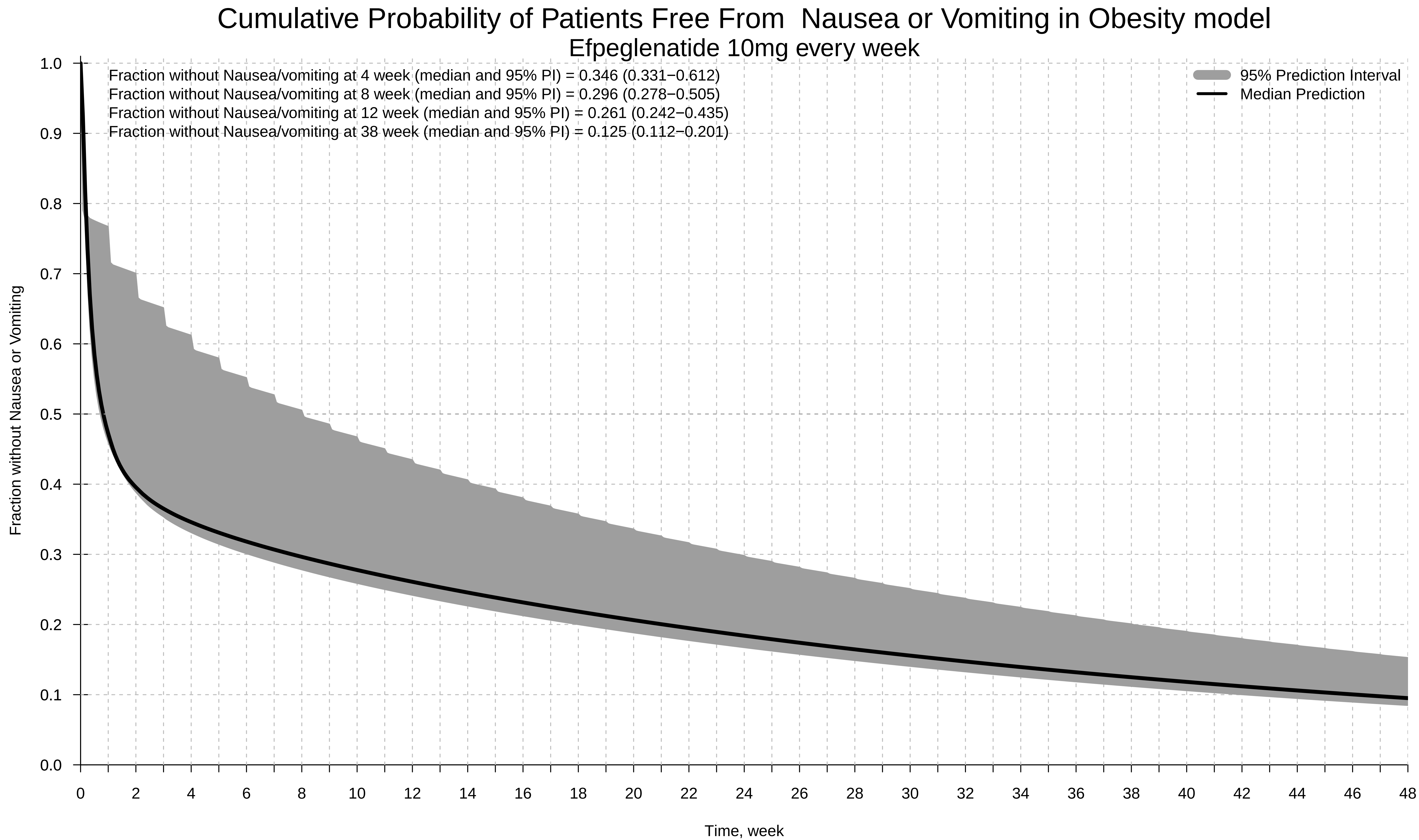
Cumulative Probability of Patients Free From Nausea or Vomiting in Obesity model
Efpeglenatide 10mg every week
Fraction without Nausea/vomiting at 4 week (median and 95% PI) = 0.346 (0.331−0.612)
Fraction without Nausea/vomiting at 8 week (median and 95% PI) = 0.296 (0.278−0.505)
Fraction without Nausea/vomiting at 12 week (median and 95% PI) = 0.261 (0.242−0.435)
Fraction without Nausea/vomiting at 38 week (median and 95% PI) = 0.125 (0.112−0.201)
95% Prediction Interval
Median Prediction
Fraction without Nausea or Vomiting
Time, week
0.0
0.1
0.2
0.3
0.4
0.5
0.6
0.7
0.8
0.9
1.0
0
2
4
6
8
10
12
14
16
18
20
22
24
26
28
30
32
34
36
38
40
42
44
46
48

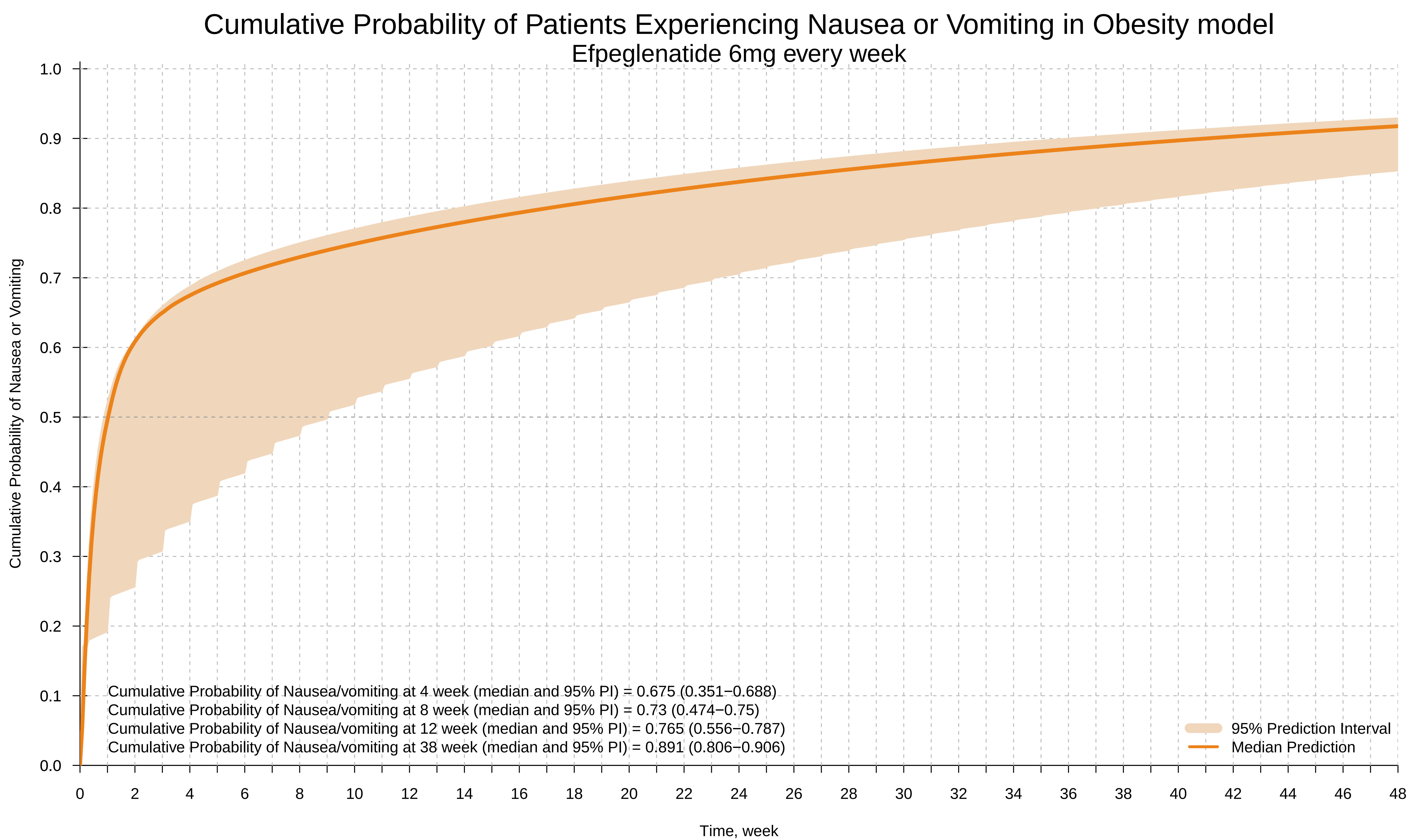
Cumulative Probability of Patients Experiencing Nausea or Vomiting in Obesity model
Efpeglenatide 6mg every week
Cumulative Probability of Nausea or Vomiting
Time, week
Cumulative Probability of Nausea/vomiting at 4 week (median and 95% PI) = 0.675 (0.351−0.688)
Cumulative Probability of Nausea/vomiting at 8 week (median and 95% PI) = 0.73 (0.474−0.75)
Cumulative Probability of Nausea/vomiting at 12 week (median and 95% PI) = 0.765 (0.556−0.787)
Cumulative Probability of Nausea/vomiting at 38 week (median and 95% PI) = 0.891 (0.806−0.906)
95% Prediction Interval
Median Prediction

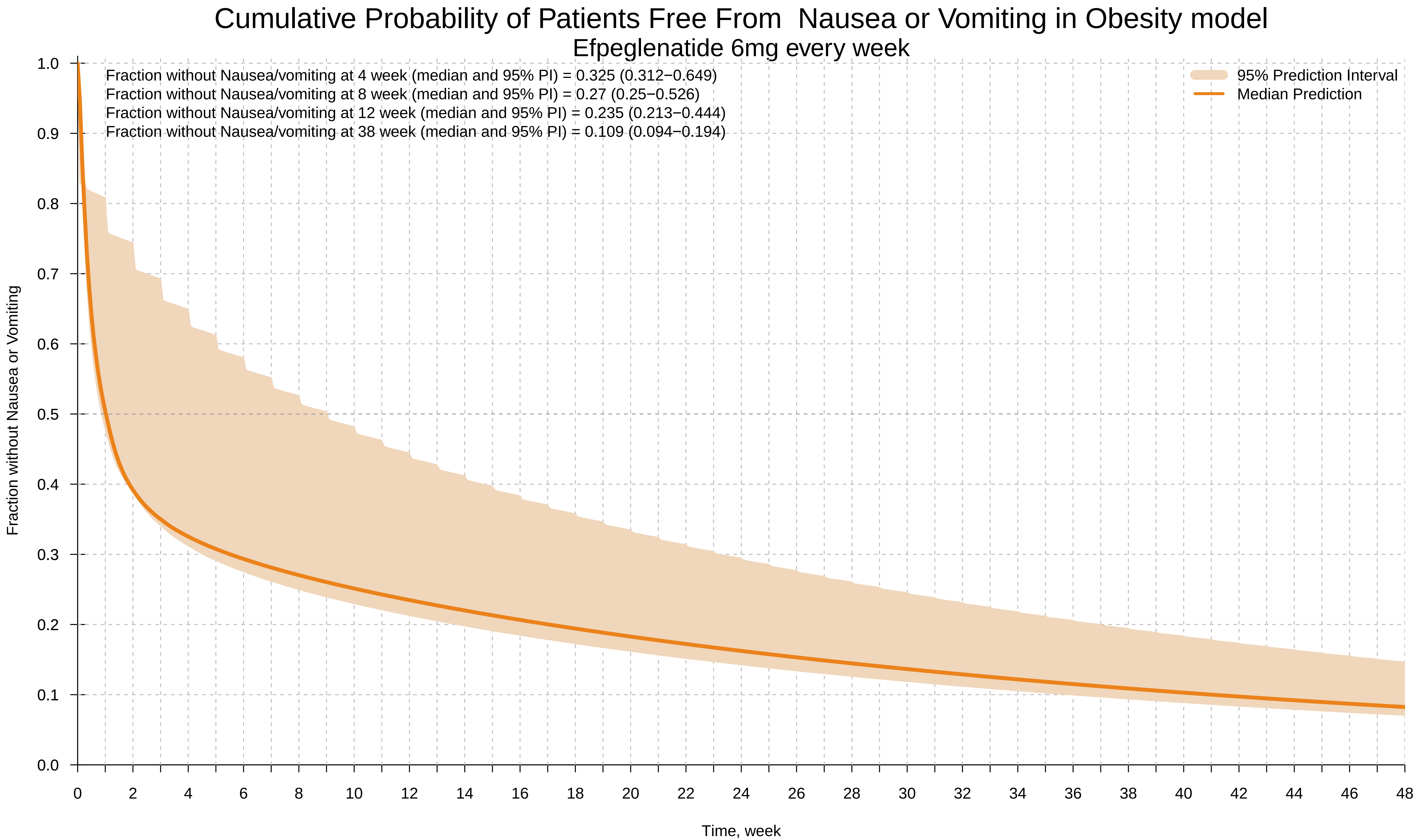

Cumulative Probability of Patients Free From Nausea or Vomiting in Obesity model
Efpeglenatide 6mg every week
Fraction without Nausea/vomiting at 4 week (median and 95% PI) = 0.325 (0.312−0.649)
Fraction without Nausea/vomiting at 8 week (median and 95% PI) = 0.27 (0.25−0.526)
Fraction without Nausea/vomiting at 12 week (median and 95% PI) = 0.235 (0.213−0.444)
Fraction without Nausea/vomiting at 38 week (median and 95% PI) = 0.109 (0.094−0.194)
95% Prediction Interval
Median Prediction
Fraction without Nausea or Vomiting
1.0
0.9
0.8
0.7
0.6
0.5
0.4
0.3
0.2
0.1
0.0
0
2
4
6
8
10
12
14
16
18
20
22
24
26
28
30
32
34
36
38
40
42
44
46
48
Time, week

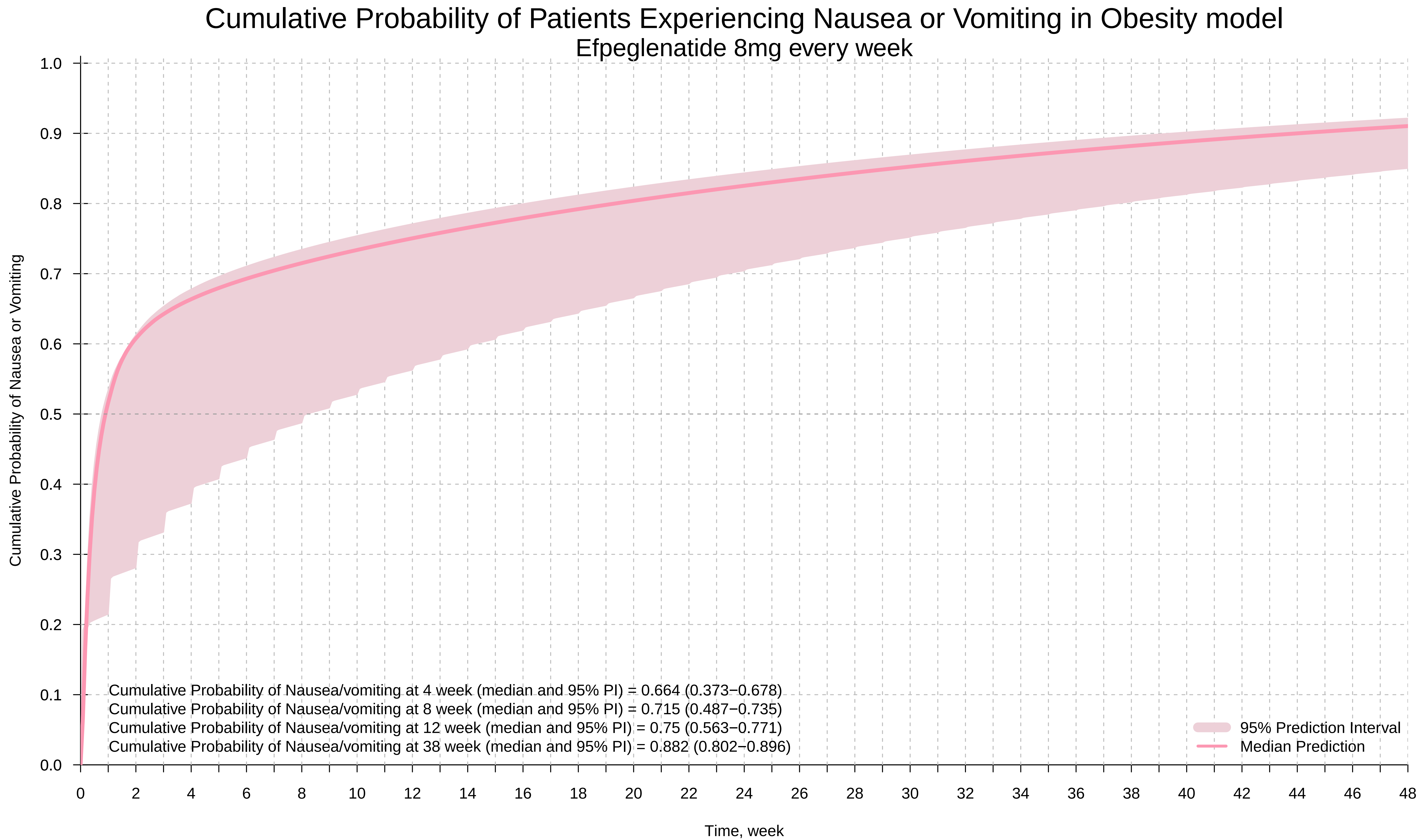
Cumulative Probability of Patients Experiencing Nausea or Vomiting in Obesity model
Efpeglenatide 8mg every week
Cumulative Probability of Nausea or Vomiting
Time, week
Cumulative Probability of Nausea/vomiting at 4 week (median and 95% PI) = 0.664 (0.373−0.678)
Cumulative Probability of Nausea/vomiting at 8 week (median and 95% PI) = 0.715 (0.487−0.735)
Cumulative Probability of Nausea/vomiting at 12 week (median and 95% PI) = 0.75 (0.563−0.771)
Cumulative Probability of Nausea/vomiting at 38 week (median and 95% PI) = 0.882 (0.802−0.896)
95% Prediction Interval
Median Prediction

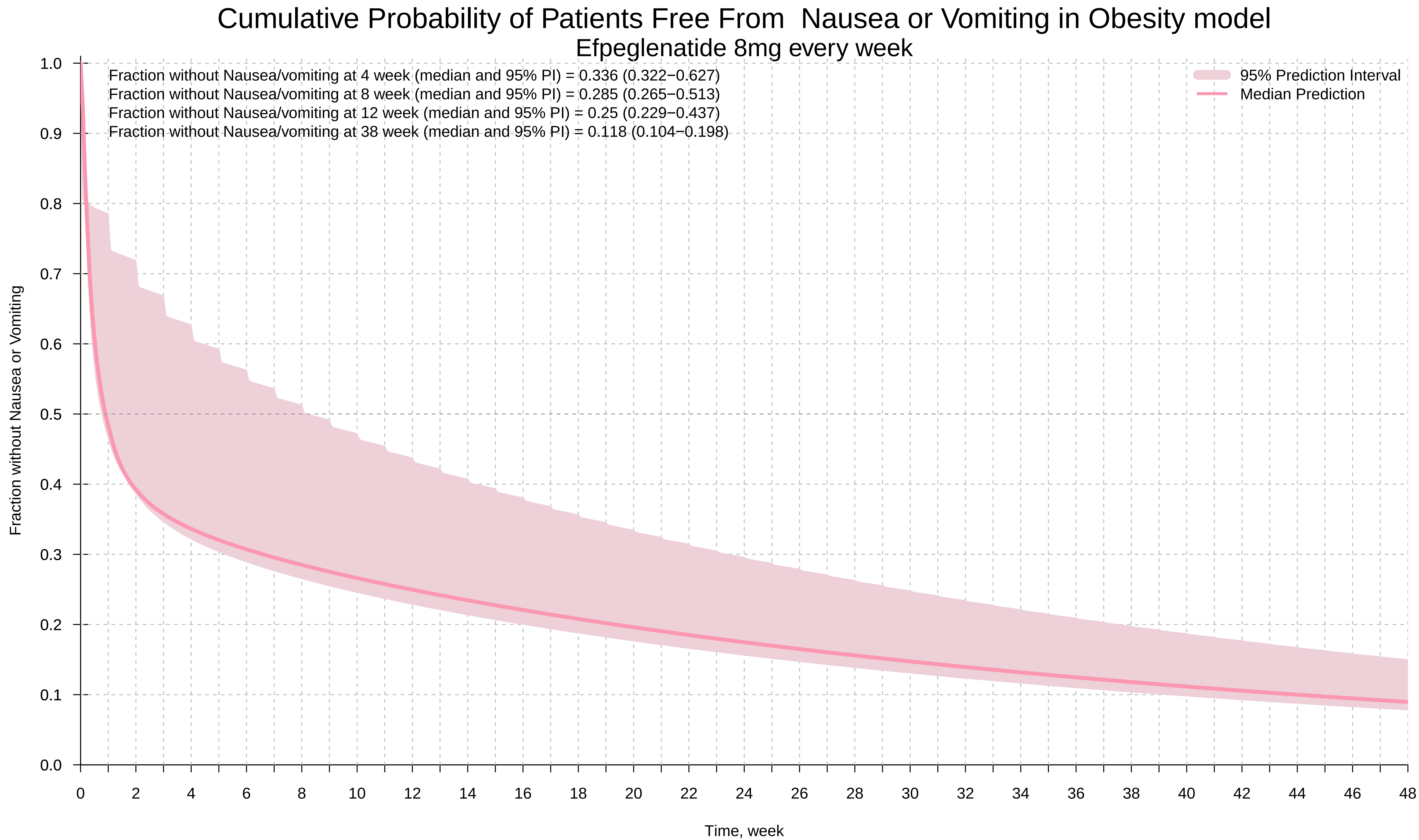
Cumulative Probability of Patients Free From Nausea or Vomiting in Obesity model
Efpeglenatide 8mg every week
Fraction without Nausea/vomiting at 4 week (median and 95% PI) = 0.336 (0.322−0.627)
Fraction without Nausea/vomiting at 8 week (median and 95% PI) = 0.285 (0.265−0.513)
Fraction without Nausea/vomiting at 12 week (median and 95% PI) = 0.25 (0.229−0.437)
Fraction without Nausea/vomiting at 38 week (median and 95% PI) = 0.118 (0.104−0.198)
95% Prediction Interval
Median Prediction
Fraction without Nausea or Vomiting
Time, week
