# Supplementary material for: Exposure-driven tolerance modeling of efpeglenatide-induced nausea or vomiting using repeated time-to-event analysis: implications for dose-escalation strategies
Source: Front Pharmacol. 2026 Sep 22;17:1846164. doi: 10.3389/fphar.2026.1846164 (PMC13639893; doi:10.3389/fphar.2026.1846164)
Supplement: Supplementary file 2 [file Supplementaryfile1.docx]

***Supplementary Material***

**Supplementary 1. Mathematical Formulation of the Refined RTTE Model** [19-20]

*T* denotes the event-time random variable measured relative to the first drug administration during the observation period extending to the last follow-up. The survival function $S\left( t \right)$ is described by Equation 1, where $P\left( T>t \right)$ represents the cumulative probability that the first event has not yet occurred until time $t$. By definition, $S\left( t_{0} \right)=1$. $P\left( T>t \right)$ can be expressed as a function of the hazard $h\left( t \right)$ integrated over time.

$$S\left( t \right)=P\left( T>t \right)={exp}^{\left[ -\int_{t_{0}}^{t} h\left( t \right)dt \right]}$$

Eq. 1

The hazard, denoted as $h\left( t \right)$, is expressed in Equation 2, which represents the instantaneous event rate at time $t$, conditional on the event not having occurred up to that time $t$. It quantifies the instantaneous risk (or occurrence rate) of an event at time $t$ among individuals who are still event-free just before $t$. This function quantifies the instantaneous risk of an event at time.

$$h\left( t \right)=P\left( T=t|T\geq t \right)=\lim_{\Delta t\to0} \frac{P\left( t\leq T<t+\Delta t|T\geq t \right)}{\Delta t}$$

Eq. 2

The cumulative hazard $H\left( t \right)$ represents the cumulative hazard of an event occurring by time $t$ Moreover, when repetitions of the event are possible, $H\left( t \right)$ can be interpreted as the expected number of events occurring during the interval from the time origin $t_{0}$ to $t$. As shown in Equation 3:

$$H\left( t \right)=\int_{t_{0}}^{t} h\left( u \right)du$$

Eq. 3

$H\left( t \right)$ represents the accumulated hazard, where a unit value signifies the standard amount of risk sufficient to produce one event on average.

The cumulative probability of an event occurring until time $t$*,* and its relationship with $S(t)$*,* is given by Equation 4:

$$F\left( t \right)=P\left( T<t \right)=1-S(t)$$

Eq. 4

The probability density function of an event occurring at a given time $t$ can be obtained by differentiating $F\left( t \right)$, and its relationship with $S\left( t \right)$and $h\left( t \right)$ is summarized by Equation 5:

$$f\left( t \right)=h(t)\cdot S(t) =-\frac{dS(t)}{dt}$$

Eq.5

In the context of maximum likelihood estimation, $f$ serves as the individual likelihood contribution.

Suppose that multiple events may occur in an individual and let $t_{i}$ denote the time of the $i$-th event. In RTTE analysis, the survival between two successive event times $t_{i}$ and $t_{i+1}$ is defined as the cumulative probability that no event occurs up to time $t_{i+1}$, conditional on survival up to time $t_{i}$. Therefore, the survival function in RTTE models is conditional and can be expressed as:

$$S(t_{i+1}|t_{i})= P\left( T>t_{i+1} | T>t_{i} \right)$$

Eq. 6

Conditioning on survival up to $t_{i}$, the baseline time origin is reset at the previous event time $t_{i}$, and the conditional survival probability over the interval $(t_{i}$, $t_{i+1}]$ is expressed as Equation 7:

$$S(t_{i+1}|t_{i})={exp}^{-\int_{t_{i}}^{t_{i+1}} h\left( t \right)dt}$$

Eq. 7

In repeated exact time-to-event analysis, $h\left( t_{i+1} \right)$ can be redefined as the instantaneous event rate at time $t_{i+1,}$ conditional on survival up to time $t_{i}$. Treating the previous event time $t_{i}$ as a realized constant, the conditional hazard can be expressed as the derivative of the conditional cumulative hazard:

$$h\left( t_{i+1}|t_{i} \right)=\left. \frac{d}{dt}H(t\mid t_{i}) \right|_{t=t_{i+1}}$$

Eq. 8

To preserve the dependence structure between successive events, $t_{i}$ and $t_{i+1}$ were treated as two realized values of an event-time random variable associated with $h\left( t \right)$, and the conditional interval-averaged hazard was defined as follows:

$$h\left( t_{i+1}|t_{i} \right)=\frac{\int_{t_{0}}^{t_{i+1}} h\left( u \right)du-\int_{t_{0}}^{t_{i}} h\left( u \right)du}{t_{i+1}-t_{i}}$$

Eq. 9

As shown in Equation 9, the conditional hazard $h(t_{i+1}\mid t_{i})$ is defined as the mean rate of change of the cumulative hazard (CUMHAZ) over the interval $(t_{i},t_{i+1}]$, calculated as the difference between the cumulative hazard evaluated at $t_{i+1}$ and at $t_{i}$, divided by the elapsed time.

In repeated exact time-to-event analysis, the conditional probability density function of an event occurring at time $t_{i+1}$, $f(t_{i+1})$, can be expressed conditional on survival up to time $t_{i}$. The conditional probability density can therefore be written as:

| $f(t_{i+1}$\|$t_{i}$)=$S(t_{i+1}$\|$t_{i}$) $\cdot h(t_{i+1}$\|$t_{i}$) | Eq. 10 |
| --- | --- |

For interval-censored RTTE data—where occurrences are documented within 24-hour windows rather than at exact timestamps—conditional probabilities were derived by conditioning on the event occurring after the endpoint of the preceding interval $(t_{i}, {t^{'}}_{i}]$. In interval-censored data, the conditional survival function at the current interval $(t_{i+1}, {t^{'}}_{i+1}]$ is defined with respect to the endpoint of the previous event interval, $t_{i}^{'}$, as follows:

$$S(t_{i+1}|{t'}_{i})= P\left( T>t_{i+1} | T>{t'}_{i} \right)={exp}^{-\int_{{t'}_{i}}^{t_{i+1}} h\left( t \right)dt}$$

Eq. 11

Accordingly, the endpoint ${t^{'}}_{i}$ of the previous event interval $(t_{i}, {t^{'}}_{i}]$ was used as the reference point for conditioning. The conditional cumulative hazard was evaluated from ${t^{'}}_{i}$ to each boundary of the current interval $(t_{i+1}, {t^{'}}_{i+1}]$. The cumulative hazard over the current interval was then obtained by subtracting the cumulative hazard up to $t_{i+1}$ from that up to ${t'}_{i+1}$. Because both quantities are referenced to the same preceding endpoint ${t^{'}}_{i}$, the common cumulative-hazard contribution over $({t'}_{i}, t_{i+1}]$, cancels, leaving only the cumulative hazard accumulated within the current interval $(t_{i+1}, {t^{'}}_{i+1}]$, as expressed in Equation 12:

$$H\left( t_{i+1}, {t^{'}}_{i+1}|{t^{'}}_{i} \right)=\int_{t_{i+1}}^{{t^{'}}_{i+1}} h\left( t|{t^{'}}_{i} \right)dt=H\left( t_{i+1}, {t^{'}}_{i+1} \right)$$

Eq. 12

In recurrent interval-censored time-to-event analysis, the conditional probability of an event occurring within the interval $(t_{i+1}, {t^{'}}_{i+1}]$, following a prior event in $(t_{i}, {t^{'}}_{i}]$, can be formulated as follows, given survival up to the start of the current interval $t_{i+1}$:

$$F\left( t_{i+1}, {t^{'}}_{i+1} | {t^{'}}_{i} \right)=S\left( t_{i+1} | {t^{'}}_{i} \right)\cdot H\left( t_{i+1}, {t^{'}}_{i+1} \right)$$

Eq. 13

Given that $H\left( t_{i+1}, {t^{'}}_{i+1} \right)$ represents the cumulative hazard for a single event within the interval $(t_{i+1}, {t^{'}}_{i+1}]$, the cumulative hazard for $n$ independent and identical events can be derived based on combinatorial principles. Accordingly, the conditional probability of observing exactly $n$ events in the current interval, following the end point ${t^{'}}_{i}$ of the previous interval $(t_{i}, {t^{'}}_{i}]$, is formulated as:

$$\left. F\left( t_{i+1}, {t^{'}}_{i+1} | {t^{'}}_{i} \right) \right|_{event=n}=S\left( t_{i+1} | {t^{'}}_{i} \right)\cdot\frac{1}{n!}\left\{ H\left( t_{i+1}, {t^{'}}_{i+1} \right) \right\}^{n}$$

Eq. 14

The termination of the evaluation period (i.e., the total observation duration for each participant) was treated as a right-censoring point, under the assumption that no further events were recorded for the participant after this threshold.

Therefore, the probability beyond the censoring time was defined as the likelihood of no event occurring, conditional on the event occurring after the $n$th last event. This probability was determined using the survival function, as presented in equation 15.

$$f(t|t_{n})=S(t|t_{n})$$

Eq. 15

For censored events, where no occurrence is documented before the end of the observation period, the likelihood is specifically formulated as the conditional survival function, $S(t|t_{n})$, which represents the probability of remaining event-free up to the censoring time.

Therefore, $f$ represents the probability density of an event occurrence for participants who experienced the event, and the survival probability for those who were censored.

The mathematical formulations described in equations 6, 9, 11, 12, 14, and 15 were integrated into the NONMEM^®^ control stream. These functions were then numerically evaluated using the built-in differential equation solver to account for the time-varying nature of the hazard and survival estimates.

**Supplementary 2. Comparison of candidate tolerance structures for** $\boldsymbol{E}_{\boldsymbol{max}}$ **and** $\boldsymbol{E}\boldsymbol{C}_{\boldsymbol{50}}$ **in the pooled total model**

**(a) Candidate tolerance structures and corresponding hazard equations evaluated for the pooled total model.**

| **Candidate tolerance structure** | **Tolerance function** | **Hazard equation** |
| --- | --- | --- |
| **Dual tolerance on** $\boldsymbol{E}_{\boldsymbol{max}}$ **and** $\boldsymbol{E}\boldsymbol{C}_{\boldsymbol{50}}$**:**  **separate** $\boldsymbol{TOL}_{\boldsymbol{max}}$ **and shared** $\boldsymbol{T}\boldsymbol{C}_{\boldsymbol{50}}$ **(Final model)** | $Tol_{1}\left( AUC \right)=\frac{TOL_{max,1}\cdot AUC}{TC_{50}+AUC}$  $Tol_{2}\left( AUC \right)=\frac{TOL_{max,2}\cdot AUC}{TC_{50}+AUC}$ | $h(t)=\theta_{0}\cdot\exp\left\{ \exp(-\theta_{ktol}\cdot t)+\frac{E_{max}\cdot(1-Tol_{1}(AUC))\cdot C_{p}}{EC_{50}\cdot(1+Tol_{2}(AUC))+C_{p}} \right\}$ |
| **Dual tolerance on** $\boldsymbol{E}_{\boldsymbol{max}}$ **and** $\boldsymbol{E}\boldsymbol{C}_{\boldsymbol{50}}$**:**  **separate** $\boldsymbol{TOL}_{\boldsymbol{max}}$ **and** $\boldsymbol{T}\boldsymbol{C}_{\boldsymbol{50}}$ | $Tol_{1}\left( AUC \right)=\frac{TOL_{max,1}\cdot AUC}{TC_{50,1}+AUC}$  $Tol_{2}(AUC)=\frac{TOL_{max,2}\cdot AUC}{TC_{50,2}+AUC}$ | $h(t)=\theta_{0}\cdot\exp\left\{ \exp(-\theta_{ktol}\cdot t)+\frac{E_{max}\cdot(1-Tol_{1}(AUC))\cdot C_{p}}{EC_{50}\cdot(1+Tol_{2}(AUC))+C_{p}} \right\}$ |
| **Dual tolerance on** $\boldsymbol{E}_{\boldsymbol{max}}$ **and** $\boldsymbol{E}\boldsymbol{C}_{\boldsymbol{50}}$**: shared** $\boldsymbol{TOL}_{\boldsymbol{max}}$ **and** $\boldsymbol{T}\boldsymbol{C}_{\boldsymbol{50}}$ | $Tol(AUC)=\frac{TOL_{max}\cdot AUC}{TC_{50}+AUC}$ | $h(t)=\theta_{0}\cdot\exp\left\{ \exp(-\theta_{ktol}\cdot t)+\frac{E_{max}\cdot(1-Tol(AUC))\cdot C_{p}}{EC_{50}\cdot(1+Tol(AUC))+C_{p}} \right\}$ |
| **Tolerance on** $\boldsymbol{E}_{\boldsymbol{max}}$ **only** |  | $h(t)=\theta_{0}\cdot\exp\left\{ \exp(-\theta_{ktol}\cdot t)+\frac{E_{max}\cdot(1-Tol(AUC))\cdot C_{p}}{EC_{50}+C_{p}} \right\}$ |
| **Tolerance on** $\boldsymbol{E}\boldsymbol{C}_{\boldsymbol{50}}$ **only** |  | $h(t)=\theta_{0}\cdot\exp\left\{ \exp(-\theta_{ktol}\cdot t)+\frac{E_{max}\cdot C_{p}}{EC_{50}\cdot(1+Tol(AUC))+C_{p}} \right\}$ |

Abbreviations: AUC, cumulative area under the concentration–time curve; $C_{p}$, time-varying drug concentration; $\theta_{E_{max}}$, maximum drug effect; $\theta_{{EC}_{50}}$, concentration producing 50% of Emax; $\theta_{{TOL}_{max}}$, maximum tolerance effect; $\theta_{{TC}_{50}}$, cumulative AUC producing 50% of TOLmax. $\theta_{0}$ denotes the baseline hazard, and $\theta_{ktol}$ denotes the first-order rate constant describing the time course of the non-drug effect. $Tol_{1}$ and $Tol_{2}$ indicate tolerance functions applied to Emax and EC50, respectively. For shared-tolerance structures, a common $Tol$ function was applied to both Emax and EC50.

The first candidate structure corresponds to the final pooled total model used as the reference model.

**(b) Parameter estimates for candidate tolerance structures evaluated for the pooled total model.**

| **Total model** | | | | | | |
| --- | --- | --- | --- | --- | --- | --- |
| **Parameter** | **Unit** | **Dual tolerance on** $\boldsymbol{E}_{\boldsymbol{max}}$ **and** $\boldsymbol{E}\boldsymbol{C}_{\boldsymbol{50}}$**:**  **separate** $\boldsymbol{TOL}_{\boldsymbol{max}}$ **and shared** $\boldsymbol{T}\boldsymbol{C}_{\boldsymbol{50}}$ **(Final model)** | **Dual tolerance on** $\boldsymbol{E}_{\boldsymbol{max}}$ **and** $\boldsymbol{E}\boldsymbol{C}_{\boldsymbol{50}}$**:**  **separate** $\boldsymbol{TOL}_{\boldsymbol{max}}$ **and** $\boldsymbol{T}\boldsymbol{C}_{\boldsymbol{50}}$ | **Dual tolerance on** $\boldsymbol{E}_{\boldsymbol{max}}$ **and** $\boldsymbol{E}\boldsymbol{C}_{\boldsymbol{50}}$**: shared** $\boldsymbol{TOL}_{\boldsymbol{max}}$ **and** $\boldsymbol{T}\boldsymbol{C}_{\boldsymbol{50}}$ | **Tolerance on** $\boldsymbol{E}_{\boldsymbol{max}}$ **only** | **Tolerance on** $\boldsymbol{E}\boldsymbol{C}_{\boldsymbol{50}}$ **only** |
| **OFV** | | 10,782.399 | 10,782.399 | 10,835.083 | 10,839.114 | 11,145.083 |
| **ΔOFV vs Final** | | reference | +0.00002 | +52.684 | +56.715 | +362.684 |
| **Base model: Exponential model** | | | | | | |
| $\theta_{0}$ | hr^-1^ | 0.0000702 | 0.0000702 | 0.00005613 | 0.00005607 | 0.0000655 |
| **Drug effect (Emax concentration model)** | | | | | | |
| $\theta_{E_{max}}$ | - | 5.22 | 5.22 | 3.32 | 3.3 | 1.83 |
| $\theta_{{EC}_{50}}$ | ng/mL | 81.0 | 81.0 | 0.0788 | 0.0654 | 16.8 |
| **Tolerance effect** | | | | | | |
| $\theta_{{TOL}_{max}}$ | *-* | - | - | 0.75 | - | - |
| $\theta_{{TOL}_{{max}_{1}}}$ | - | 0.829 | 0.829 | - | 0.75 | - |
| $\theta_{{TOL}_{{max}_{2}}}$ | - | 0.996 | 0.996 | - | - | 0.996 |
| $\theta_{{TC}_{50}}$ | hr$\cdot$ng/mL | 109,872 | - | 109,872 | 109,872 | 109,872 |
| $\theta_{{TC}_{{50}_{1}}}$ | hr$\cdot$ng/mL | - | 109,872 | - | - | - |
| $\theta_{{TC}_{{50}_{2}}}$ | hr$\cdot$ng/mL | - | 109,872 | - | - | - |
| **Non-drug effect** | | | | | | |
| $\theta_{ktol}$ | hr^-1^ | 0.0117 | 0.0117 | 0.00685 | 0.00655 | 0.00200595 |

Where OFV is objective function value; $\theta_{0}$ denotes the baseline hazard; $\theta_{ktol}$ is the first-order rate constant describing the time course of the non-drug effect; $\theta_{E_{max}}$ denotes the maximum drug effect in the Emax concentration–effect model; $\theta_{{EC}_{50}}$ denotes the concentration producing 50% of Emax; $\theta_{{TOL}_{max}}$ represents the maximum tolerance effect; and $\theta_{{TC}_{50}}$denotes the cumulative AUC producing 50% of the maximum tolerance effect.

For shared tolerance structures, $\theta_{{TOL}_{max}}$ and $\theta_{{TC}_{50}}$ represent common tolerance parameters applied to both $E_{max}$ and $EC_{50}$. For separate tolerance structures, subscript 1 denotes tolerance parameters for Emax, and subscript 2 denotes tolerance parameters for $EC_{50}$. A dash indicates that the parameter was not included in the corresponding model.

The first column represents the final pooled total model used as the reference model. In this structure, tolerance was applied to both $E_{max}$ and $EC_{50}$, with separate ${TOL}_{max}$ parameters and a shared $TC_{50}$. This parameterization allowed the maximum drug effect and concentration sensitivity to be attenuated to different extents, while assuming a common cumulative AUC scale for tolerance development.

The remaining columns represent alternative candidate tolerance structures evaluated during model development. These included a dual-tolerance model with separate ${TOL}_{max}$ and separate $TC_{50}$, a dual-tolerance model with shared ${TOL}_{max}$ and shared $TC_{50}$, and single-component tolerance models in which tolerance was applied only to either $E_{max}$ or $EC_{50}$. These candidate structures were compared with the final reference model to evaluate whether separate maximum tolerance effects for $E_{max}$ and $EC_{50}$ were supported while maintaining a parsimonious shared $TC_{50}$ structure.

**Supplementary 3. Visual predictive checks for candidate tolerance structures applied to** $\boldsymbol{E}_{\boldsymbol{max}}$ **and/or** $\boldsymbol{E}\boldsymbol{C}_{\boldsymbol{50}}$ **in the pooled total model**

**(a) Dual tolerance on** $\boldsymbol{E}_{\boldsymbol{max}}$ **and** $\boldsymbol{E}\boldsymbol{C}_{\boldsymbol{50}}$**: separate** $\boldsymbol{TOL}_{\boldsymbol{max}}$ **and** $\boldsymbol{T}\boldsymbol{C}_{\boldsymbol{50}}$

**(b) Dual tolerance on** $\boldsymbol{E}_{\boldsymbol{max}}$ **and** $\boldsymbol{E}\boldsymbol{C}_{\boldsymbol{50}}$**: shared** $\boldsymbol{TOL}_{\boldsymbol{max}}$ **and** $\boldsymbol{T}\boldsymbol{C}_{\boldsymbol{50}}$

**(c) Tolerance on** $\boldsymbol{E}_{\boldsymbol{max}}$ **only**

**(d) Tolerance on** $\boldsymbol{E}\boldsymbol{C}_{\boldsymbol{50}}$ **only**

In the Supplementary analysis, candidate tolerance structures in which tolerance was applied to $E_{max}$ and $EC_{50}$ in the pooled total model were compared.

In the structure in which $E_{max}$ and $EC_{50}$ shared the same ${TOL}_{max}$ and $TC_{50}$ values (b), $EC_{50}$ was estimated to be very low. In addition, the VPC of this structure did not adequately reproduce the observed longitudinal nausea/vomiting profile. These findings suggest that forcing tolerance effects of the same magnitude on both $E_{max}$ and $EC_{50}$ may not sufficiently describe the observed data.

Candidate models in which tolerance was applied to either $E_{max}$ or $EC_{50}$ were also evaluated. The model in which tolerance was applied only to $E_{max}$ (c) partially reproduced the observed pattern in the VPC and showed a trend similar to that of the final model. However, compared with the final model, the agreement between the observed and model-predicted medians was lower, and the overlap between the model prediction interval and the observed confidence interval was limited; therefore, it was excluded. In addition, this model estimated a substantially lower $EC_{50}$ than the final model, making the clinical interpretation of concentration sensitivity less appropriate. In the model in which tolerance was applied only to $EC_{50}$ (d), some parameter estimates were more interpretable than those from other candidate models; however, when $E_{max}$ and $EC_{50}$ estimates and VPC performance were considered together, this model was not as appropriate as the final model.

The model with separate ${TOL}_{max}$ and separate $TC_{50}$ values yielded essentially the same OFV as the final model, and $TC_{50,1}$ and $TC_{50,2}$ converged to the same estimate. The corresponding VPC (a) was also similar to that of the final model with a shared $TC_{50}.$ Thus, allowing separate $TC_{50}$ parameters provided no meaningful improvement, supporting the use of a shared $TC_{50}$ for parameter parsimony.

Therefore, the final pooled total model adopted a structure in which a separate ${TOL}_{max}$ was applied to $E_{max}$ and $EC_{50}$, while $TC_{50}$ was shared. This structure allowed the magnitude of the tolerance effect on $E_{max}$ and $EC_{50}$ to be represented separately, while maintaining a common cumulative AUC scale for tolerance development. Overall, this model was considered to provide the most appropriate balance between model flexibility and parameter parsimony.

**Supplementary 4. Structural Model for Drug Effect and Tolerance Development** [10-13, 26-27]

The non-drug effect component $f_{nd}\left( \theta;t \right)$ was described using the following functional form:

$$f_{nd}\left( \theta;t \right)=\exp\left( -\theta_{ktol}\cdot t \right)$$

Eq.1

where $\theta_{ktol}$ represents the first-order rate constant describing the temporal decay of the non-drug effect.

Equation 2 describes the drug effect applied in the obesity model:

$$f_{d}(\theta;t,C_{p},Tol)=tol(\theta;t)\cdot Slope\cdot\log(C_{p}+Intercept)$$

Eq.2

The model parameters were defined as follows:

- $Slope=\theta_{slope}$
- $Intercept=\theta_{intercept}$

The parameter $\theta_{slope}$ represents the slope of the log-linear drug effect model, and $\theta_{intercept}$ represents the intercept of the log-linear relationship.

Equation 3 describes the drug effect model applied in the total population model:

$$f_{d}\left( \theta;t, C_{p}, Tol \right)=\frac{E_{max}\cdot{tol}_{1}\left( \theta_{1};t \right)\cdot C_{p}}{{EC}_{50}\cdot{tol}_{2}\left( \theta_{2};t \right)+C_{p}}$$

Eq.3

The model parameters were defined as follows:

- $E_{max}=\theta_{Emax}$
- $EC_{50}=\theta_{EC50}$

The parameter $\theta_{Emax}$ represents the maximum drug effect on the log-hazard associated with efpeglenatide exposure, and $\theta_{EC50}$ represents the efpeglenatide concentration producing 50% of the maximum drug effect.

In both models, $C_{p}$ represents the predicted efpeglenatide concentration at time $t$, which was obtained using empirical Bayes estimates based on individual PK parameters, actual dosing history, and maximum a posteriori (MAP) Bayesian estimation.

Equation 4 represents the indirect tolerance model applied to the slope of the log concentration–linear model in the population with obesity:

$$tol(\theta;t)=1-\frac{TOL_{max}\cdot AUC(C_{p},t)}{TC_{50}+AUC(C_{p},t)}$$

Eq.4

The model parameters were defined as follows:

- $TOL_{max}=\theta_{TOL_{max\_slope}}$
- $TC_{50}=\theta_{TC50}$

Here, $AUC(C_{p},t)$ denotes the cumulative area under the concentration–time curve from time 0 to time $t$. The parameter $\theta_{TOLmax\_slope}$ represents the maximum tolerance effect on $\theta_{slope}$, and $\theta_{TC50}$ denotes the $AUC(C_{p},t)$ value producing 50% of the maximum tolerance effect.

Equation 5 represents the indirect tolerance models applied to the $E_{max}$ and $EC_{50}$ parameters in the total population $E_{max}$ model. Specifically, $tol_{1}(\theta_{1};t)$ denotes the tolerance model applied to $E_{max}$, whereas $tol_{2}(\theta_{2};t)$ denotes the tolerance model applied to $EC_{50}$:

$$tol_{1}\left( \theta_{1};t \right)=1-\frac{TOL_{max,1}\cdot AUC\left( C_{p},t \right)}{TC_{50}+AUC\left( C_{p},t \right)},$$

$$tol_{2}\left( \theta_{2};t \right)=1+\frac{TOL_{max,2}\cdot AUC(C_{p},t)}{TC_{50}+AUC(C_{p},t)}$$

Eq.5

The model parameters were defined as follows:

- $TOL_{max1}=\theta_{TOLmax1}$
- $TOL_{max2}=\theta_{TOLmax2}$
- $TC_{50}=\theta_{TC50}$

Here, $\theta_{TOLmax1}$ represents the maximum tolerance effect on $E_{max}$, and $\theta_{TOLmax2}$ represents the maximum tolerance effect on $EC_{50}$. In addition, $\theta_{TC50}$ denotes the $AUC(C_{p},t)$ value producing 50% of the maximum tolerance effect $\left( \theta_{TOLmax1} \right.$ and $\left. \theta_{TOLmax2} \right)$.

A common $TC_{50}$ was used for the $E_{max}$- and $EC_{50}$-related tolerance functions. During model development, allowing separate $TC_{50}$ parameters provided no meaningful improvement, with $TC_{50,1}$ and $TC_{50,2}$ converging to the same estimate. Therefore, a shared $TC_{50}$ was retained for parameter parsimony, while the magnitudes of the tolerance effects were allowed to differ. This parameterization was also considered biologically plausible, as the underlying mechanism of tolerance development was presumed to be shared between the $E_{max}$- and $EC_{50}$-related effects.

To ensure model stability and biologically plausible parameter estimation, the upper bounds of the tolerance parameters $\theta_{TOLmax1}$ and $\theta_{TOLmax2}$ were restricted to 1. However, the rationale for imposing these constraints differed between the two parameters.

For the tolerance model describing attenuation of $E_{max}$, at the maximum-tolerance limit, the estimated term $\theta_{Emax}\cdot(1-\theta_{TOLmax1})$ becomes negative when $\theta_{TOLmax1}$ exceeds 1, resulting in a biologically implausible negative efficacy. Therefore, the upper bound of $\theta_{TOLmax1}$ was fixed at 1 to prevent this mathematical inconsistency.

In contrast, for the tolerance model describing the increase in $EC_{50}$, represented as $\theta_{EC50}\cdot(1+\theta_{TOLmax2})$, $\theta_{TOLmax2}$ is not mathematically restricted to values below 1. Nevertheless, during model development, external factors unrelated to drug exposure but that influence the log-hazard appeared to be overestimated in the estimation of $\theta_{EC50}$, leading to model instability. Therefore, to improve model robustness and optimize parameter correlation, the upper bound for $\theta_{TOLmax2}$ was also set to 1, enabling estimation within a stable, interpretable range.

**Supplementary 5. Covariate Model Implementation**

Continuous covariates (e.g., age, BMI, HbA1c, bilirubin, and creatinine) were incorporated using a power model (Eq. 1), centered on the median value of each covariate. Categorical covariates (e.g., sex and race) were evaluated using linear models (Eq. 2) based on indicator variables. For covariates with more than two categories (e.g., race), separate parameters for each category were estimated using conditional statements implemented in the NONMEM control stream.

Continuous covariates were incorporated using power models (Eq. 1) for age, BMI, HbA1C, bilirubin, and creatinine.

$$\theta_{TV, ij}=\theta_{Ref}\cdot\left( \frac{x_{ij}}{x_{ref}} \right)^{\theta_{x}}$$

Eq. 1

Where $\theta_{Ref}$ and $\theta_{x}$ are the fixed-effect parameters, and $x_{ref}$ is the reference value for the covariates $x_{ij}$. For this analysis, the median value approximation was used for $x_{ref}$. The $\theta_{x}$ value can be either positive or negative, while $\theta_{Ref}$ must be positive numbers.

If there are two categories, the covariates were incorporated using linear models (Eq. 2), such as sex.

$$\theta_{TV, ij}=\theta_{Ref}\cdot\left( 1-x_{ij} \right)+ \theta_{x}\cdot x_{ij}$$

Eq. 2

Where $\theta_{Ref}$ and $\theta_{x}$ are the fixed-effect parameters, and $x_{ij}$ is the indicator variable which equals 1 or 0 depending on the category of the covariates. Both $\theta_{x}$ and $\theta_{Ref}$ are constrained to be positive numbers.

For covariates with more than two categories (e.g., race), the binary indicator formulation in Equation 2 was not used. In such cases, the parameter values for each category should be estimated using the IF statement in the control syntax. Each category corresponds to a separate value of $\theta.$

**Supplementary 6. Obesity model-based predicted cumulative probability and interval-specific weekly increase in nausea/vomiting occurrence by dosing scenario**

**A. Predicted cumulative probability of nausea/vomiting occurrence by week**

| **Scenario** | **Weeks** | | | | | | | | | | | | | | | | |
| --- | --- | --- | --- | --- | --- | --- | --- | --- | --- | --- | --- | --- | --- | --- | --- | --- | --- |
|  | **0** | **1** | **2** | **3** | **4** | **5** | **6** | **7** | **8** | **9** | **10** | **11** | **12** | **13** | **14** | **15** | **16** |
| **Case1** | 0.0000 | 0.3689 | 0.5531 | 0.6490 | 0.6956 | 0.7222 | 0.7408 | 0.7546 | 0.7657 | 0.7753 | 0.7837 | 0.7914 | 0.7984 | 0.8050 | 0.8111 | 0.8170 | 0.8226 |
| **Case2** | 0.0000 | 0.3689 | 0.5531 | 0.6490 | 0.6956 | 0.7222 | 0.7408 | 0.7545 | 0.7658 | 0.7754 | 0.7841 | 0.7920 | 0.7993 | 0.8061 | 0.8125 | 0.8186 | 0.8243 |
| **Case3** | 0.0000 | 0.3689 | 0.5531 | 0.6490 | 0.6956 | 0.7222 | 0.7408 | 0.7545 | 0.7658 | 0.7754 | 0.7841 | 0.7920 | 0.7993 | 0.8061 | 0.8125 | 0.8186 | 0.8244 |
| **Case4** | 0.0000 | 0.3689 | 0.5531 | 0.6490 | 0.6956 | 0.7222 | 0.7408 | 0.7545 | 0.7658 | 0.7755 | 0.7841 | 0.7920 | 0.7992 | 0.8059 | 0.8122 | 0.8182 | 0.8238 |
| **1mg weekly** | 0.0000 | 0.2769 | 0.4707 | 0.5932 | 0.6689 | 0.7187 | 0.7537 | 0.7790 | 0.7983 | 0.8139 | 0.8273 | 0.8385 | 0.8483 | 0.8570 | 0.8647 | 0.8717 | 0.8781 |
| **2mg weekly** | 0.0000 | 0.3689 | 0.5531 | 0.6447 | 0.6952 | 0.7263 | 0.7487 | 0.7663 | 0.7804 | 0.7925 | 0.8030 | 0.8124 | 0.8208 | 0.8286 | 0.8357 | 0.8423 | 0.8484 |
| **10mg weekly** | 0.0000 | 0.5280 | 0.6044 | 0.6349 | 0.6541 | 0.6691 | 0.6818 | 0.6932 | 0.7036 | 0.7132 | 0.7223 | 0.7309 | 0.7391 | 0.7469 | 0.7544 | 0.7616 | 0.7685 |
| **6mg weekly** | 0.0000 | 0.4957 | 0.6081 | 0.6499 | 0.6746 | 0.6923 | 0.7065 | 0.7188 | 0.7296 | 0.7395 | 0.7487 | 0.7572 | 0.7652 | 0.7728 | 0.7800 | 0.7869 | 0.7934 |
| **8mg weekly** | 0.0000 | 0.5165 | 0.6076 | 0.6424 | 0.6636 | 0.6796 | 0.6928 | 0.7045 | 0.7150 | 0.7247 | 0.7338 | 0.7423 | 0.7504 | 0.7581 | 0.7655 | 0.7725 | 0.7793 |

Values represent median model-predicted cumulative probabilities of nausea/vomiting occurrence up to each week from Monte Carlo simulations.

**B. Interval-specific weekly increase in predicted cumulative probability of nausea/vomiting occurrence**

| **Scenario** | **Week-interval** | | | | | | | | | | | | | | | |
| --- | --- | --- | --- | --- | --- | --- | --- | --- | --- | --- | --- | --- | --- | --- | --- | --- |
|  | **0-1** | **1-2** | **2-3** | **3-4** | **4-5** | **5-6** | **6-7** | **7-8** | **8-9** | **9-10** | **10-11** | **11-12** | **12-13** | **13-14** | **14-15** | **15-16** |
| **Case1** | 0.3689 | 0.1842 | 0.0959 | 0.0466 | 0.0266 | 0.0186 | 0.0138 | 0.0111 | 0.0096 | 0.0084 | 0.0077 | 0.0070 | 0.0066 | 0.0061 | 0.0059 | 0.0056 |
| **Case2** | 0.3689 | 0.1842 | 0.0959 | 0.0466 | 0.0266 | 0.0186 | 0.0137 | 0.0113 | 0.0096 | 0.0087 | 0.0079 | 0.0073 | 0.0068 | 0.0064 | 0.0061 | 0.0057 |
| **Case3** | 0.3689 | 0.1842 | 0.0959 | 0.0466 | 0.0266 | 0.0186 | 0.0137 | 0.0113 | 0.0096 | 0.0087 | 0.0079 | 0.0073 | 0.0068 | 0.0064 | 0.0061 | 0.0058 |
| **Case4** | 0.3689 | 0.1842 | 0.0959 | 0.0466 | 0.0266 | 0.0186 | 0.0137 | 0.0113 | 0.0097 | 0.0086 | 0.0079 | 0.0072 | 0.0067 | 0.0063 | 0.0060 | 0.0056 |
| **1mg weekly** | 0.2769 | 0.1938 | 0.1225 | 0.0757 | 0.0498 | 0.0350 | 0.0253 | 0.0193 | 0.0156 | 0.0134 | 0.0112 | 0.0098 | 0.0087 | 0.0077 | 0.0070 | 0.0064 |
| **2mg weekly** | 0.3689 | 0.1842 | 0.0916 | 0.0505 | 0.0311 | 0.0224 | 0.0176 | 0.0141 | 0.0121 | 0.0105 | 0.0094 | 0.0084 | 0.0078 | 0.0071 | 0.0066 | 0.0061 |
| **10mg weekly** | 0.5280 | 0.0764 | 0.0305 | 0.0192 | 0.0150 | 0.0127 | 0.0114 | 0.0104 | 0.0096 | 0.0091 | 0.0086 | 0.0082 | 0.0078 | 0.0075 | 0.0072 | 0.0069 |
| **6mg weekly** | 0.4957 | 0.1124 | 0.0418 | 0.0247 | 0.0177 | 0.0142 | 0.0123 | 0.0108 | 0.0099 | 0.0092 | 0.0085 | 0.0080 | 0.0076 | 0.0072 | 0.0069 | 0.0065 |
| **8mg weekly** | 0.5165 | 0.0911 | 0.0348 | 0.0212 | 0.0160 | 0.0132 | 0.0117 | 0.0105 | 0.0097 | 0.0091 | 0.0085 | 0.0081 | 0.0077 | 0.0074 | 0.0070 | 0.0068 |

Values were calculated as the difference in predicted cumulative probability between consecutive weekly time points.

**Supplementary** **7. Summary of nausea/vomiting event counts by dose and dosing regimen across the six clinical trials**

| **Study** | **Titration*** | **Regimen** | **Dose**** | **Frequency**  **of medication** | | **Number of**  **events** | | **Proportion of total events**  **within each study** |
| --- | --- | --- | --- | --- | --- | --- | --- | --- |
| HM-EXC-102 | No titration | QW | 6 mg | 4 times | | 11 | | 0.28 (11/40) |
| HM-EXC-102 | No titration | QW | 0 mg | 4 times | | 0 | | 0.00 (0/40) |
| HM-EXC-102 | 4 mg (2W),  8 mg (2W) | QM | 16 mg | 3 times | | 28 | | 0.70 (28/40) |
| HM-EXC-102 | No titration | QM | 0 mg | 3 times | | 1 | | 0.03 (1/40) |
| HM-EXC-201 | No titration | Single | 2 mcg/kg | 1 time | | 1 | | 0.08 (1/12) |
| HM-EXC-201 | No titration | Single | 4 mcg/kg | 1 time | | 1 | | 0.08 (1/12) |
| HM-EXC-201 | No titration | Single | 8 mcg/kg | 1 time | | 0 | | 0.00 (0/12) |
| HM-EXC-201 | No titration | Single | 14 mcg/kg | 1 time | | 0 | | 0.00 (0/12) |
| HM-EXC-201 | No titration | Single | 20 mcg/kg | 1 time | | 1 | | 0.08 (1/12) |
| HM-EXC-201 | No titration | Single | 40 mcg/kg | 1 time | | 1 | | 0.08 (1/12) |
| HM-EXC-201 | No titration | Single | 60 mcg/kg | 1 time | | 4 | | 0.33 (4/12) |
| HM-EXC-201 | No titration | Single | 100 mcg/kg | 1 time | | 4 | | 0.33 (4/12) |
| HM-EXC-201 | No titration | Single | 0 mcg/kg | 1 time | | 0 | | 0.00 (0/12) |
| HM-EXC-202 | No titration | QW | 1 mg | 8 times | | 0 | | 0.00 (0/79) |
| HM-EXC-202 | No titration | QW | 2 mg | 8 times | | 3 | | 0.04 (3/79) |
| HM-EXC-202 | No titration | QW | 4 mg | 8 times | | 5 | | 0.06 (5/79) |
| HM-EXC-202 | No titration | QM | 8 mg | 3 times | | 21 | | 0.27 (21/79) |
| HM-EXC-202 | No titration | QM | 12 mg | 3 times | | 16 | | 0.20 (16/79) |
| HM-EXC-202 | No titration | QM | 16 mg | 3 times | | 19 | | 0.24 (19/79) |
| HM-EXC-202 | No titration | QM | 0 mg | 3 times | | 15 | | 0.19 (15/79) |
| HM-EXC-203 | No titration | QW | 0.3 mg | 12 times | | 3 | | 0.09 (3/32) |
| HM-EXC-203 | No titration | QW | 1 mg | 12 times | | 1 | | 0.03 (1/32) |
| HM-EXC-203 | No titration | QW | 2 mg | 12 times | | 7 | | 0.22 (7/32) |
| HM-EXC-203 | No titration | QW | 3 mg | 12 times | | 7 | | 0.22 (7/32) |
| HM-EXC-203 | No titration | QW | 4 mg | 12 times | | 8 | | 0.25 (8/32) |
| HM-EXC-203 | No titration | QW | 0 mg | 12 times | | 6 | | 0.19 (6/32) |
| HM-EXC-204 | 4 mg (4W),  8 mg (1W) | QM | 8 mg | 2 times | | 24 | | 0.25 (24/95) |
| HM-EXC-204 | 4 mg (4W),  8 mg (1W) | QM | 12 mg | 2 times | | 30 | | 0.32 (30/95) |
| HM-EXC-204 | 4 mg (4W),  8 mg (1W) | QM | 16 mg | 2 times | | 37 | | 0.39 (37/95) |
| HM-EXC-204 | No titration | QM | 0 mg | 2 times | | 4 | | 0.04 (4/95) |
| HM-EXC-205 | No titration | QW | 4 mg | 20 times | | 88 | | 0.21 (88/414) |
| HM-EXC-205 | No titration | QW | 6 mg | 20 times | | 99 | | 0.24 (99/414) |
| HM-EXC-205 | No titration | QW | 0 mg | 20 times | | 0 | | 0.00 (0/ 414) |
| HM-EXC-205 | 4 mg (2W) | Q2W | 6 mg | 9 times | | 83 | | 0.20 (83/414) |
| HM-EXC-205 | 4 mg (2W) | Q2W | 8 mg | 9 times | | 126 | | 0.30 (126/414) |
| HM-EXC-205 | No titration | Q2W | 0 mg | 9 times | | 18 | | 0.04 (18/414) |
| Overall | | | | | 672 | | - | |
| 1W = once weekly (one time), 2W = once weekly (two times), 4W = once weekly (four times)  QW = once weekly, QM = once monthly, Q2W = once every other week  The proportion of events was calculated using the total number of nausea/vomiting events within each study as the denominator, not the number of participants.  *Titration was performed weekly.  **The dose of 0mg corresponds to the placebo group. | | | | | | | | |

**Supplementary 8. Comparison of obesity model bootstrap summaries according to replicate inclusion criteria**

| **Replicate inclusion criteria** | **N** | **Percentile** | $\boldsymbol{\theta}_{\mathbf{0}}$ | $\boldsymbol{\theta}_{\boldsymbol{TOL}_{\boldsymbol{max}\boldsymbol{\_slope}}}$ | $\boldsymbol{\theta}_{\boldsymbol{TC}_{\boldsymbol{50}}}$ | $\boldsymbol{\theta}_{\boldsymbol{k}\boldsymbol{tol}}$ | $\boldsymbol{\theta}_{\boldsymbol{Slope}}$ | $\boldsymbol{\theta}_{\boldsymbol{Intercept}}$ |
| --- | --- | --- | --- | --- | --- | --- | --- | --- |
| **All replicates** | 1000 | 2.5 | 0.0000641 | 0.996 | 109,872 | 0.00219 | 0.303 | 0.000702 |
|  |  | 50 | 0.000169 | 0.996 | 109,872 | 0.0191 | 0.592 | 0.397 |
|  |  | 97.5 | 0.000351 | 0.996 | 109,872 | 0.0263 | 0.825 | 3.138 |
| **Minimization successful**  **only** | 957 (0.957) | 2.5 | 0.0000635 | 0.996 | 109,872 | 0.00222 | 0.301 | 0.000702 |
|  |  | 50 | 0.000167 | 0.996 | 109,872 | 0.0191 | 0.596 | 0.419 |
|  |  | 97.5 | 0.000351 | 0.996 | 109,872 | 0.0263 | 0.826 | 3.259 |
| **Evaluable**  **replicates** | 997 (0.997) | 2.5 | 0.0000640 | 0.996 | 109,872 | 0.00219 | 0.302 | 0.000702 |
|  |  | 50 | 0.000169 | 0.996 | 109,872 | 0.0191 | 0.592 | 0.398 |
|  |  | 97.5 | 0.000351 | 0.996 | 109,872 | 0.0263 | 0.825 | 3.147 |
| **Final estimates** | - | | 0.000153 | 0.996 | 109,,872 | 0.0208 | 0.627 | 0.634 |

Values are presented as bootstrap percentiles. Evaluable replicates were defined as bootstrap runs with successful minimization or minimization terminated with rounding errors that yielded parameter estimates. Among 1000 bootstrap replicates, 957 showed successful minimization, 40 terminated with rounding errors, and 3 were non-evaluable. $\theta_{0}$, baseline hazard; $\theta_{{TOL}_{max\_slope}}$, maximum tolerance effect on *Slope*; $\theta_{{TC}_{50}}$, cumulative AUC producing 50% of maximum tolerance effect corresponding to *Slope*; $\theta_{ktol}$, first-order rate constant describing the time course of the non-drug effect; $\theta_{Slope}$, slope of the log-linear concentration–effect relationship; $\theta_{Intercept}$, intercept of the log-linear concentration–effect relationship.

**Supplementary 9. Visual predictive checks from sensitivity analyses of the log-linear concentration–effect intercept in the primary obesity model**

**(a) Final estimate for the intercept,** $\boldsymbol{\theta}_{\boldsymbol{Intercept}}$**= 0.634 ng/mL**

**(b) Lower bound of 95% bootstrap confidence bound for the intercept,** $\boldsymbol{\theta}_{\boldsymbol{Intercept}}$**= 0.0000702 ng/mL**

**(c) Median of the bootstrap estimates for the intercept,** $\boldsymbol{\theta}_{\boldsymbol{Intercept}}$**= 0.419 ng/mL**

**(d) Upper bound of 95% bootstrap confidence bound for the intercept,** $\boldsymbol{\theta}_{\boldsymbol{Intercept}}$**= 3.259 ng/mL**

The bootstrap median and 95% confidence interval values used for this sensitivity analysis were based on bootstrap replicates with successful minimization only (N=957), whereas the bootstrap summary in Table 5 was based on evaluable replicates.

**Supplementary 10. Comparison of total model bootstrap summaries according to replicate inclusion criteria**

| **Replicate inclusion criteria** | **N** | **Percentile** | $\theta_{0}$ | $\theta_{{TOL}_{{max}_{1}}}$ | $\theta_{{TC}_{50}}$ | $\theta_{E_{max}}$ | $\theta_{{EC}_{50}}$ | $\theta_{ktol}$ | $\theta_{{TOL}_{{max}_{2}}}$ |
| --- | --- | --- | --- | --- | --- | --- | --- | --- | --- |
| **All replicates** | 1000 | 2.5 | 0.0000381 | 0.616 | 109,872 | 2.674 | 0.000833 | 0.00405 | 0.996 |
|  |  | 50 | 0.000068 | 0.824 | 109,872 | 3.625 | 0.102 | 0.00902 | 0.996 |
|  |  | 97.5 | 0.000122 | 0.996 | 109,872 | 8.481 | 310.089 | 0.0147 | 0.996 |
| **Minimization successful**  **only** | 769 (0.769) | 2.5 | 0.0000388 | 0.621 | 109,872 | 2.664 | 0.000833 | 0.00407 | 0.996 |
|  |  | 50 | 0.0000691 | 0.827 | 109,872 | 3.942 | 3.870 | 0.00955 | 0.996 |
|  |  | 97.5 | 0.000124 | 0.996 | 109,872 | 8.620 | 320.376 | 0.0149 | 0.996 |
| **Evaluable**  **replicates** | 993  (0.993) | 2.5 | 0.0000381 | 0.616 | 109,872 | 2.672 | 0.00083 | 0.00404 | 0.996 |
|  |  | 50 | 0.0000674 | 0.824 | 109,872 | 3.622 | 0.102 | 0.00901 | 0.996 |
|  |  | 97.5 | 0.000122 | 0.996 | 109,872 | 8.472 | 307.315 | 0.0147 | 0.996 |
| **Final estimates** | - | | 0.0000702 | 0.829 | 109,872 | 5.22 | 81 | 0.0117 | 0.996 |

Values are presented as bootstrap percentiles. Evaluable replicates were defined as bootstrap runs with successful minimization or minimization terminated with rounding errors that yielded parameter estimates. Among 1000 bootstrap replicates, 769 showed successful minimization, 224 terminated with rounding errors, and 7 were non-evaluable. $\theta_{0}$, baseline hazard; $\theta_{{TOL}_{{max}_{1}}}$ and $\theta_{{TOL}_{{max}_{2}}}$ represent the maximum tolerance effects on $\theta_{E_{max}}$ and $\theta_{{EC}_{50}}$, respectively; $\theta_{{TC}_{50}}$, cumulative AUC producing 50% of maximum tolerance effect; $\theta_{ktol}$, first-order rate constant describing the time course of the non-drug effect; ; $\theta_{E_{max}}$ represents the maximum drug effect on the log-hazard; $\theta_{{EC}_{50}}$ is the efpeglenatide concentration producing 50% of the maximum drug effect; ;

**Supplementary 11. Primary obesity model–based cumulative occurrence and event-free probability profiles across nine dosing scenarios**

Due to the number of figure panels, Supplementary 11 is provided as a separate PDF file. The PDF contains the model-predicted cumulative probability of nausea/vomiting and the corresponding event-free probability for each of the nine dosing scenarios based on the primary obesity model.

**Supplementary 12. Exploratory pooled total model–based simulations of cumulative nausea/vomiting occurrence and event-free probability across nine dosing scenarios**

**(a) Total model-predicted cumulative probability of nausea/vomiting across nine dosing scenarios**

**(b) Total model-predicted event-free probability of nausea/vomiting across nine dosing scenarios**

**Supplementary 13. Simulated cumulative AUC trajectories across nine dosing scenarios with TC50 and TC90 tolerance thresholds**

**(a) Cumulative AUC trajectories up to 16 weeks with the TC90 threshold**

**(b) Early cumulative AUC trajectories up to 8 weeks with the TC50 threshold**

Simulated cumulative AUC trajectories are shown for the nine dosing scenarios. The horizontal dashed lines indicate the cumulative AUC thresholds corresponding to TC50 and TC90, defined as the cumulative AUC required to reach 50% and 90% of the maximum tolerance effect, respectively.

**Supplementary 14. Comparison of AUC-driven, time-driven, and no-tolerance model structures in the obesity model**

**(a)** **Candidate tolerance structures and corresponding hazard equations evaluated for the primary obesity model**

| **Candidate tolerance structure** | **Tolerance function** | **Hazard equation** |
| --- | --- | --- |
| **AUC driven- tolerance**  **(Log-linear/*Slope*; Final model)** | $Tol(AUC)=\frac{\theta_{TOLmax}\cdot AUC}{\theta_{TC50}+AUC}$ | $h(t)=\theta_{0}\cdot\exp\left\{ \exp(-\theta_{ktol}\cdot t)+\theta_{slope}\cdot(1-Tol(AUC))\cdot\log(C_{p}+\theta_{intercept}) \right\}$ |
| **Time-drive -Tolerance**  **(Log-linear/*Slope*)** | $Tol(t)=\frac{\theta_{TOLmax}\cdot t}{\theta_{TC50}+t}$ | $h(t)=\theta_{0}\cdot\exp\left\{ \exp(-\theta_{ktol}\cdot t)+\theta_{slope}\cdot(1-Tol(t))\cdot log(C_{p}+\theta_{intercept}) \right\}$ |
| **Time-drive -Tolerance**  **(**$\boldsymbol{E}_{\boldsymbol{max}}$**/**$\boldsymbol{E}_{\boldsymbol{max}}$**)** |  | $h(t)=\theta_{0}\cdot\exp\left\{ \exp(-\theta_{ktol}\cdot t)+\frac{\theta_{Emax}\cdot(1-Tol(t))\cdot C_{p}}{\theta_{EC50}+C_{p}} \right\}$ |
| **Time-drive -Tolerance**  **(**$\boldsymbol{E}_{\boldsymbol{max}}$**/**$\boldsymbol{E}\boldsymbol{C}_{\boldsymbol{50}}$**)** |  | $h(t)=\theta_{0}\cdot\exp\left\{ \exp(-\theta_{ktol}\cdot t)+\frac{\theta_{Emax}\cdot C_{p}}{\theta_{EC50}\cdot(1+Tol(t))+C_{p}} \right\}$ |
| **No tolerance**  **(**$\boldsymbol{E}_{\boldsymbol{max}}$**/ Neither** $\boldsymbol{E}_{\boldsymbol{max}}$ **nor** $\boldsymbol{E}\boldsymbol{C}_{\boldsymbol{50}}$**)** | — | $h(t)=\theta_{0}\cdot\exp\left\{ \exp(-\theta_{ktol}\cdot t)+\frac{\theta_{Emax}\cdot C_{p}}{\theta_{EC50}+C_{p}} \right\}$ |
| **No tolerance**  **(Log-linear/*Slope*)** | — | $h(t)=\theta_{0}\cdot\exp\left\{ \exp(-\theta_{ktol}\cdot t)+\theta_{slope}\cdot log(C_{p}+\theta_{intercept}) \right\}$ |

Where AUC, cumulative area under the concentration–time curve; $C_{p}$, time-varying drug concentration; $h(t)$, hazard at time $t$; $\theta_{0}$ denotes the baseline hazard; $\theta_{ktol}$ is the first-order rate constant describing the time course of the non-drug effect; $\theta_{slope}$ is the slope of the log-linear concentration–effect relationship; $\theta_{intercept}$ is the intercept of the log-linear concentration–effect model; $\theta_{E_{max}}$ denotes the maximum drug effect in the Emax concentration–effect model; $\theta_{{EC}_{50}}$ denotes the concentration producing 50% of Emax in the Emax concentration–effect model; $\theta_{{TOL}_{max}}$ represents the maximum tolerance effect; and $\theta_{{TC}_{50}}$denotes the cumulative AUC or treatment time producing 50% of the maximum tolerance effect. For log-linear concentration–effect models, tolerance was applied to the concentration–effect slope. For Emax-based concentration–effect models, tolerance was applied to either Emax or EC50, as specified in the column heading. A dash indicates that the parameter was not included in the corresponding model

The first row represents the final AUC-driven tolerance model used as the reference obesity model in the main analysis. In this model, the drug effect was described using a log-linear concentration–effect relationship, and tolerance development was driven by cumulative AUC. The second row represents a time-driven tolerance model with the same log-linear concentration–effect structure, in which tolerance was driven by treatment time rather than cumulative AUC. The third and fourth rows represent $E_{max}$-based time-driven tolerance models in which tolerance was applied to $E_{max}$ or $EC_{50}$, respectively. The fifth row represents an $E_{max}$ concentration–effect model without a tolerance component, and the sixth row represents a log-linear concentration–effect model without a tolerance component. These alternative structures were compared with the final AUC-driven tolerance model to evaluate whether cumulative AUC provided additional explanatory value beyond treatment time alone or no tolerance component.

**(b) Parameter estimates for AUC-driven, time-driven, and no-tolerance candidate model structures evaluated for the primary obesity model**

| **Obesity model** | | | | | | |
| --- | --- | --- | --- | --- | --- | --- |
| **Parameter** | **AUC driven- tolerance**  **(Log-linear/*Slope*; Final model)** | **Time-drive -Tolerance**  **(Log-linear/*Slope*)** | **Time-drive -Tolerance**  **(**$\boldsymbol{E}_{\boldsymbol{max}}$**/**$\boldsymbol{E}_{\boldsymbol{max}}$**)** | **Time-drive -Tolerance**  **(**$\boldsymbol{E}_{\boldsymbol{max}}$**/**$\boldsymbol{E}\boldsymbol{C}_{\boldsymbol{50}}$**)** | **No tolerance**  **(**$\boldsymbol{E}_{\boldsymbol{max}}$**/ Neither** $\boldsymbol{E}_{\boldsymbol{max}}$ **nor** $\boldsymbol{E}\boldsymbol{C}_{\boldsymbol{50}}$**)** | **No tolerance**  **(Log-linear/*Slope*)** |
| **OFV** | 6650.932 | 6678.365 | 6363.698 | 6906.729 | 6906.740 | 6961.332 |
| **ΔOFV vs Final** | reference | +27.433 | -287.234 | +255.797 | +255.808 | +310.400 |
| **Base model: Exponential model** | | | | | | |
| $\theta_{0}$ | 0.000153 hr^-1^ | 0.000168 hr^-1^ | 0.0000582 hr^-1^ | 0.0000661 hr^-1^ | 0.0000661 hr^-1^ | 0.000245 hr^-1^ |
| **Drug effect (log-linear concentration model)** | | | | | | |
| $\theta_{Slope}$ | 0.627 (hr$\cdot$ng/mL)^-1^ | 1.30 (hr$\cdot$ng/mL)^-1^ | - | - | - | 0.0764 (hr$\cdot$ng/mL)^-1^ |
| $\theta_{Intercept}$ | 0.634 ng/mL | 0. 000702 ng/mL | - | - | - | 0.000702 ng/mL |
| **Drug effect (Emax concentration model)** | | | | | | |
| $\theta_{E_{max}}$ | - | - | 6.76 | 1.83 | 1.83 |  |
| $\theta_{{EC}_{50}}$ | - | - | 0.000702 ng/mL | 0.000702 ng/mL | 0.000702 ng/mL |  |
| **Tolerance effect** | | | | | | |
| $\theta_{{TOL}_{max}}$ | 0.996 | 0.964 | 0.813 | 0.996 | - | - |
| $\theta_{{TC}_{50}}$ | 109,872 hr$\cdot$ng/mL | 20.832 hr (0.124 week) | 16.128 hr (0.096 week) | 162.12 hr (0.965 week) | - | - |
| **Non-drug effect** | | | | | | |
| $\theta_{ktol}$ | 0.0208 hr^-1^ | 0.00133 hr^-1^ | 0.000887 hr^-1^ | 0.00165 hr^-1^ | 0.00165 hr^-1^ | 0.00169 hr^-1^ |

Where OFV is objective function value; AUC, cumulative area under the concentration–time curve; $\theta_{0}$ denotes the baseline hazard; $\theta_{ktol}$ is the first-order rate constant describing the time course of the non-drug effect; $\theta_{slope}$ is the slope of the log-linear concentration–effect relationship; $\theta_{intercept}$ is the intercept of the log-linear concentration–effect model; $\theta_{E_{max}}$ denotes the maximum drug effect in the Emax concentration–effect model; $\theta_{{EC}_{50}}$ denotes the concentration producing 50% of Emax in the Emax concentration–effect model; $\theta_{{TOL}_{max}}$ represents the maximum tolerance effect; and $\theta_{{TC}_{50}}$denotes the cumulative AUC or treatment time producing 50% of the maximum tolerance effect. For log-linear concentration–effect models, tolerance was applied to the concentration–effect slope. For Emax-based concentration–effect models, tolerance was applied to either Emax or EC50, as specified in the column heading. A dash indicates that the parameter was not included in the corresponding model

The first column represents the final AUC-driven tolerance model used as the reference obesity model in the main analysis. In this model, the drug effect was described using a log-linear concentration–effect relationship, and tolerance development was driven by cumulative AUC. The second column represents a time-driven tolerance model with the same log-linear concentration–effect structure, in which tolerance was driven by treatment time rather than cumulative AUC. The third and fourth columns represent $E_{max}$-based time-driven tolerance models in which tolerance was applied to $E_{max}$ or $EC_{50}$, respectively. The fifth column represents an $E_{max}$ concentration–effect model without a tolerance component, and the sixth column represents a log-linear concentration–effect model without a tolerance component. These alternative structures were compared with the final AUC-driven tolerance model to evaluate whether cumulative AUC provided additional explanatory value beyond treatment time alone or no tolerance component.

**Supplementary 15. Visual predictive checks comparing AUC-driven, time-driven, and no-tolerance structures in the obesity model**

**(a) Final obesity model: AUC-driven tolerance on *Slope* in the log-linear concentration-effect model**

**(b) Time-driven tolerance on *Slope* in the log-linear concentration-effect model**

**(c) Time-driven tolerance on** $\boldsymbol{E}_{\boldsymbol{max}}$ **in the Emax concentration-effect model**

**(d) Time-driven tolerance on** $\boldsymbol{E}\boldsymbol{C}_{\boldsymbol{50}}$ **in the Emax concentration-effect model**

**(e) The** $\boldsymbol{E}_{\boldsymbol{max}}$ **concentration-effect model without tolerance**

**(f) The Log-linear concentration-effect model without tolerance**

Visual predictive checks are shown for the final AUC-driven tolerance model and five alternative candidate models evaluated in the obesity dataset. The final model used a log-linear concentration–effect structure with cumulative AUC-driven tolerance applied to the concentration–effect slope. Alternative models included time-driven tolerance applied to the log-linear slope, time-driven tolerance applied to either $E_{max}$ or $EC_{50}$ in $E_{max}$-based concentration–effect models, and models without a tolerance component. Although the time-driven Emax-tolerance model yielded a lower OFV than the final model, it was not retained because its VPC was less consistent with the observed early longitudinal pattern and its parameter estimates, particularly the very small EC50 and very early TC50, were less pharmacologically interpretable. The final AUC-driven model most adequately reproduced the observed longitudinal nausea/vomiting profile, including the rapid early increase and subsequent attenuation/plateau phase.

**Supplementary 16. Comparison of candidate exposure–hazard models for the obesity and pooled total datasets**

| **Dataset** | **Candidate**  **model** | $\boldsymbol{T}\boldsymbol{C}_{\boldsymbol{50}}$ **(hr·ng/mL)** | **OFV** | **VPC performance** | **Interpretation** | **Decision** |
| --- | --- | --- | --- | --- | --- | --- |
| Obesity | Log-linear | 109,872 | 6650.932 | Better reproduced rapid early increase and subsequent attenuation/plateau | Best longitudinal  predictive adequacy | Final |
| Obesity | $E_{max}$ | 109,872 | 6457.511 | Less adequate VPC despite  lower OFV than the log-linear mode | Lower OFV but  poorer time-course prediction | Not selected |
| Total | Log-linear | 109,872 | 10718.258 | Comparable/slightly better VPC  and Lower OFV than the $E_{max}$ model | Less pharmacologically interpretable  for concentration threshold | Not selected |
| Total | $E_{max}$ | 109,872 | 10782.399 | Acceptable/comparable VPC and  Slightly higher OFV  than the log-linear model | Retained for complementary  ${EC}_{50}$/$E_{max}$-based  exploratory interpretation | Exploratory  final |

$TC_{50}$ denotes the cumulative AUC associated with 50% of the maximum tolerance effect; OFV, objective function value.; VPC performance was evaluated by visual agreement with the observed cumulative probability profiles.

Candidate models were evaluated by considering OFV, VPC performance, longitudinal predictive adequacy, and pharmacological interpretability. For the obesity model, the log-linear structure was selected as the final model despite a higher OFV than the Emax structure, because it provided better VPC fit and more adequately reproduced the longitudinal cumulative probability profile. The Emax model in the pooled total dataset was retained for complementary exploratory $EC_{50}$- and $E_{max}$-based interpretation.

**Supplementary 17. Visual predictive checks supporting exposure–hazard model selection in the obesity and pooled total datasets**

**(a) VPC for the candidate** $\boldsymbol{E}_{\boldsymbol{max}}$**-exposure–hazard model in the obesity dataset**

**(b) VPC for the candidate log-linear exposure–hazard model in the pooled total dataset**

**Supplementary 18. Individual PK-derived cumulative AUC trajectories and week 4 exposure-quartile cutoffs**

Each line represents an individual PK-derived cumulative AUC trajectory in active-treatment patients. The vertical dashed line indicates week 4, and the horizontal dashed lines indicate the cumulative AUC quartile cutoffs at week 4. These profiles show substantial interindividual variability in cumulative exposure despite the same treatment duration.

**Supplementary 19. Summary of observed nausea/vomiting events according to week 4 individual PK-derived cumulative AUC quartiles**

| **Exposure quartile** | **Week 4 cumulative AUC range (h·ng/mL)** | **N patients** | **N events up to week 4** | **Events per patient** | **Proportion of total events (%)** | **Observed cumulative probability at week1** | **Observed cumulative probability at week2** | **Observed cumulative probability at week4** |
| --- | --- | --- | --- | --- | --- | --- | --- | --- |
| Q1 (25%) | 178190.6 ~ 254013.1 | 51 | 41 | 0.80 | 18.3 | 0.31264 | 0.46828 | 0.56398 |
| Q2 (50%) | 254013.1 ~ 306098.4 | 50 | 62 | 1.24 | 27.7 | 0.42940 | 0.61224 | 0.71437 |
| Q3 (75%) | 306098.4 ~ 371717.1 | 50 | 53 | 1.06 | 23.7 | 0.39538 | 0.57344 | 0.65731 |
| Q4 (100%) | 371717.1 ~ 625470.3 | 51 | 68 | 1.33 | 30.4 | 0.46905 | 0.65651 | 0.7416 |
| Total | - | 202 | 224 | 1.11 | 100 | - | - | - |

Note. “Exposure quartiles” were defined based on individual PK-derived cumulative AUC at week 4 among active-treatment patients. The cumulative AUC range is expressed as h·ng/mL. “N events up to week 4” represents the total number of observed nausea/vomiting events occurring up to week 4 within each quartile, and “events per patient” was calculated as the number of events divided by the number of patients in each quartile. “The proportion of total events” was calculated relative to the total number of nausea/vomiting events observed up to week 4. “Observed cumulative probabilities” were estimated using the Kaplan–Meier method.

**Supplementary 20. Observed cumulative probability of nausea/vomiting during total follow up time stratified by week 4 PK-derived cumulative AUC quartiles**

Kaplan–Meier–based observed cumulative probability curves are shown over the total follow-up period according to week 4 exposure quartiles. The vertical dashed line marks week 4, and the horizontal dashed lines indicate the observed cumulative probability at week 4 for each group stratified by week 4 PK-derived cumulative AUC quartiles. Higher week 4 cumulative AUC quartiles (Q4) generally showed greater cumulative occurrence probability (0.742) in week 4. This analysis was used to compare the magnitude of observed cumulative occurrence probability across exposure quartiles, including the corresponding probabilities at week 4.

**Supplementary 21. Observed cumulative probability of nausea/vomiting during week4-interval stratified by week 4 PK-derived cumulative AUC quartiles**

Kaplan–Meier–based observed cumulative probability curves are presented for nausea/vomiting events from treatment initiation through week 4, with week 4 serving as the endpoint of the early-interval analysis. Patients were stratified by individual PK-derived cumulative AUC quartiles at week 4. This complementary early-interval analysis was used to compare the relative timing and accumulation pattern of events observed within the first 4 weeks across the exposure quartiles. Higher exposure quartiles, particularly Q4 relative to Q1, generally showed earlier accumulation of observed events.

**Supplementary 22. Supporting Evidence for GLP-1 Receptor Agonist Titration Schedules and Estimated 50% Tolerance Timing in Table 8**

**(a) Liraglutide**

Liraglutide 3.0 mg was evaluated as a weight-management therapy in a pivotal clinical trial involving patients with overweight or obesity, in which gastrointestinal adverse events, including nausea and diarrhea, were commonly reported [54]. The gastric-emptying delay associated with liraglutide 3.0 mg may represent one of the mechanisms contributing to appetite regulation and early weight loss; however, this effect may gradually attenuate with sustained GLP-1 receptor stimulation. According to Maselli et al. (2022), the gastric-emptying delay observed at week 5 was significantly reduced by week 16 (−12.9 min, p < 0.001), suggesting drug-related tachyphylaxis or physiological adaptation [55].

In addition, according to the clinical data analyzed by Lean et al., 62% of nausea episodes occurred within the first 4 weeks, and 67% of vomiting episodes occurred within the first 6 weeks, indicating that these events were concentrated during the standard dose-escalation period (weeks 1–4) and the immediately subsequent period [52]. The FDA Summary Review also noted that treatment discontinuations due to adverse events were concentrated during the initial 8 weeks, suggesting that this period may represent a clinically relevant transition window for determining whether tolerability is established [53].

Taken together, the period from approximately day 14 after treatment initiation, when attenuation of the gastric-emptying delay begins, to approximately weeks 4–8, when nausea/vomiting occurrence is concentrated and begins to attenuate, may represent a clinically relevant window during which approximately 50% tolerance to nausea/vomiting develops and physiological adaptation becomes established. Accordingly, by week 8, corresponding to completion of the 4-week titration period followed by an additional 4 weeks of treatment, tolerability may be broadly established at the population level, with adverse-event management entering a more stable phase.

**(b) Semaglutide**

The gastric-emptying delay induced by semaglutide also appears to attenuate progressively with continued treatment, which may reflect physiological adaptation associated with tolerance development. In a study in which semaglutide was escalated every 4 weeks over a 16-week dose-escalation period to the target dose, followed by approximately 4 weeks of maintenance treatment, no evidence of delayed gastric emptying was observed at week 20. This observation is consistent with the physiological adaptation typically associated with long-acting GLP-1 receptor agonist therapy [56].

Furthermore, pooled analyses of the STEP 1–4 trials showed that gastrointestinal adverse events occurred predominantly during the dose-escalation period, approximately within the first 16–20 weeks, and tended to stabilize after attainment of the target dose [8]. In particular, the prevalence of nausea or vomiting peaked during the 16-20 weeks and subsequently declined, whereas the proportion of patients experiencing their first gastrointestinal adverse event reached a plateau after approximately the week 20 [8]. Similarly, the time-to-event profiles for semaglutide 2.4 mg showed rapid accumulation of both first and recurrent gastrointestinal events during approximately the first 20 weeks, followed by marked attenuation in the rate of further event accumulation [57]. Additionally, during dose escalation period, treatment discontinuation due to gastrointestinal adverse events was relatively more frequent [8]. Also, the FDA review similarly reported that gastrointestinal adverse events were most frequently observed during dose escalation [57]. These findings suggest that the latter phase of dose escalation and the transition to maintenance dosing may represent a clinically relevant window for tolerability development and patient retention.

Taken together, these findings suggest that approximately 50% tolerance to semaglutide-associated nausea or vomiting may be established during the late dose-escalation to early maintenance period, approximately between weeks 16 and 20.

**(c) Dulaglutide**

Dulaglutide, consistent with the known class effects of GLP-1 receptor agonists, is associated with gastrointestinal adverse events such as nausea or vomiting, particularly during the early phase of treatment, with events generally concentrated within the first 2 weeks after initiation. This early pattern is thought to be related, at least in part, to the rapid onset of delayed gastric emptying, whereas sustained exposure may promote gradual tolerance development through physiological adaptation, potentially involving receptor desensitization or vagal nervous system adaptation. [38].

In a PK/PD Markov chain model–based simulation analysis, immediate initiation of higher dulaglutide doses, such as 3.0 or 4.5 mg, was predicted to result in peak nausea or vomiting incidences within 2–4 weeks, followed by stabilization toward a steady-state phase at approximately week 18. In contrast, when a stepwise dose-escalation regimen was applied at 4-week intervals to reach 4.5 mg after at least 12 weeks, the incidences of nausea or vomiting peaked at approximately week 12 and subsequently stabilized as shown in Figure 3 of Tang CC et al [10]. The estimated half-lives for tolerance development were approximately 43 days for nausea and 49 days for vomiting [10].

Considering the relationship between the dose-escalation period and tolerance development, the estimated time required to achieve 50% tolerance for nausea or vomiting was approximately 6–7 weeks, which was shorter than the time required to reach the 3.0 and 4.5 mg maintenance doses, approximately 8 and 12 weeks, respectively. This finding suggests that the currently recommended 4-week dose-escalation interval may provide sufficient time for clinically meaningful tolerance development before patients are exposed to higher doses. Accordingly, by the end of dose escalation, a substantial degree of tolerance is expected to have developed, allowing adverse-event management to enter a more stable phase.

**(d) Tirzepatide**

Tirzepatide has been shown to attenuate its gastric-emptying delay effect over time after repeated dosing in healthy participants. In a once-weekly dosing study in healthy participants, the gastric-emptying delay effect was significantly diminished by Day 23, corresponding to approximately 3.3 weeks after treatment initiation [58]. In addition, in participants with type 2 diabetes mellitus, gastric-emptying delay diminished over 4 weeks; near-complete tachyphylaxis was observed with repeated 5-mg dosing, whereas residual gastric-emptying delay remained at Day 23 in the 5/5/10/10-mg and 5/5/10/15-mg dose-escalation cohorts [58]. Collectively, these findings suggest that physiological adaptation or tolerance to tirzepatide-induced gastric-emptying delay may begin to emerge within approximately 3–4 weeks after treatment initiation.

Gastrointestinal adverse events, including nausea and diarrhea, have generally been reported to occur most frequently during the approximately 20-week dose-escalation period [59]. The FDA Summary Review likewise noted that gastrointestinal adverse events were most frequently reported during dose escalation, and gastrointestinal disorders were the most common adverse events leading to treatment discontinuation. [60].

In addition, the cumulative incidence curves presented in Figure 3 of the FDA Summary review showed marked attenuation in the slopes of the nausea or vomiting curves after approximately weeks 16–24 [60]. These findings suggest that, around completion of the approximately 20-week dose-escalation period, the likelihood of new-onset gastrointestinal adverse events may decrease.

In addition, Figure 13 of FDA Clinical Pharmacology Review showed that, in the 15 mg treatment arm, the prevalence of nausea reached its highest levels during the late dose-escalation period and subsequently decreased and stabilized during continued maintenance treatment [26]. These patterns are consistent with substantial physiological adaptation emerging around weeks 16–24, spanning the late dose-escalation and early maintenance periods.

Overall, based on the temporal attenuation in nausea/vomiting occurrence and prevalence, approximately 50% tolerance to nausea or vomiting was inferred to develop between weeks 16 and 20.

**References**

1. Müller, T. D., Finan, B., Bloom, S. R., D’Alessio, D., Drucker, D. J., Flatt, P. R., et al. (2019). Glucagon-like peptide 1 (GLP-1). Mol. Metab. 30, 72–130. doi:10.1016/j.molmet.2019.09.010
2. Drucker, D. J. (2022). GLP-1 physiology informs the pharmacotherapy of obesity. Mol. Metab. 57, 101351. doi:10.1016/j.molmet.2021.101351
3. Holst, J. J. (2007). The physiology of glucagon-like peptide 1. Physiol. Rev. 87 (4), 1409–1439. doi:10.1152/physrev.00034.2006
4. Reid, J., Rana, K., Segel, S. A., Sheikh-Ali, M., Choksi, R. R., and Goldfaden, R. F. (2019). Efpeglenatide. Glucagon-like peptide 1 (GLP-1) receptor agonist, treatment for type 2 diabetes. Drugs Future 44 (6), 435–441. doi:10.1358/dof.2019.44.6.2927591
5. Pratley, R. E., Kang, J., Trautmann, M. E., Hompesch, M., Han, O., Stewart, J., et al. (2019). Body weight management and safety with efpeglenatide in adults without diabetes: a phase II randomized study. Diabetes, Obes. Metabolism 21 (11), 2429–2439. doi:10.1111/dom.13824
6. Rosenstock, J., Sorli, C., Trautmann, M. E., Morales, C., Wendisch, U., Dailey, G., et al. (2019). Once-weekly efpeglenatide dose-range effects on glycemic control and body weight in patients with type 2 diabetes on metformin or drug naive, referenced to liraglutide. Diabetes Care 42 (9), 1733–1741. doi:10.2337/dc18-2648
7. Yoon, K.-H., Kang, J., Kwon, S. C., Trautmann, M. E., Hompesch, M., Stewart, J., et al. (2020). Pharmacokinetic and dose-finding studies on efpeglenatide in patients with type 2 diabetes. Diabetes, Obes. Metabolism 22 (8), 1292–1301. doi:10.1111/dom.14032
8. Wharton S, Calanna S, Davies M, Dicker D, Goldman B, Lingvay I, Mosenzon O, Rubino DM, Thomsen M, Wadden TA, Pedersen SD. Gastrointestinal tolerability of once-weekly semaglutide 2.4 mg in adults with overweight or obesity, and the relationship between gastrointestinal adverse events and weight loss. Diabetes Obes Metab. 2022a;24:94–105. doi:10.1111/dom.14551
9. Jastreboff AM, Aronne LJ, Ahmad NN, Wharton S, Connery L, Alves B, Kiyosue A, Zhang S, Liu B, Bunck MC, Stefanski A. Tirzepatide once weekly for the treatment of obesity. N Engl J Med. 2022;387:205–216.
10. Tang CC, Lim J, Loo LS, Jung H, Konig M, Tham LS. Practical applications of a nausea and vomiting model in the clinical development of additional doses of dulaglutide. J Clin Pharmacol. 2024;64:215–226.
11. Niebecker R, Maas H, Staab A, Freiwald M, Karlsson MO. Modeling Exposure-Driven Adverse Event Time Courses in Oncology Exemplified by Afatinib. *CPT Pharmacometrics Syst Pharmacol* (2013) 2:e70. doi: 10.1038/psp.2013.46
12. Li, M., Bhatt, D.L., Kankam, M., Ambery, P. and Bhatt, D. (2024) 'Exposure–response modeling for nausea incidence for cotadutide, a dual GLP-1/glucagon receptor agonist', *CPT: Pharmacometrics & Systems Pharmacology*, 13, pp. 1968–1979. doi: 10.1002/psp4.13194
13. Karlsson, K. E., Plan, E. L., and Karlsson, M. O. (2011). Performance of Three Estimation Methods in Repeated Time-to-Event Modeling. The AAPS Journal 13(1), 83–91. doi:10.1208/s12248-010-9248-3.
14. Zingmark, P.H., Ekblom, M., Odergren, T., Ashwood, T., Lyden, P., Karlsson, M.O. and Jonsson, E.N. (2005) 'Modelling a spontaneously reported side effect by use of a Markov mixed-effects model', European Journal of Clinical Pharmacology, 61(2), pp. 157–164. doi: 10.1007/s00228-005-0917-8
15. Karlsson, M.O., Jonsson, E.N. and Grahnén, A. (2019) 'Covariates in pharmacometric repeated time-to-event models', Journal of Pharmacokinetics and Pharmacodynamics, 46(1), pp. 79–93. doi: 10.1007/s10928-018-09617-6
16. Del Prato, S., Kang, J., Trautmann, M. E., Stewart, J., Sorli, C. H., Derwahl, M., et al. (2020). Efficacy and safety of once-monthly efpeglenatide in patients with type 2 diabetes: results of a phase 2 placebo-controlled, 16-week randomized dose-finding study. Diabetes, Obes. Metabolism 22 (7), 1176–1186. doi:10.1111/dom.14020
17. Hompesch, M.,Kang, J.,Han,O. P., Trautmann, M. E., Sorli,C.H., Ogbaa, I., et al. (2021). Effects of efpeglenatide versus liraglutide on gastric emptying, glucose metabolism and beta-cell function in people with type 2 diabetes: an exploratory randomized phase Ib study. BMJ Open Diabetes Res. and Care 9 (1), e002208. doi:10.1136/bmjdrc-2021-002208
18. Choi, S., Seo, J., Park, S., Kim, N.Y., Kim, H. and Lim, H.-S. (2025) 'Population pharmacokinetics of efpeglenatide in individuals with obesity and with type 2 diabetes', *Frontiers in Pharmacology*, 16, p. 1715585. doi: 10.3389/fphar.2025.1715585
19. Seo, J. (2024). Refining Repeated Time-to-Event Models Across Different Scenarios and Implementing Them in NONMEM®. Master’s thesis. Ulsan, Republic of Korea: Ulsan University. Available at: https://oak.ulsan.ac.kr/handle/2021.oak/13301
20. Seo, J., and Lim, H.-S. (2026). The refinement of repeated exact time-to-event modeling: methodological refinements and implementation in NONMEM®—Part I. Front. Pharmacol. 17:1791423. doi: 10.3389/fphar.2026.1791423
21. Beal, S.L., Sheiner, L.B., Boeckmann, A.J. and Bauer, R.J. (eds) (1989–2020) *NONMEM 7.5 Users Guides*. ICON plc, Gaithersburg, MD.
22. Lindstrom, M. J., and Bates, D. M. (1990). Nonlinear mixed effects models for repeated measures data. Biometrics 46 (3), 673–687. doi: 10.2307/2532087
23. Bauer RJ. NONMEM tutorial part I: Description of commands and options, with simple examples of population analysis. CPT Pharmacometrics Syst Pharmacol. 2019a;8:525–537. doi:10.1002/psp4.12351
24. Bauer RJ. NONMEM tutorial part II: Estimation methods and advanced examples. CPT Pharmacometrics Syst Pharmacol. 2019b;8:538–556. doi:10.1002/psp4.12352
25. R Core Team (2022). R: A Language and Environment for Statistical Computing. Vienna: R Foundation for Statistical Computing. Available online at: https://www.R-project.org/ (accessed December 2, 2024).
26. U.S. Food and Drug Administration. (2023a). Clinical Pharmacology Review: Zepbound (tirzepatide). Silver Spring, MD: U.S. Food and Drug Administration, Center for Drug Evaluation and Research.
27. Gabrielsson, J. and Weiner, D. (2016). Pharmacokinetic and Pharmacodynamic Data Analysis: Concepts and Applications. 5th ed. Swedish Pharmaceutical Press, Stockholm.
28. Jonsson, E.N. and Karlsson, M.O. (1998) 'Automated covariate model building within NONMEM', Pharmaceutical Research, 15(9), pp. 1463–1468. doi: 10.1023/A:1011970125687
29. Ette, E.I. (1997). Stability and performance of a population pharmacokinetic model. Journal of Clinical Pharmacology, 37(6), 486–495. <https://doi.org/10.1002/j.1552-4604.1997.tb04347.x>
30. Bonate, P.L. (2011). Pharmacokinetic-Pharmacodynamic Modeling and Simulation. 2nd ed. Springer, New York. <https://doi.org/10.1007/978-1-4419-9485-1>
31. Lindbom L, Pihlgren P, Jonsson EN. PsN-Toolkit — a collection of computer intensive statistical methods for non-linear mixed effect modeling using NONMEM. Comput Methods Programs Biomed. 2005;79(3):241–257. doi: 10.1016/j.cmpb.2005.04.005
32. Karlsson, M. O., and Holford, N. (2008). A tutorial on visual predictive checks. PAGE 17, Abstr. 1434. Available at: https://www.page-meeting.org/?abstract=1434
33. Bergstrand, M., Hooker, A.C., Wallin, J.E. and Karlsson, M.O. (2011). Prediction-corrected visual predictive checks for diagnosing nonlinear mixed-effects models. The AAPS Journal, 13(2), 143–151. <https://doi.org/10.1208/s12248-011-9255-z>
34. Lalonde, R.L., Kowalski, K.G., Hutmacher, M.M., et al. (2007). Model-based drug development. Clinical Pharmacology & Therapeutics, 82(1), 21–32.
35. Ooi, Q. X., Plan, E., and Bergstrand, M. (2025). A tutorial on pharmacometric Markov models. CPT Pharmacometrics Syst Pharmacol. 14, 197–216. doi:10.1002/psp4.13278.
36. Drucker, D.J. (2018) Mechanisms of action and therapeutic application of glucagon-like peptide-1, *Cell Metabolism*, 27(4), pp. 740–756.
37. Holst, J. J. (2024). GLP-1 physiology in obesity and development of incretin-based drugs for chronic weight management. Nat. Metab. 6, 1866–1885. doi: 10.1038/s42255-024-01113-9
38. Nauck, M.A., Kemmeries, G., Holst, J.J. and Meier, J.J. (2011) Rapid tachyphylaxis of the glucagon-like peptide 1-induced deceleration of gastric emptying in humans, *Diabetes*, 60(5), pp. 1561–1565.
39. Jalleh, R.J., Plummer, M.P., Marathe, C.S., Umapathysivam, M.M., Quast, D.R., Rayner, C.K., Jones, K.L., Wu, T., Horowitz, M. and Nauck, M.A. (2025b) Clinical consequences of delayed gastric emptying with GLP-1 receptor agonists and tirzepatide, *Journal of Clinical Endocrinology & Metabolism*, 110(1), pp. 1–15. doi:10.1210/clinem/dgae719
40. Khanna D, Khanna S, Khanna P, Kahar P, Patel BM. Obesity: A Chronic Low-Grade Inflammation and Its Markers. Cureus. 2022;14(2):e22711. doi:10.7759/cureus.22711
41. Cani PD, Bibiloni R, Knauf C, Waget A, Neyrinck AM, Delzenne NM, et al. Changes in Gut Microbiota Control Metabolic Endotoxemia-Induced Inflammation in High-Fat Diet–Induced Obesity and Diabetes in Mice. Diabetes. 2008;57(6):1470–1481. doi:10.2337/db07-1403
42. Miron I, Dumitrascu DL. Gastrointestinal Motility Disorders in Obesity. Acta Endocrinol (Buchar). 2019;15(4):497–504. doi:10.4183/aeb.2019.497
43. Emerenziani S, Guarino MPL, Trillo Asensio LM, Altomare A, Ribolsi M, Balestrieri P, et al. Role of Overweight and Obesity in Gastrointestinal Disease. Nutrients. 2020;12(1):111. doi:10.3390/nu12010111
44. Faasse K, Petrie KJ. The nocebo effect: patient expectations and medication side effects. Postgrad Med J. 2013;89(1055):540–546. doi:10.1136/postgradmedj-2012-131730
45. Petrie KJ, Rief W. Psychobiological Mechanisms of Placebo and Nocebo Effects: Pathways to Improve Treatments and Reduce Side Effects. Annu Rev Psychol. 2019;70:599–625. doi:10.1146/annurev-psych-010418-102907
46. Colloca L, Miller FG. The nocebo effect and its relevance for clinical practice. Psychosom Med. 2011;73(7):598–603. doi:10.1097/PSY.0b013e3182294a50
47. Jalleh, R. J., Marathe, C. S., Rayner, C. K., Jones, K. L., Umapathysivam, M. M., Wu, T., et al. (2025a). Physiology and pharmacology of effects of GLP-1-based therapies on gastric, biliary and intestinal motility. Endocrinology 166, bqae155. doi: 10.1210/endocr/bqae155.
48. Bettge, K., Kahle, M., Abd El Aziz, M.S., Meier, J.J. and Nauck, M.A. (2017) Occurrence of nausea, vomiting and diarrhoea reported as adverse events in clinical trials studying glucagon-like peptide-1 receptor agonists: a systematic analysis of published clinical trials, *Diabetes, Obesity and Metabolism*, 19(3), pp. 336–347.
49. Gorgojo-Martínez, J.J., Mezquita-Raya, P., Carretero-Gómez, J., Castro, A., Cebrián-Cuenca, A., de Torres-Sánchez, A., García-de-Lucas, M.D., Núñez, J., Obaya, J.C., Soler, M.J., Górriz, J.L. and Rubio-Herrera, M.Á. (2023) Clinical recommendations to manage gastrointestinal adverse events in patients treated with GLP-1 receptor agonists: a multidisciplinary expert consensus, *Journal of Clinical Medicine*, 12(1), article 145.
50. Wharton, S., Davies, M., Dicker, D., Lingvay, I., Mosenzon, O., Rubino, D.M. and Pedersen, S.D. (2022b) Managing the gastrointestinal side effects of GLP-1 receptor agonists in obesity: recommendations for clinical practice, *Postgraduate Medicine*, 134(1), pp. 14–19. doi:10.1080/00325 481.2021.2002616
51. Horowitz, M., Aroda, V.R., Han, J., Hardy, E. and Rayner, C.K. (2017) 'Upper and/or lower gastrointestinal adverse events with glucagon-like peptide-1 receptor agonists: Incidence and consequences', Diabetes, Obesity and Metabolism, 19(5), pp. 672–681.
52. Lean MEJ, Carraro R, Finer N, Hartvig H, Lindegaard ML, Rössner S, Van Gaal L, Astrup A. Tolerability of nausea and vomiting and associations with weight loss in a randomized trial of liraglutide in obese, non-diabetic adults. Int J Obes. 2014;38:689–697.
53. U.S. Food and Drug Administration (2014). Summary Review for Regulatory Action: Saxenda (Liraglutide). Silver Spring, MD: U.S. Food and Drug Administration, Center for Drug Evaluation and Research.
54. Pi-Sunyer, X., Astrup, A., Fujioka, K., Greenway, F., Halpern, A., Krempf, M., Edelson, G., Jensen, C.B., Boardman, M.K., Davies, M. and Wilding, J.P.H. (2015) A randomized, controlled trial of 3.0 mg of liraglutide in weight management, New England Journal of Medicine, 373(1), pp. 11–22.
55. Maselli D, Atieh J, Clark MM, Eckert D, Taylor A, Carlson P, Burton DD, Busciglio I, Harmsen WS, Vella A, Acosta A, Camilleri M. Effects of liraglutide on gastrointestinal functions and weight in obesity: A randomized clinical and pharmacogenomic trial. Obesity (Silver Spring). 2022;30:1608–1620.
56. Friedrichsen M, Breitschaft A, Tadayon S, Wizert A, Skovgaard D. The effect of semaglutide 2.4 mg once weekly on energy intake, appetite, control of eating, and gastric emptying in adults with obesity. Diabetes Obes Metab. 2021;23:754–762.
57. U.S. Food and Drug Administration (2021). Division Director Summary Review for Regulatory Action: Wegovy (Semaglutide). Silver Spring, MD: U.S. Food and Drug Administration, Center for Drug Evaluation and Research.
58. Urva S, Coskun T, Loghin C, Cui X, Beebe E, O’Farrell L, Briere DA, Benson C, Nauck MA, Haupt A. The novel dual glucose-dependent insulinotropic polypeptide and glucagon-like peptide-1 receptor agonist tirzepatide transiently delays gastric emptying similarly to selective long-acting GLP-1 receptor agonists. Diabetes Obes Metab. 2020;22:1886–1891.
59. Rubino, D.M. et al. (2025) Gastrointestinal tolerability and weight reduction associated with tirzepatide in adults with obesity or overweight with and without type 2 diabetes in the SURMOUNT-1 to -4 trials, Diabetes, Obesity and Metabolism, 27(4), pp. 1826–1835. DOI: 10.1111/dom.16176
60. U.S. Food and Drug Administration. (2023b). Cross-Discipline Team Leader Summary Review for Regulatory Action: Zepbound (tirzepatide). Silver Spring, MD: U.S. Food and Drug Administration, Center for Drug Evaluation and Research.
